# Supplementary figures and images for: Detection of genes with differential expression dispersion unravels the role of autophagy in cancer progression
Source: PLoS Comput Biol. 2023 Mar 9;19(3):e1010342. doi: 10.1371/journal.pcbi.1010342 (PMC9997931; doi:10.1371/journal.pcbi.1010342)

**A**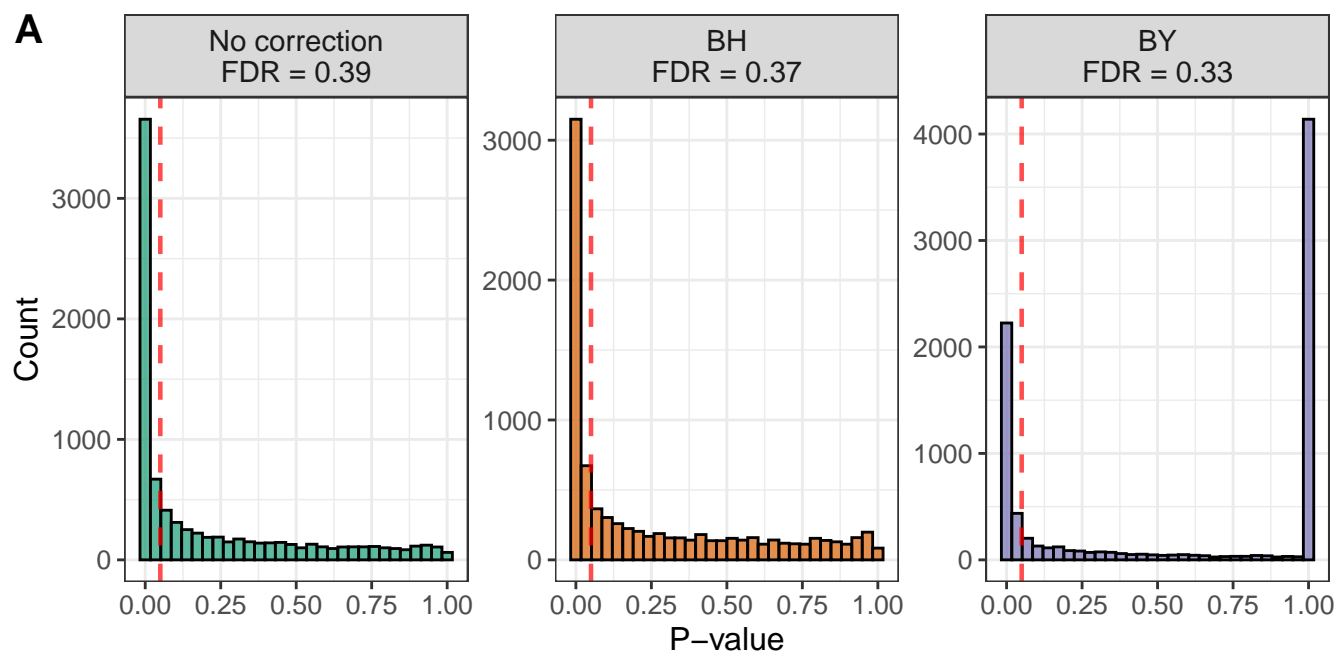**B**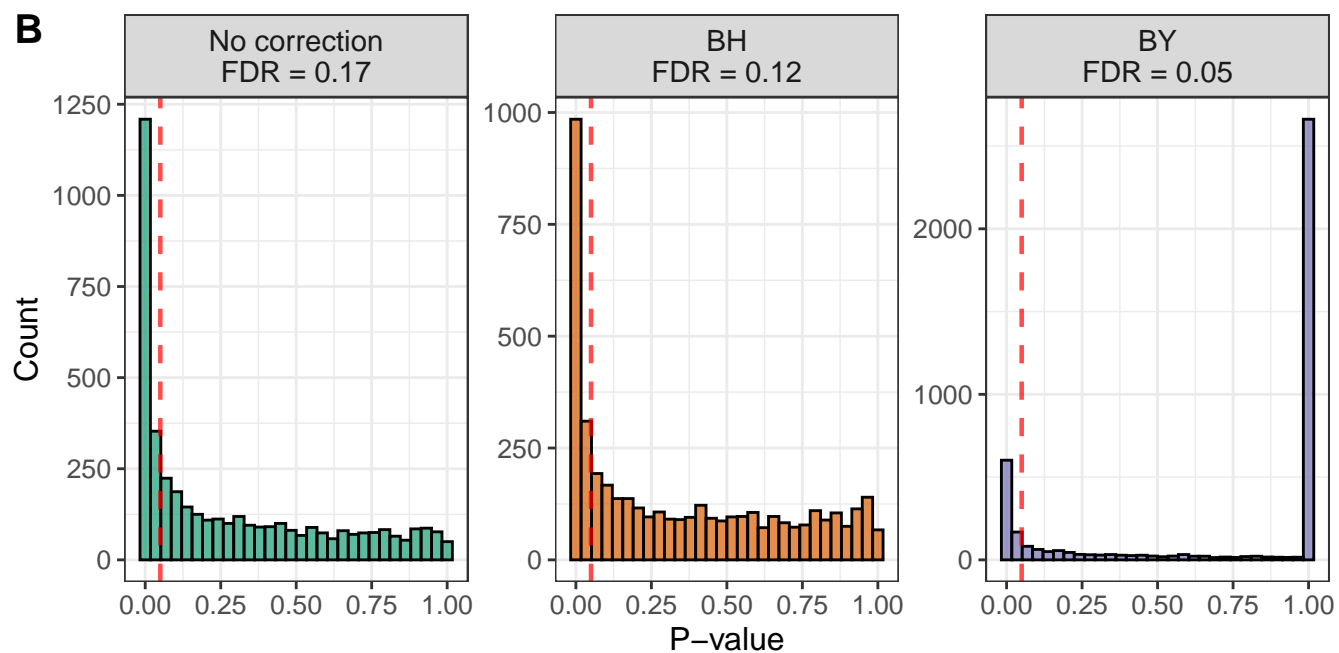

Supplement: S1 Fig — P-values obtained with MDSeq for the detection of differential dispersion in gene expression data from a simulated dataset composed of highly and lowly differentially expressed genes between two populations of 50 samples. P-values were corrected by the Benjamini-Hochberg (BH) and Benjamini-Yekutieli (BY) procedures for (A) the entire set of genes and (B) the lowly DE genes only. The red dotted lines represent a p-value threshold value of 0.05. (PDF) [file pcbi.1010342.s001.pdf]

Performance

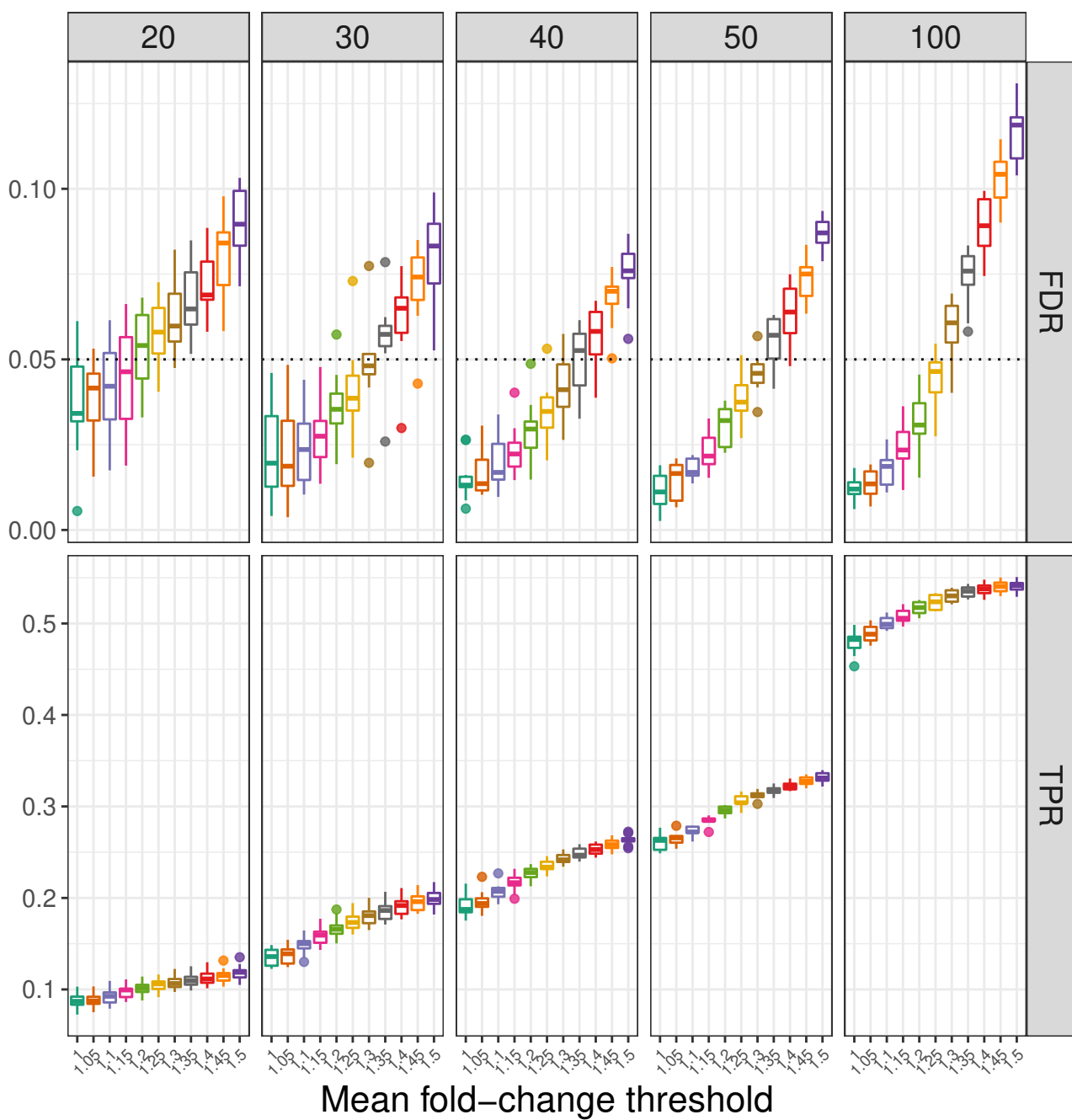

Supplement: S3 Fig — (A) False discovery rate (FDR) and (B) true positive rate (TPR) values obtained with simulated datasets composed of two sample populations of equal size (panels on the horizontal axis). (PDF) [file pcbi.1010342.s003.pdf]

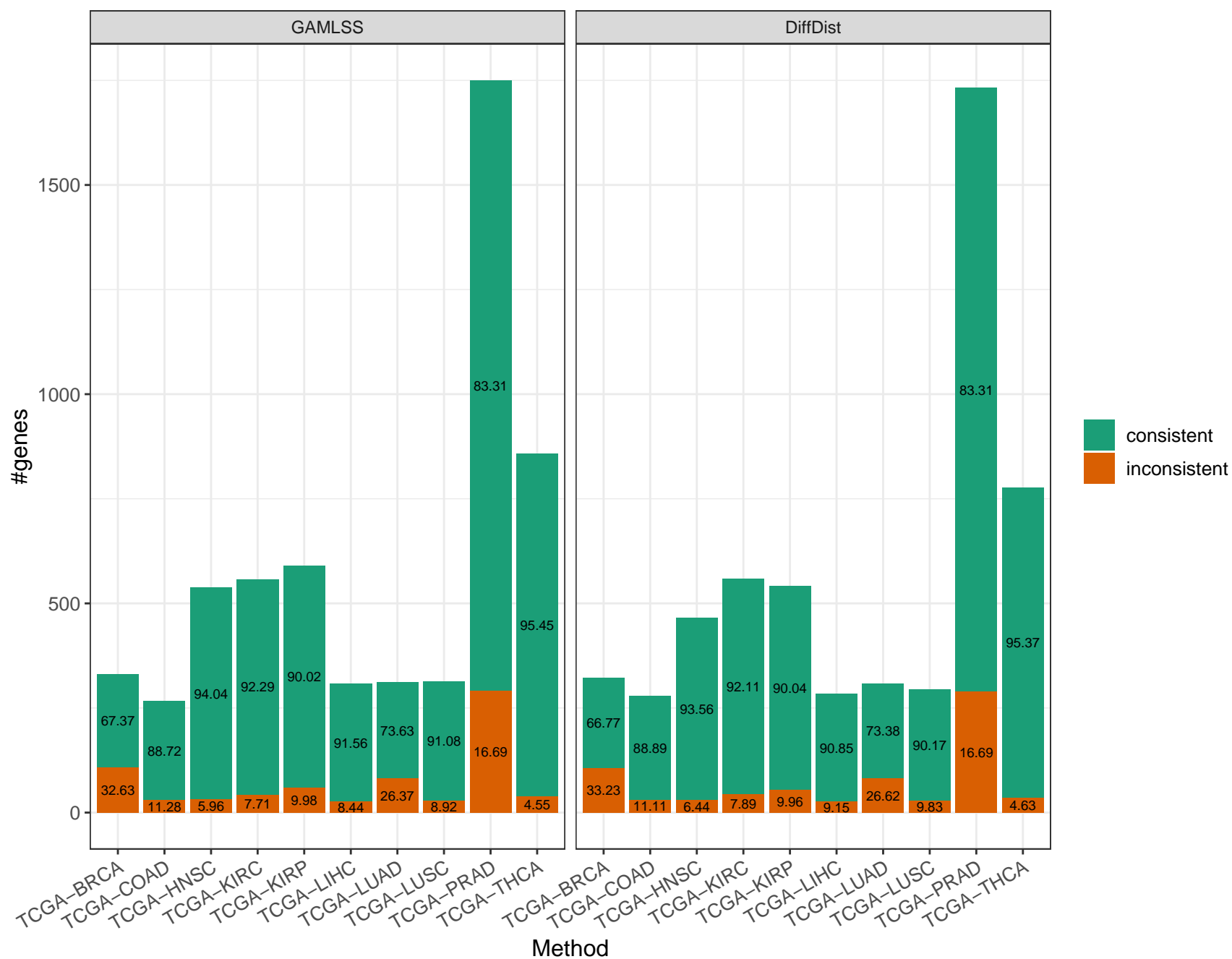

Supplement: S4 Fig — Counts of differentially dispersed (DD) genes specifically identified by (A) GAMLSS or (B) DiffDist with a sign of dispersion log2-fold change consistent or inconsistent with the one predicted by DiPhiSeq for all the analyzed TCGA datasets. Percentages of the two defined categories of genes over the entire sets of specifically identified DD genes are indicated at the middle of the corresponding bars. (PDF) [file pcbi.1010342.s004.pdf]

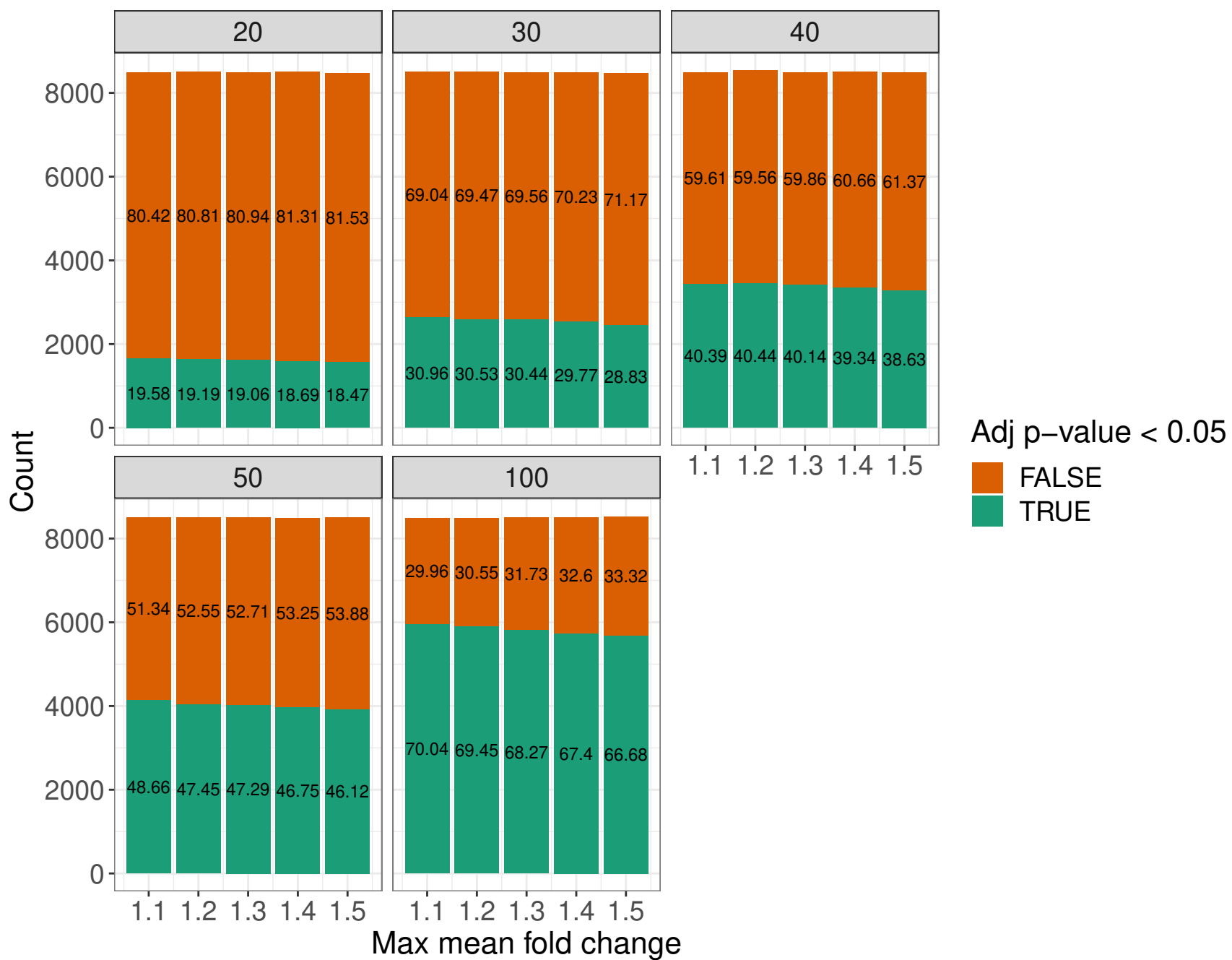

Supplement: S6 Fig — The Shapiro-Wilk test was computed for each gene after log2-transformation of the normalized read counts. P-values were corrected by the Benjamini-Hochberg procedure and displayed stratified by the sample size. Counts of adjusted p-values lower than 0.05 and adjusted p-values greater than 0.05 were averaged over 10 replicates of simulated datasets and percentages are indicated at the middle of the corresponding bars. (PDF) [file pcbi.1010342.s006.pdf]

Samples    ● tumor    ● normal

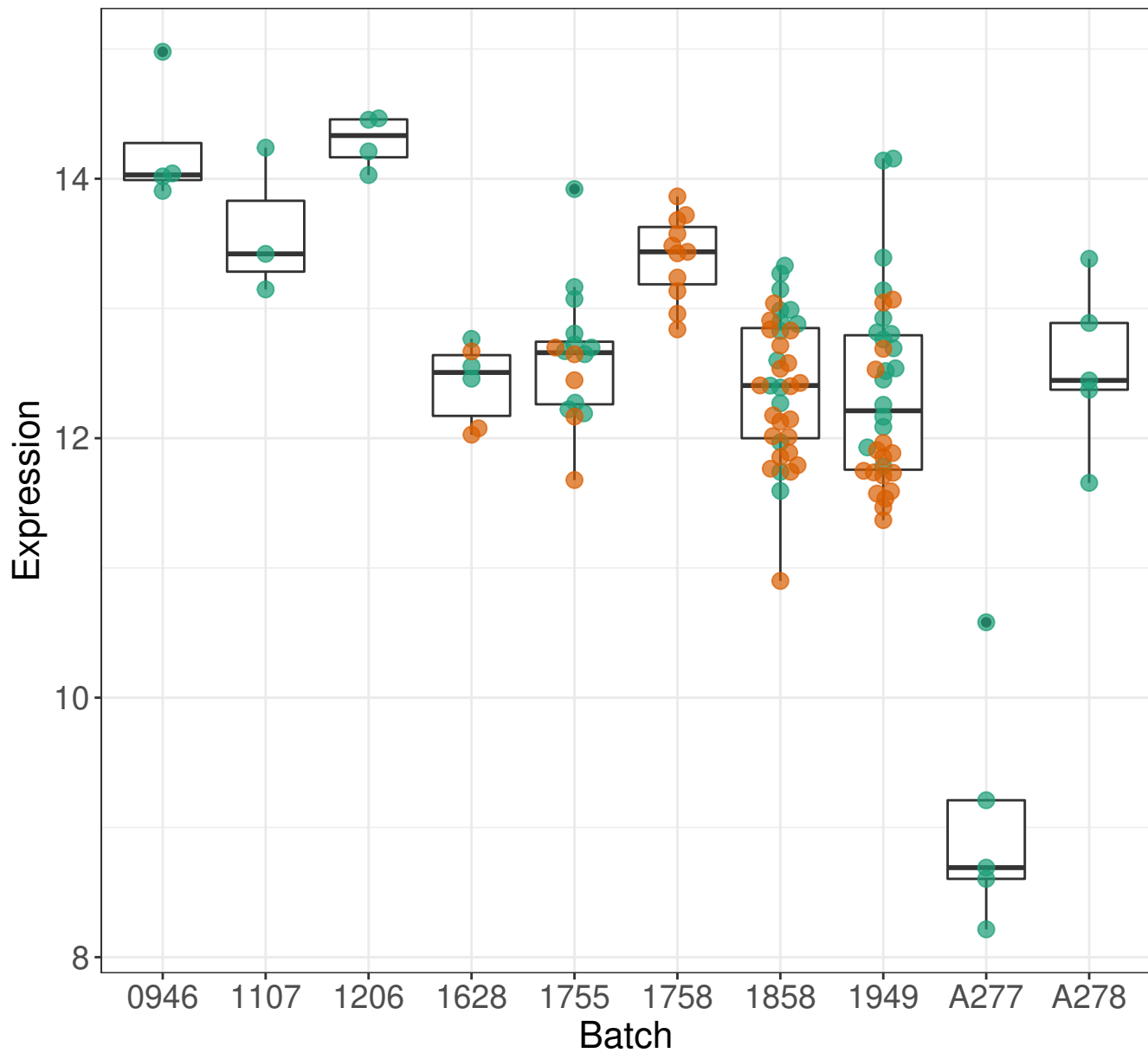

Supplement: S7 Fig — Expression values of the LMAN2 gene (lectin, mannose binding 2, ENSG00000169223) based on the TCGA dataset composed of samples from patients with lung adenocarcinoma (TCGA-LUAD) for whom both a tumor sample and a normal sample are available. Data are clustered according to sequencing batch. In batches 0946, 1107, 1206, A277 and A278, which enabled the sequencing of only tumor samples, the dispersion of LMAN2 expression increased with respect to the other batches composed of samples from both conditions. Corrected p-values obtained with the five evaluated methods are listed below. MDSeq without the integration of batch effect by a blocking factor in the generalized linear model (GLM): 2.1410−4, MDSeq with the integration of batch effect by a blocking factor in the GLM: 1.1410−1, Levene’s test: 1.9510−3, DiPhiSeq: 9.0810−5, GAMLSS: 2.9410−8, DiffDist: 1.4810−4. (PDF) [file pcbi.1010342.s007.pdf]

Sample size: 20

**A**

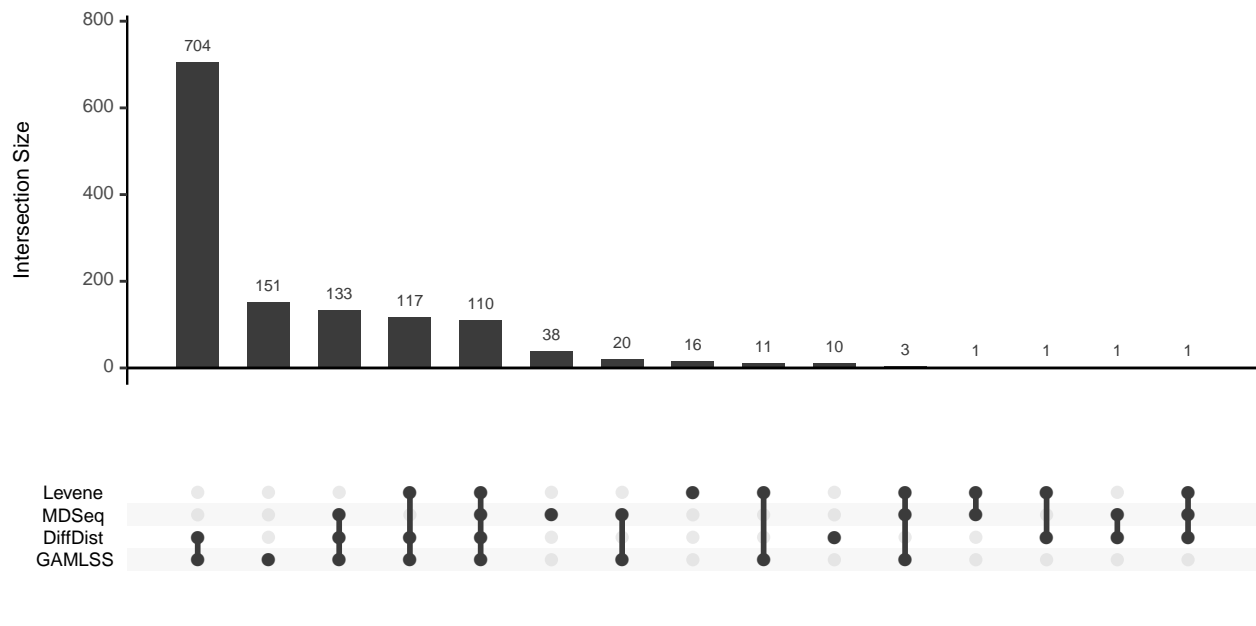

**B**

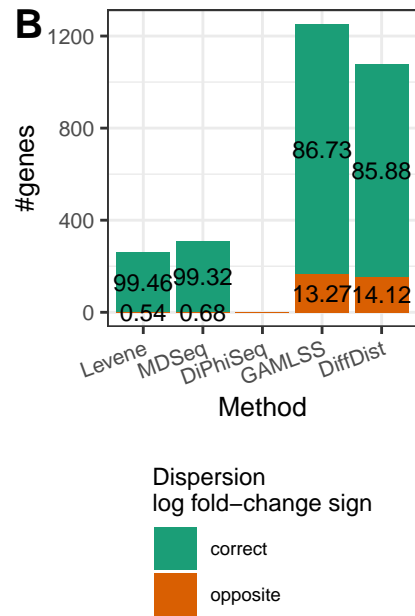

**C**

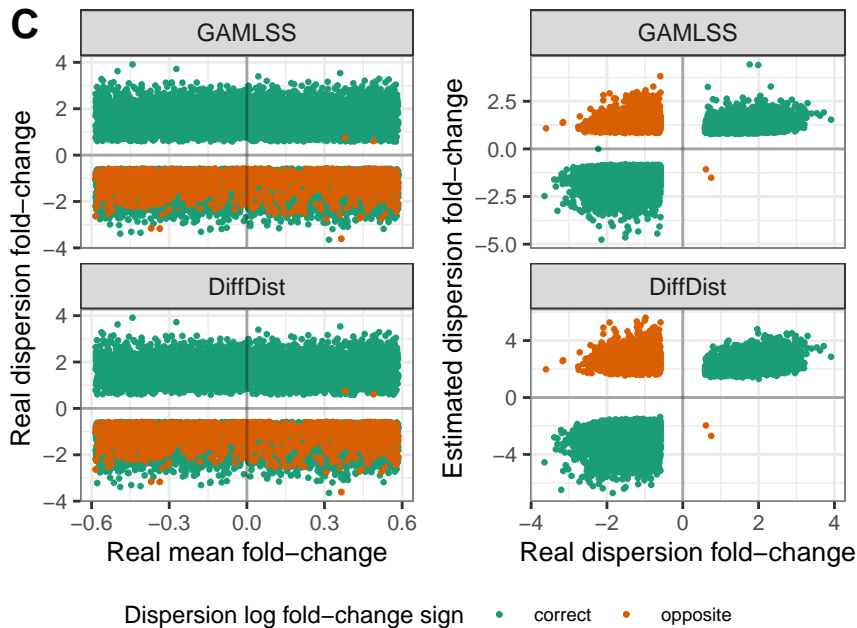

**D**

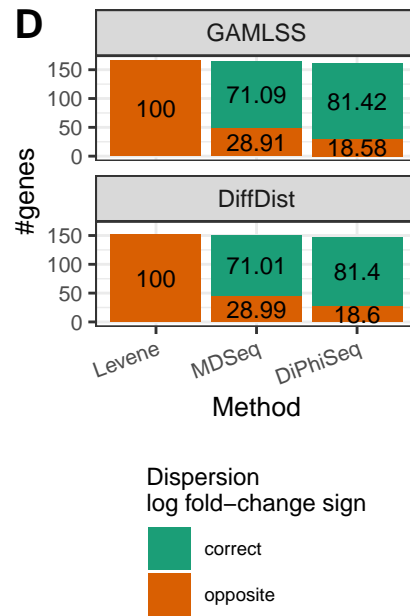

Sample size: 30

**A**

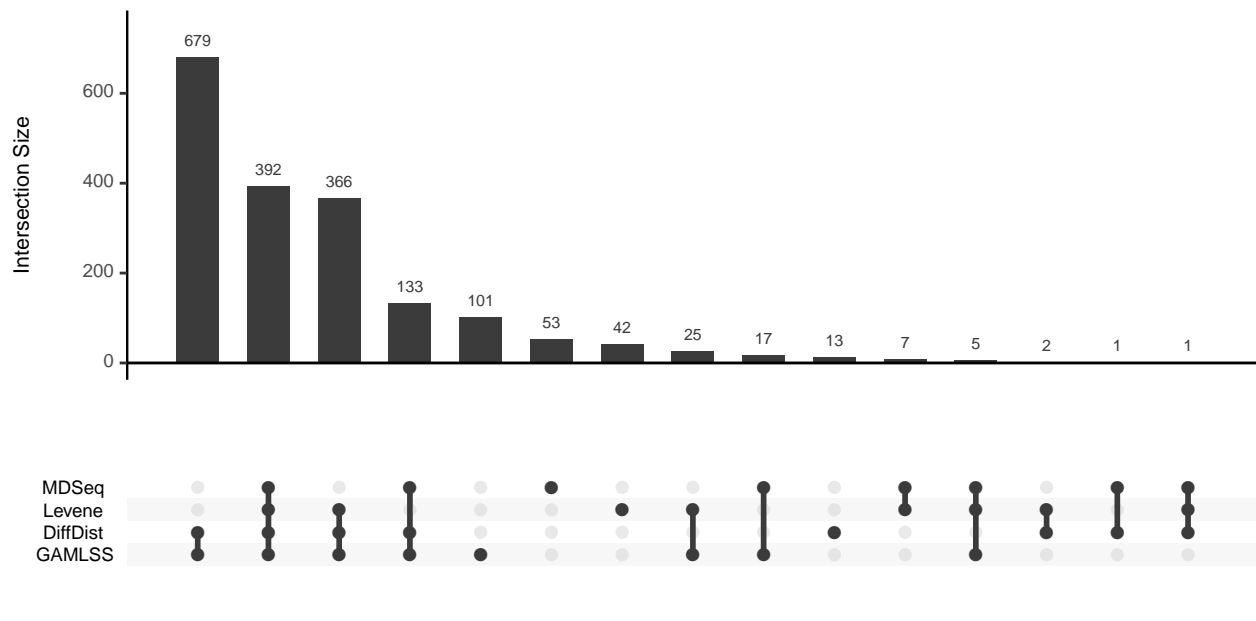

**B**

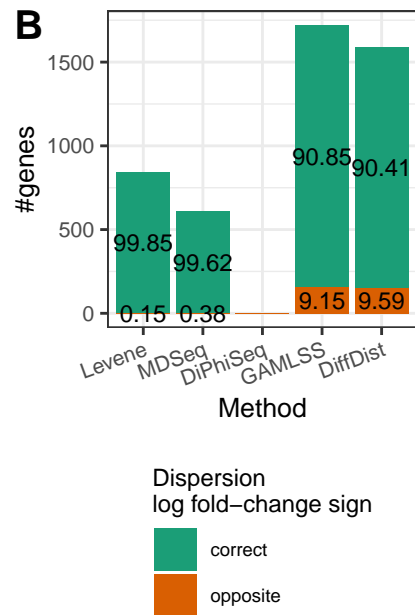

**C**

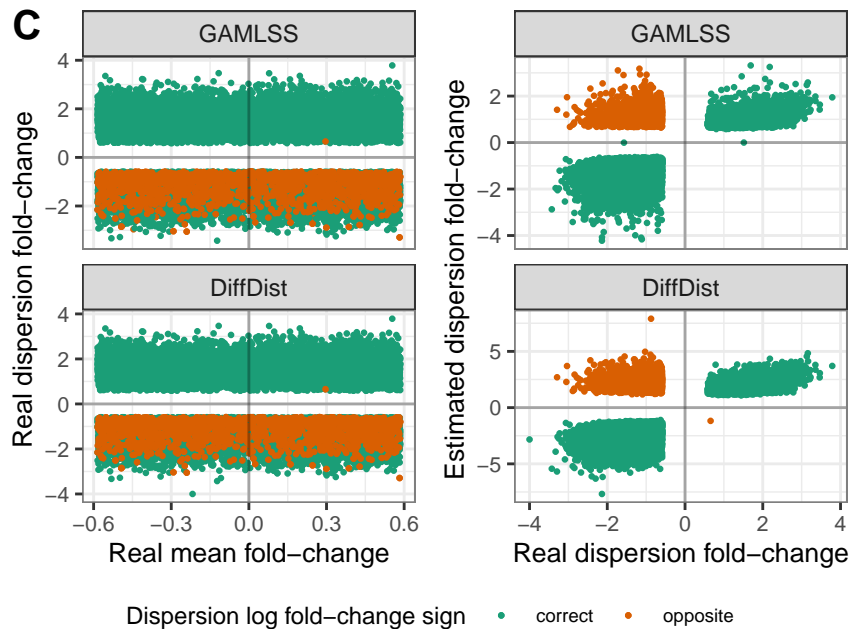

**D**

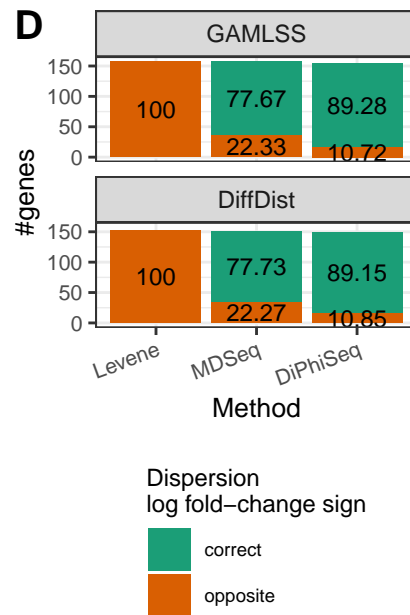

Sample size: 40

**A**

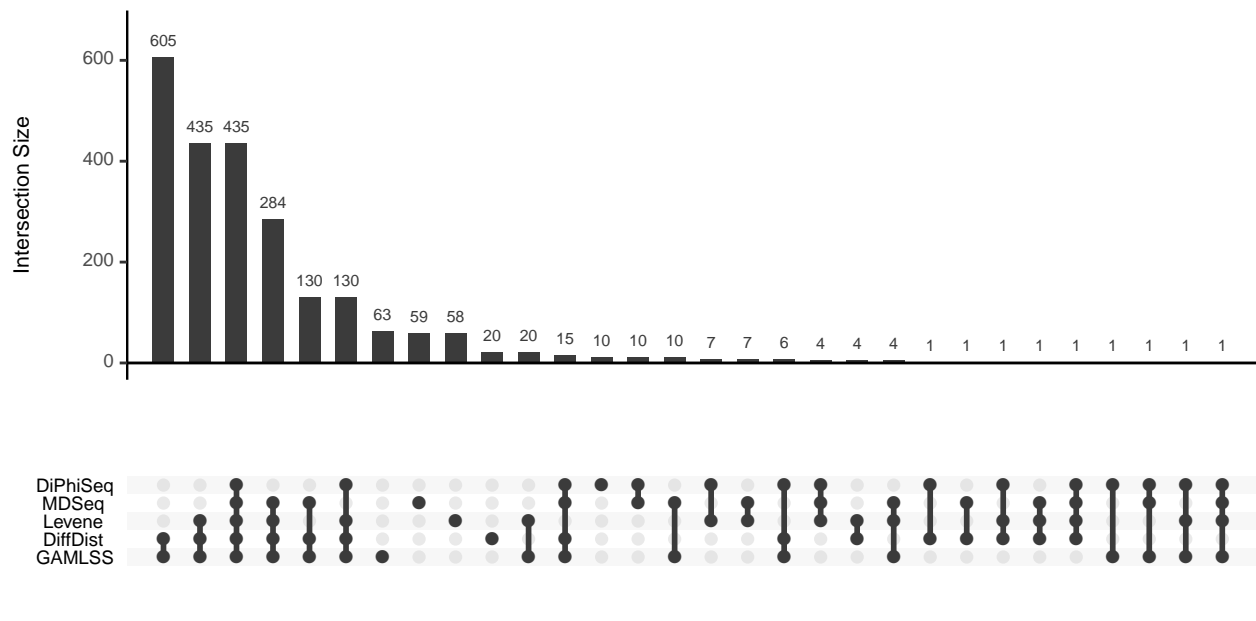

**B**

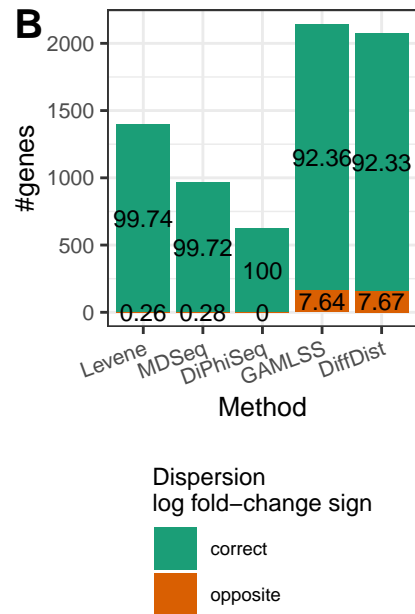

**C**

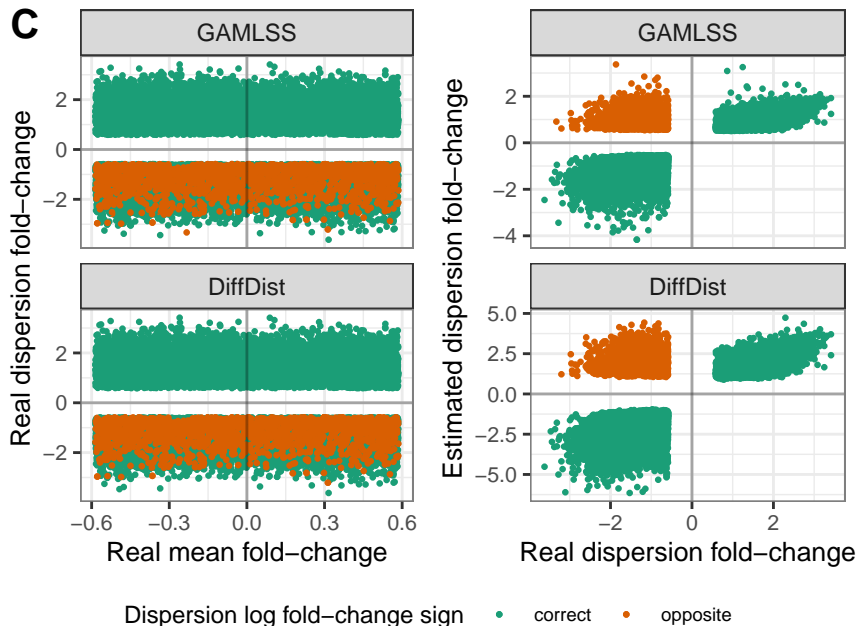

**D**

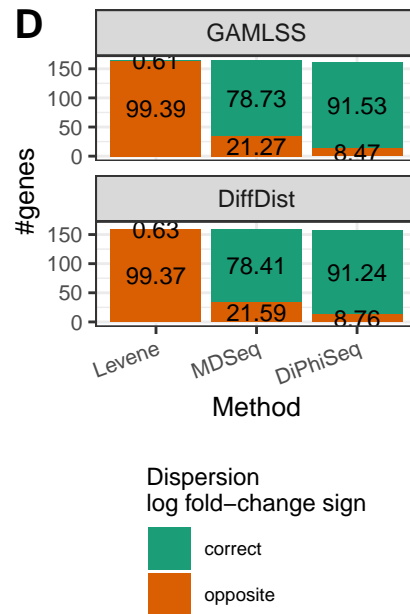

Sample size: 100

**A**

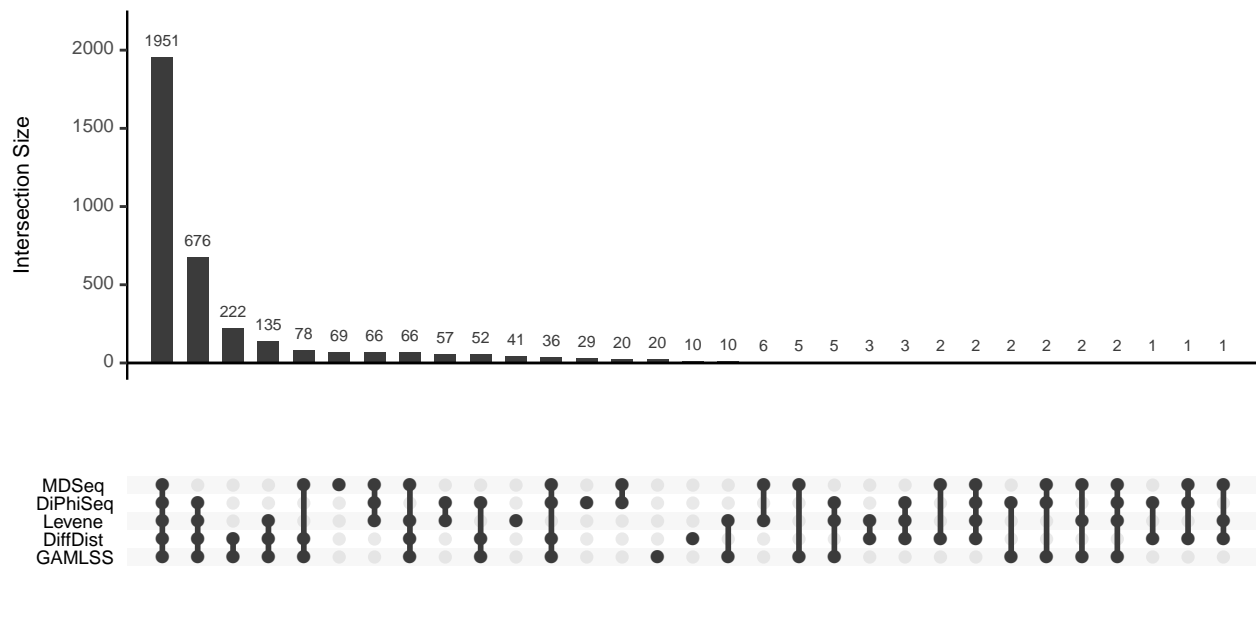

**B**

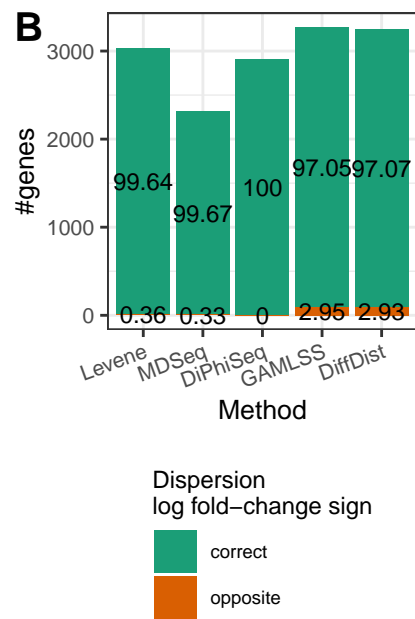

**C**

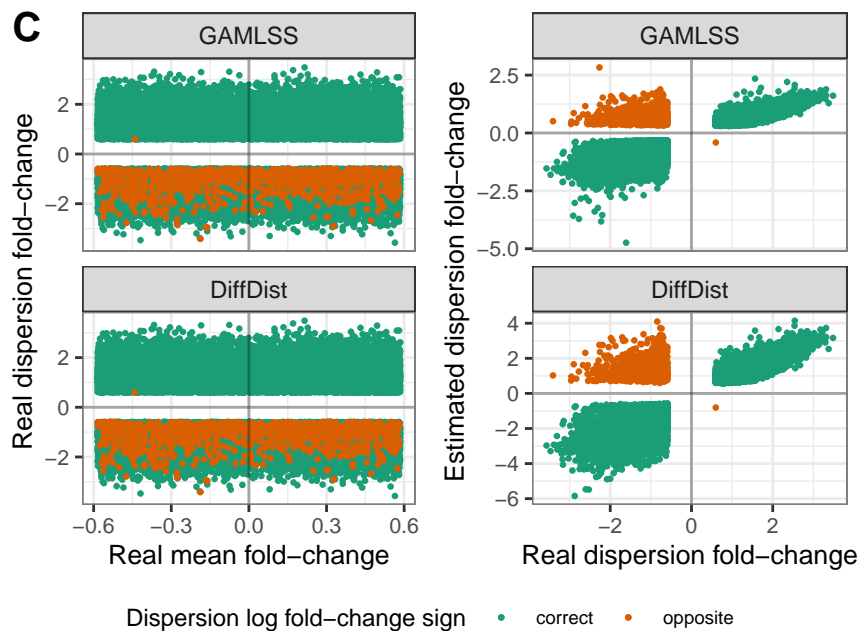

**D**

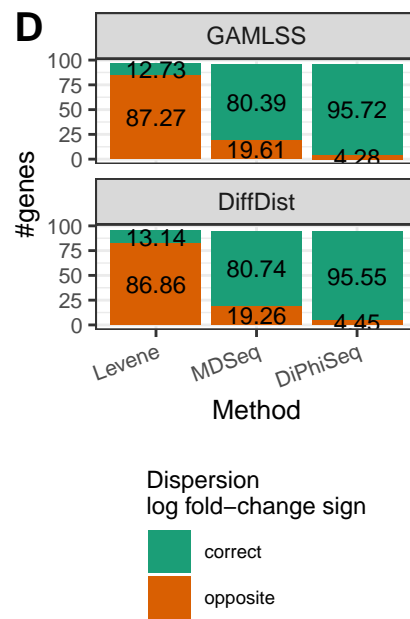

Supplement: S1 File — (A) Intersections of sets of differentially dispersed (DD) genes correctly identified by Levene’s test, MDSeq, DiPhiSeq, GAMLSS and DiffDist. (B): Correctness of dispersion log2-fold change sign of DD genes correctly identified by the different methods. (C) Real mean and dispersion log2-fold changes and estimated dispersion log2-fold changes of DD genes correctly identified by GAMLSS and DiffDist. (D) Correctness of dispersion log2-fold change signs according to Levene’s test, MDSeq and DiPhiSeq for DD genes correctly identified by GAMLSS and DiffDist with incorrect log2-fold change sign. Simulated datasets are composed of two populations of 20, 30, 40 or 100 samples and lowly differentially expressed genes have a mean fold change of expression between 1 and 1.5. All counts are averaged over 10 replicates of simulated datasets. (PDF) [file pcbi.1010342.s008.pdf]

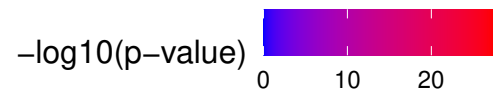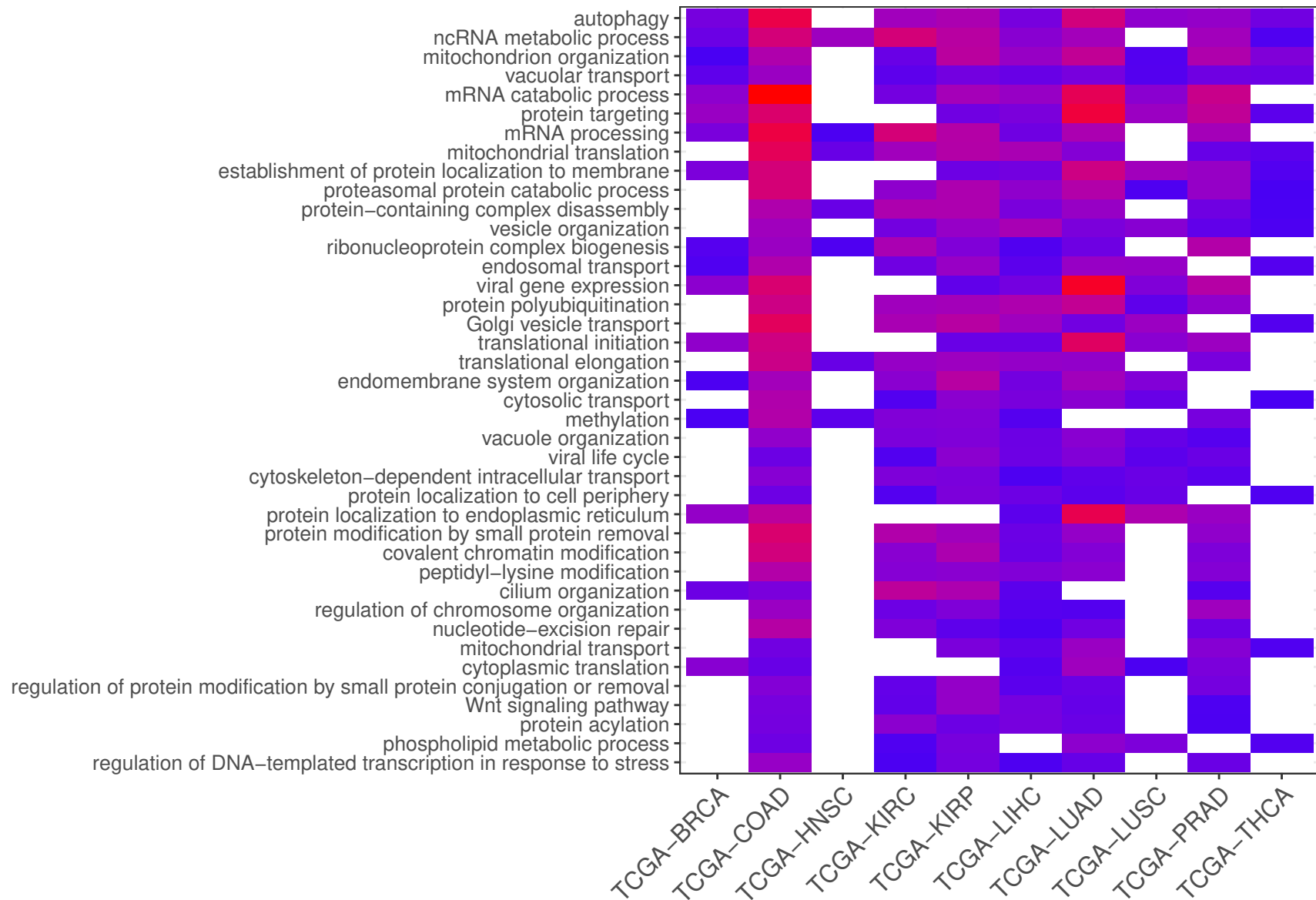

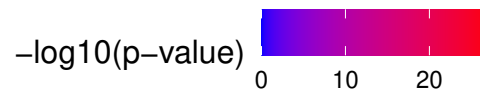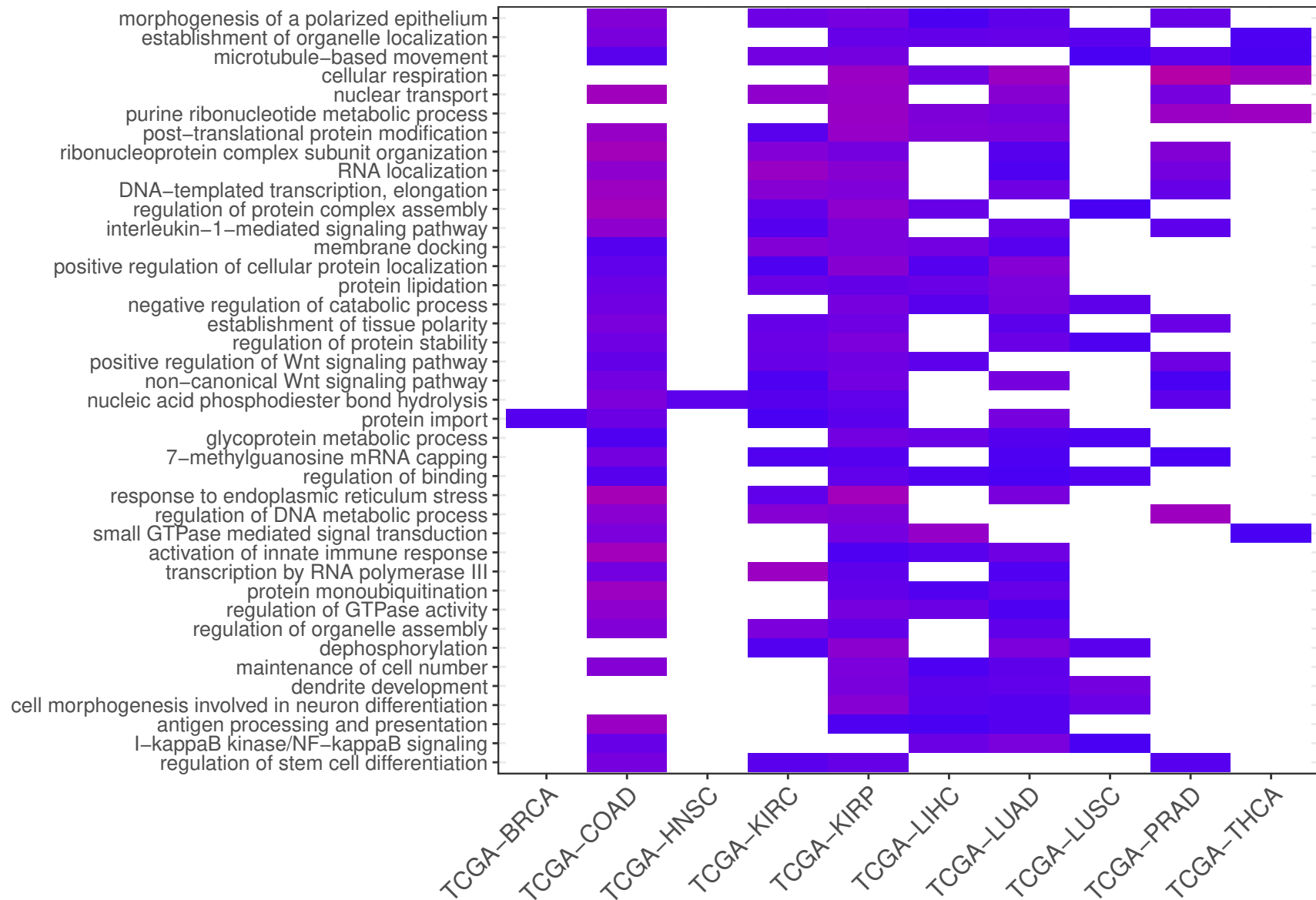

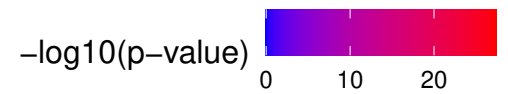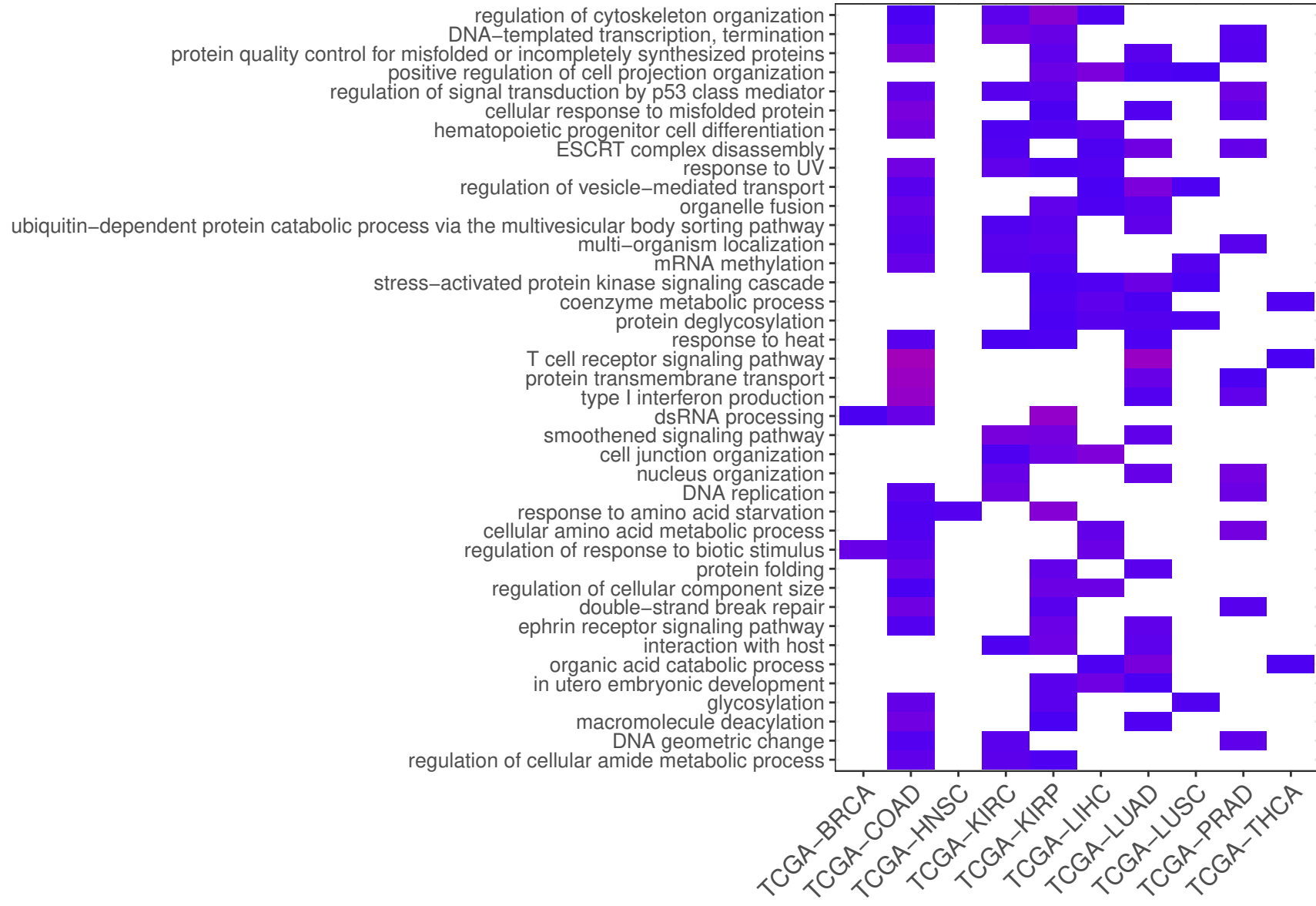

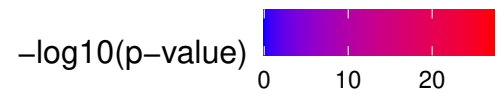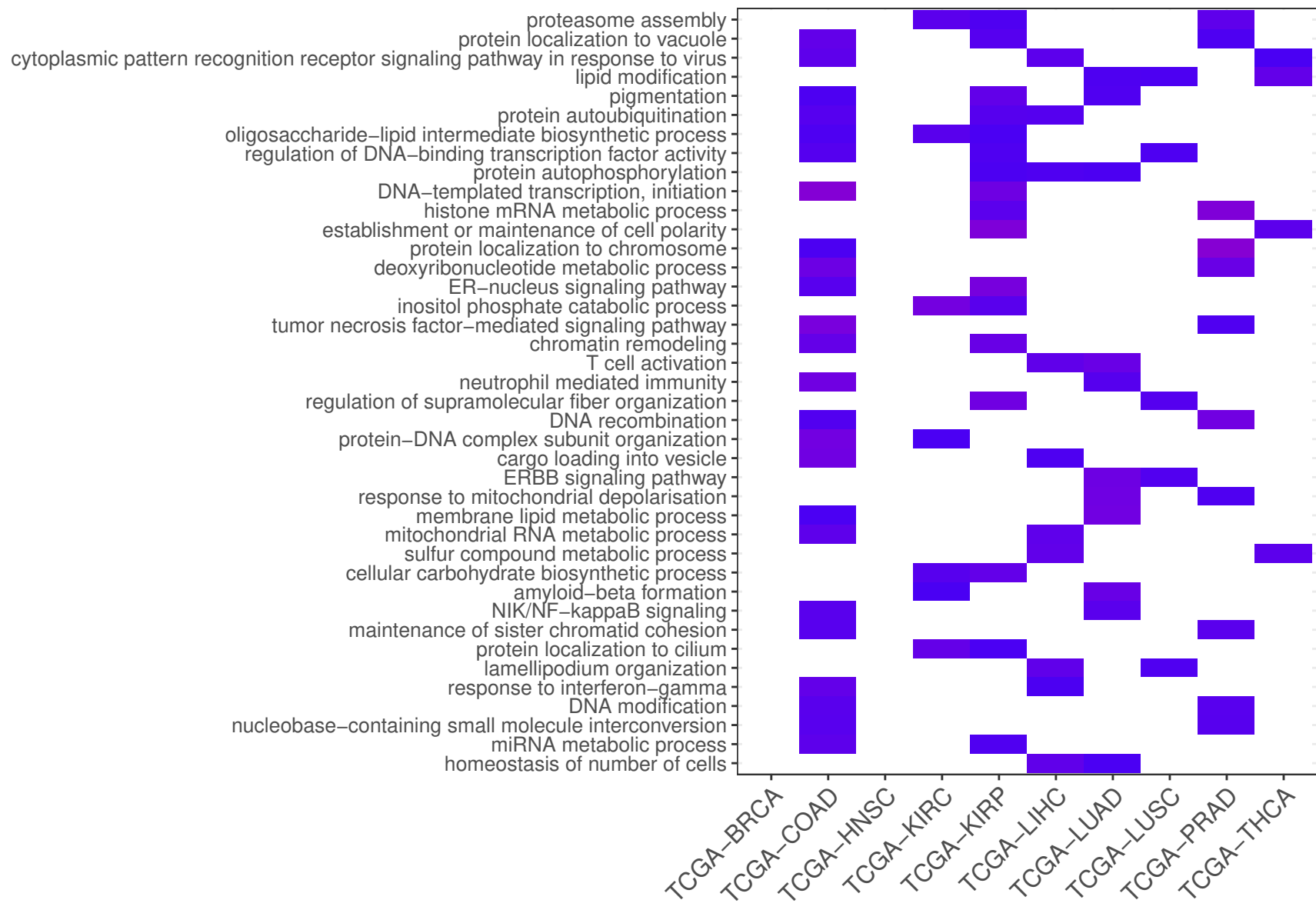

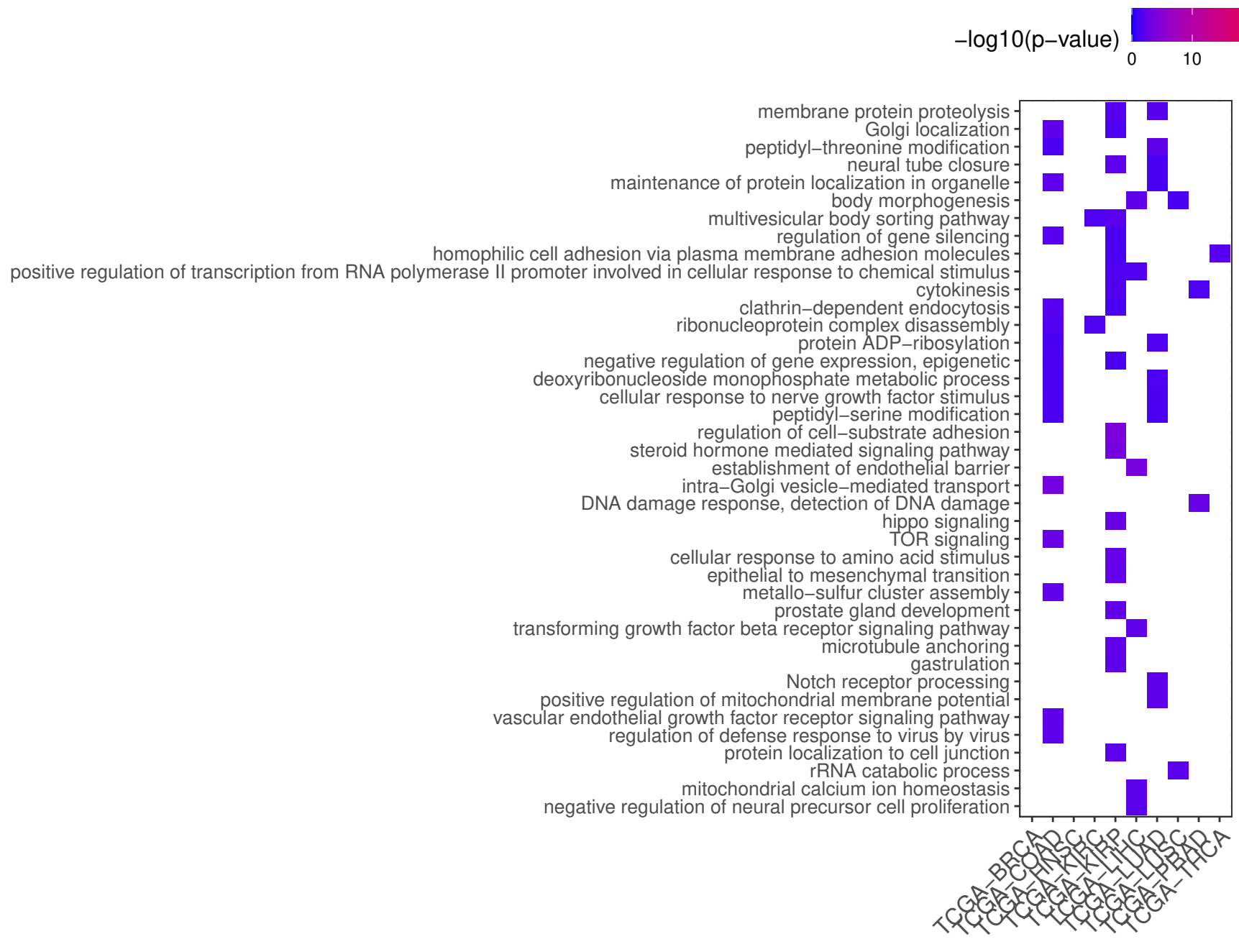

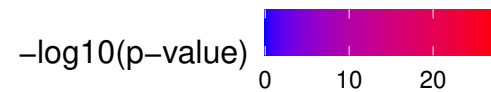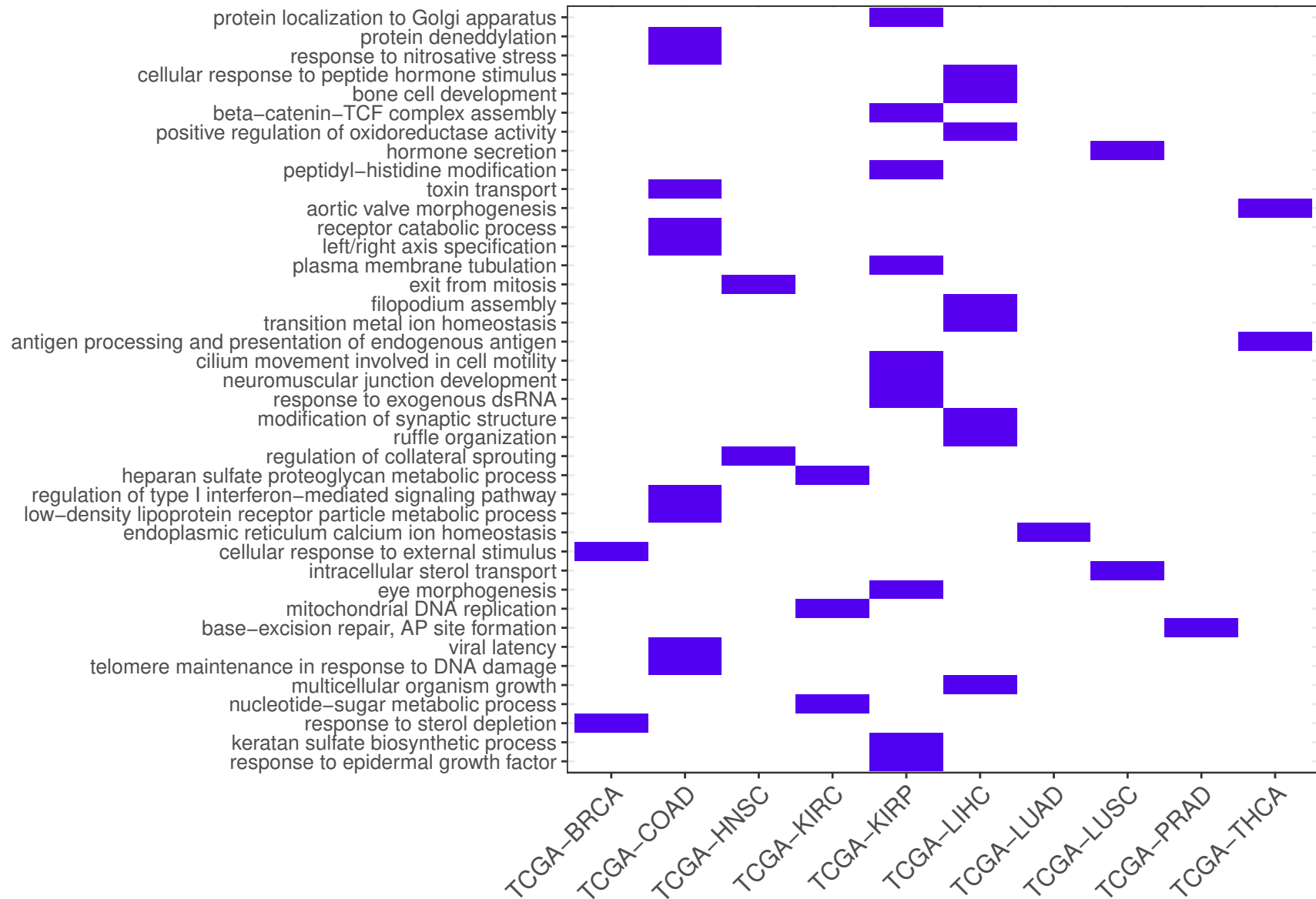

$-\log_{10}(\text{p-value})$

0 10 20

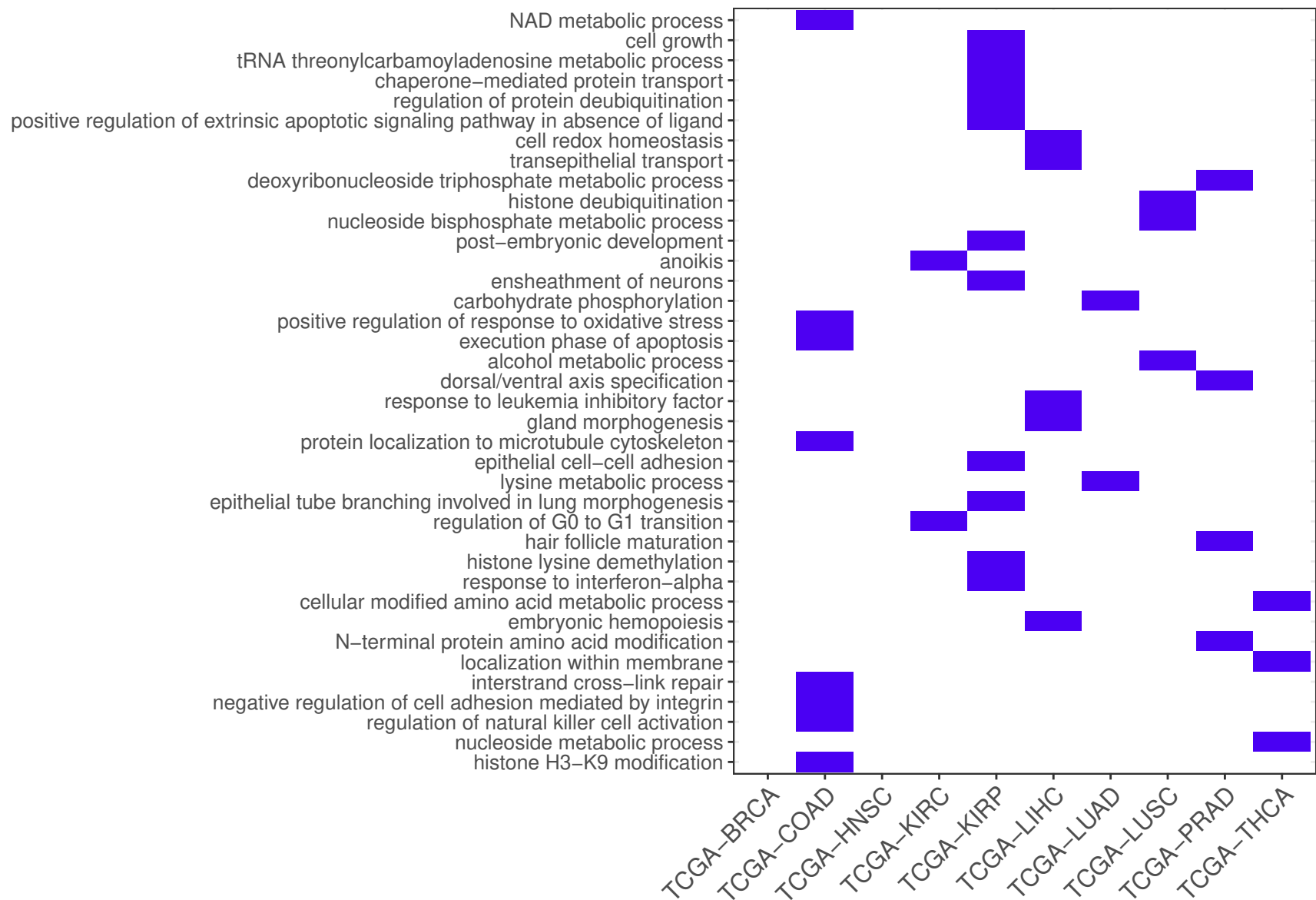

Supplement: S3 File — Full list of representative enriched Gene Ontology (GO) terms among overdispersed genes in tumors (DD+) among non-differentially expressed (non-DE) genes, ordered first by the number of datasets for which they are enriched (decreasing order) and second by the mean p-values of enrichment across all datasets (increasing order). Non-DE genes were identified using MDSeq, and DD+ genes were identified among non-differentially expressed genes by at least one of the evaluated methods, i.e. Levene’s test, MDSeq, DiPhiSeq, GAMLSS and DiffDist. (PDF) [file pcbi.1010342.s010.pdf]

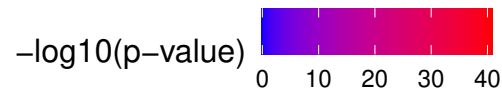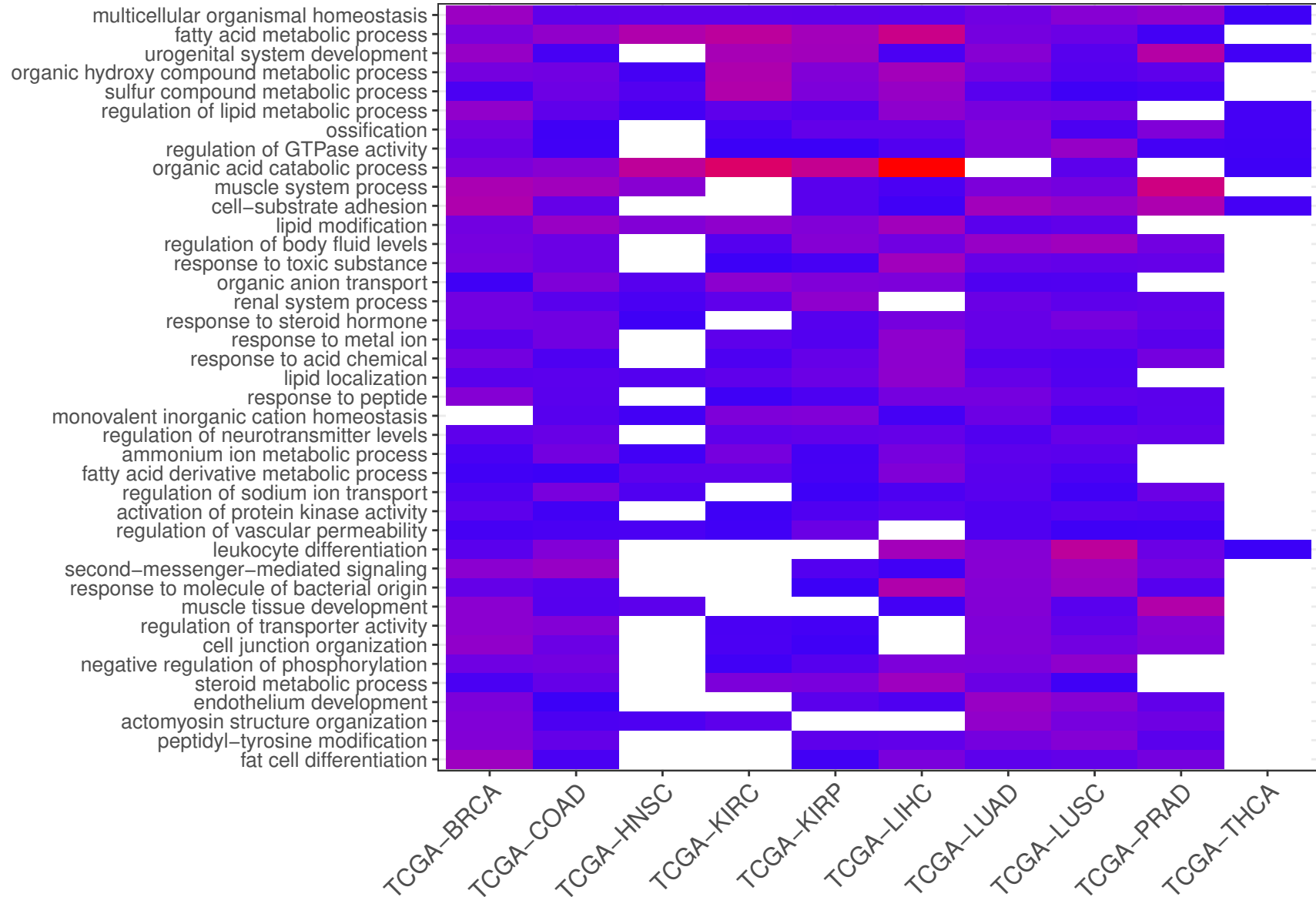

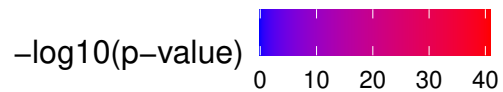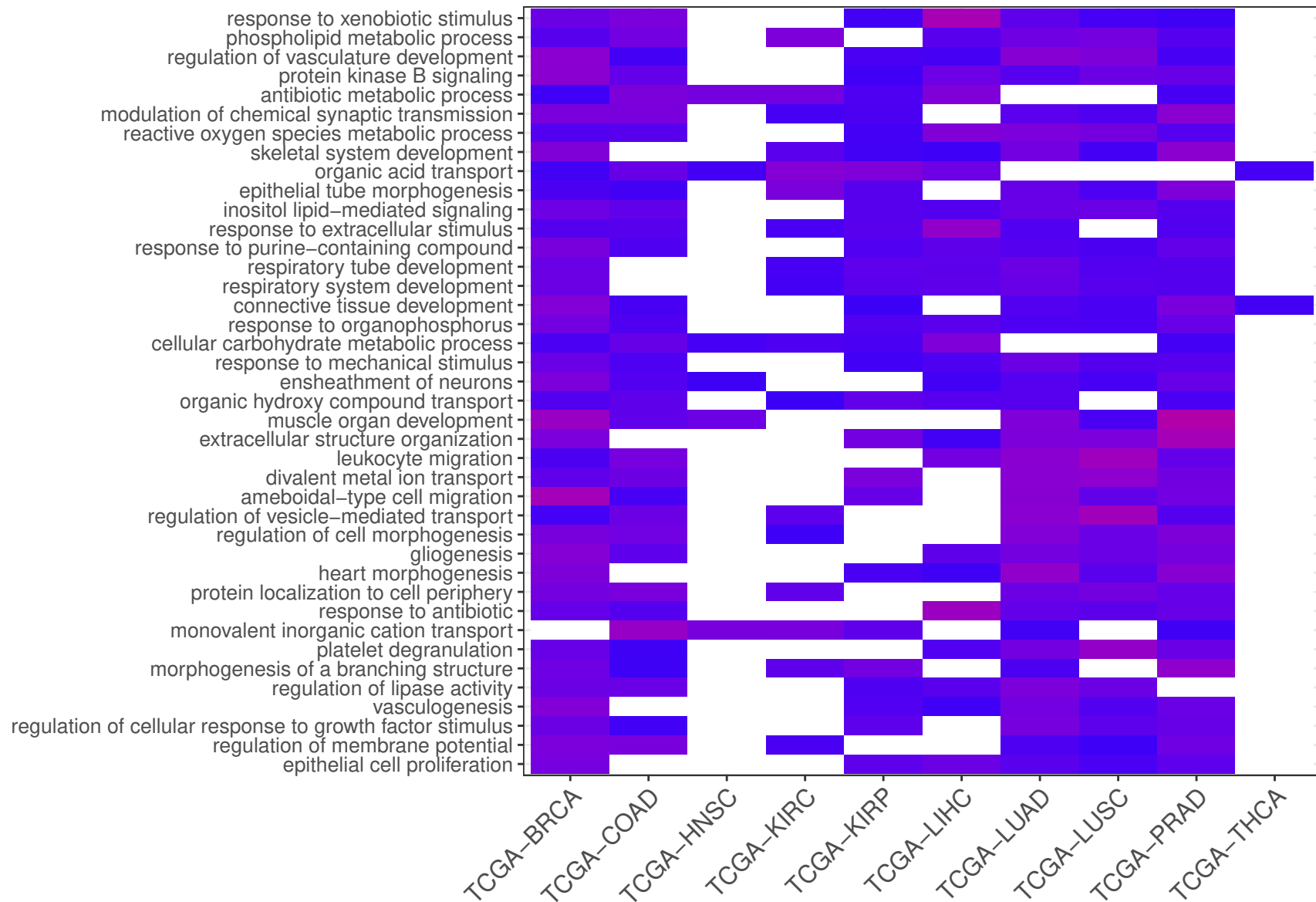

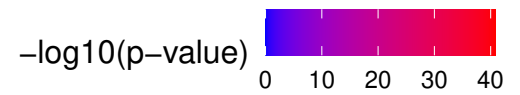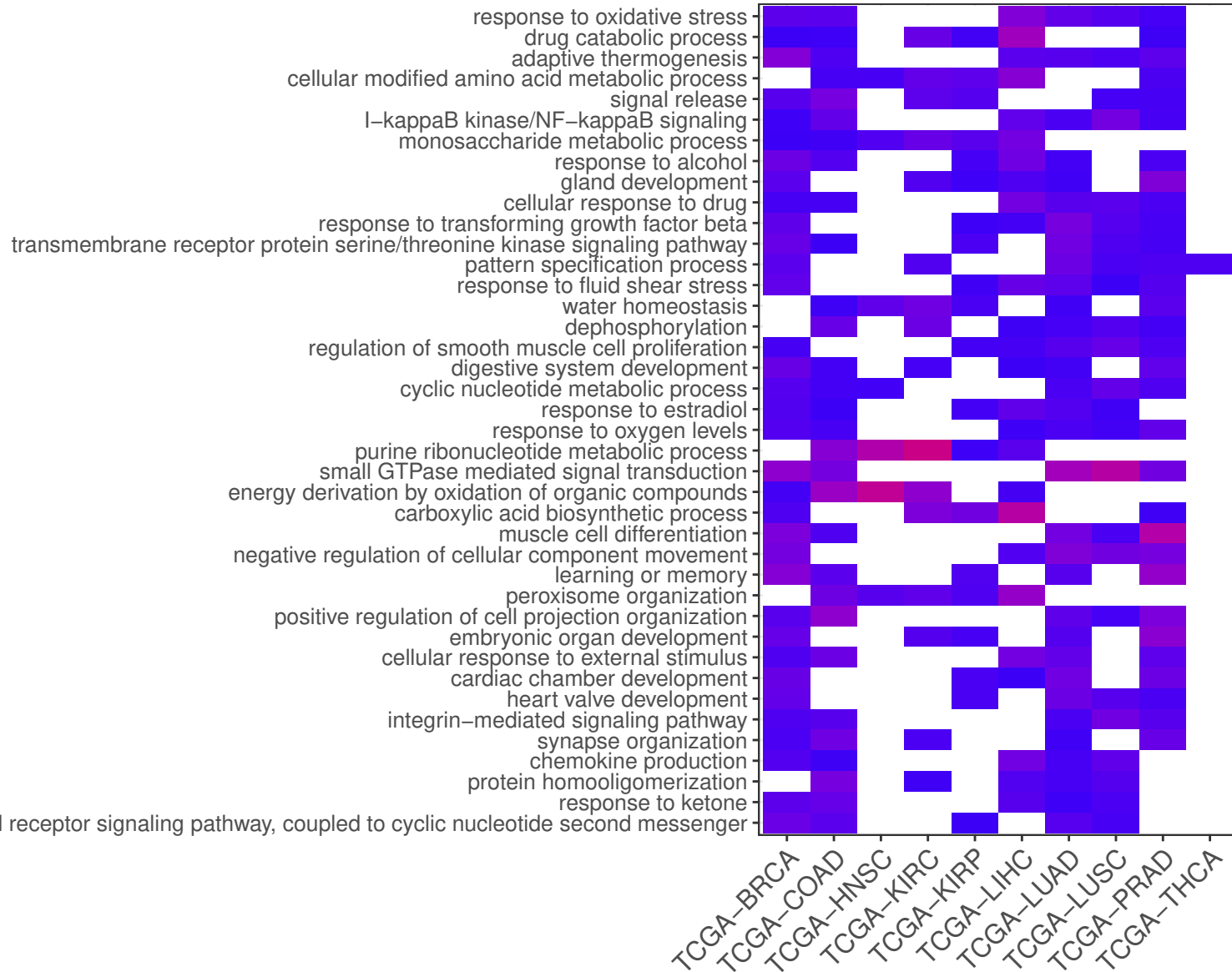

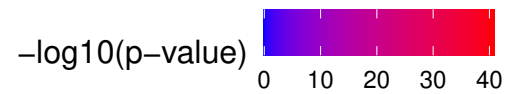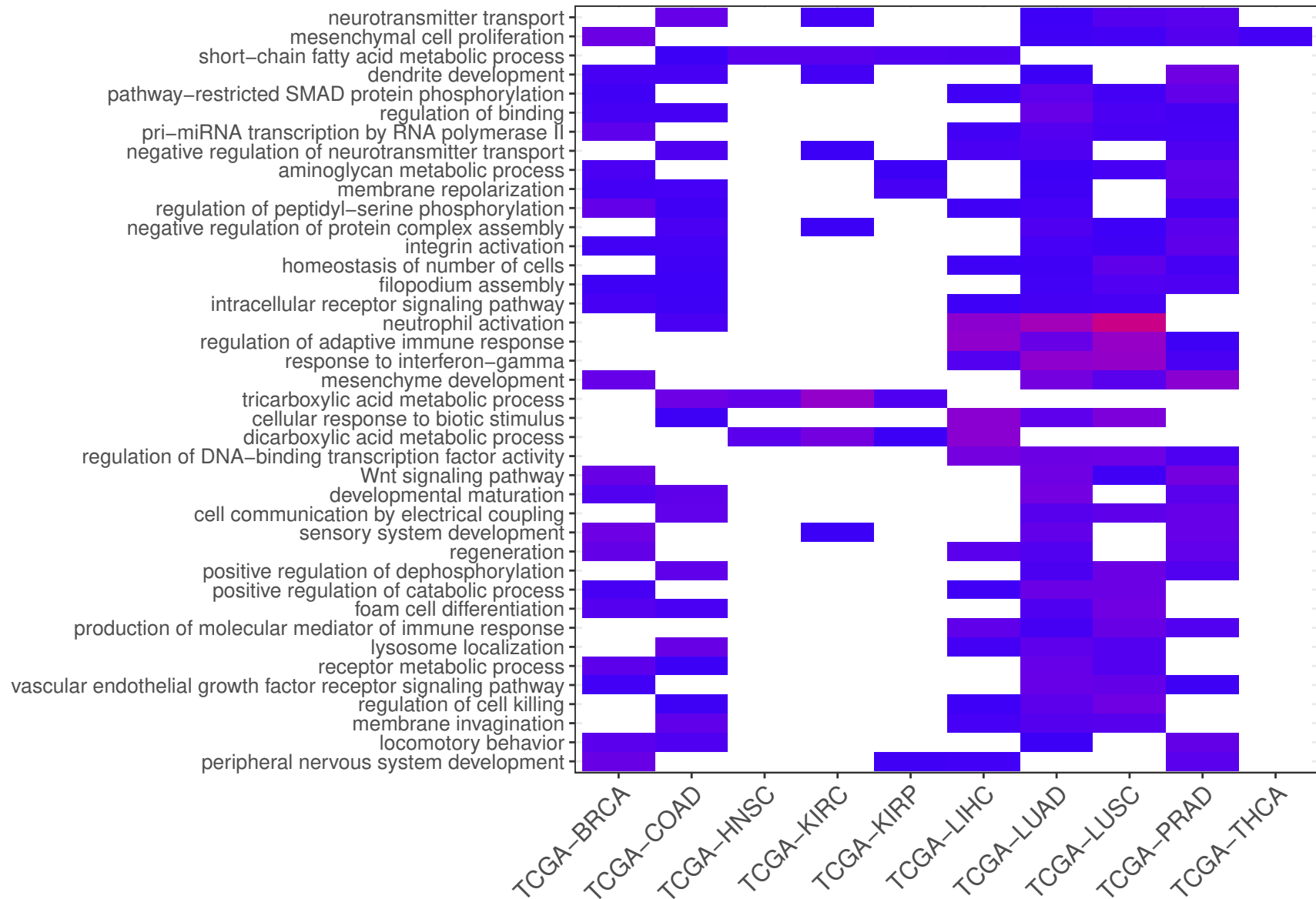

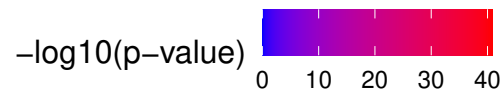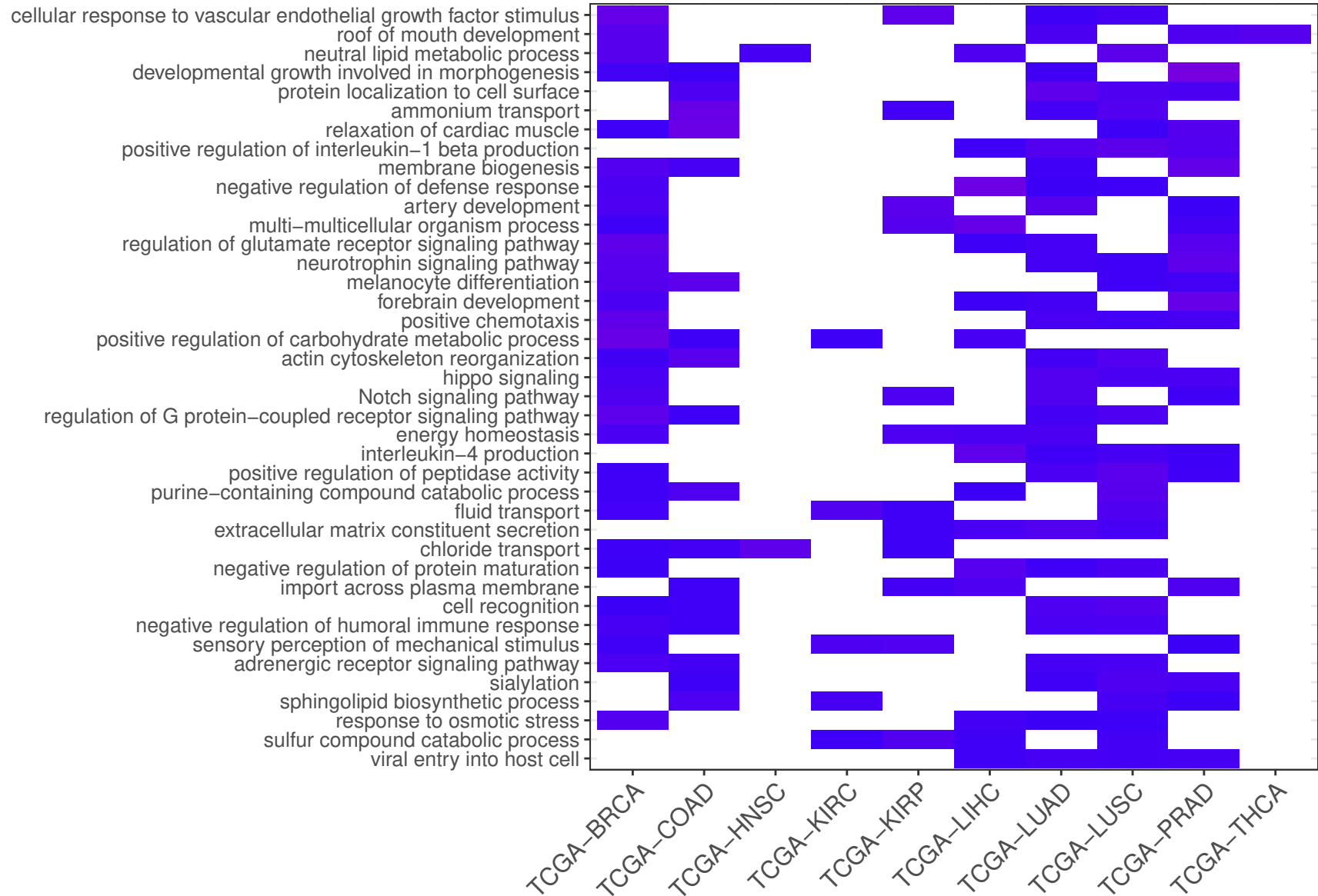

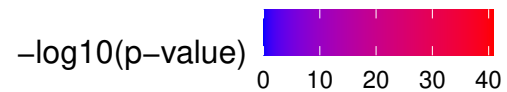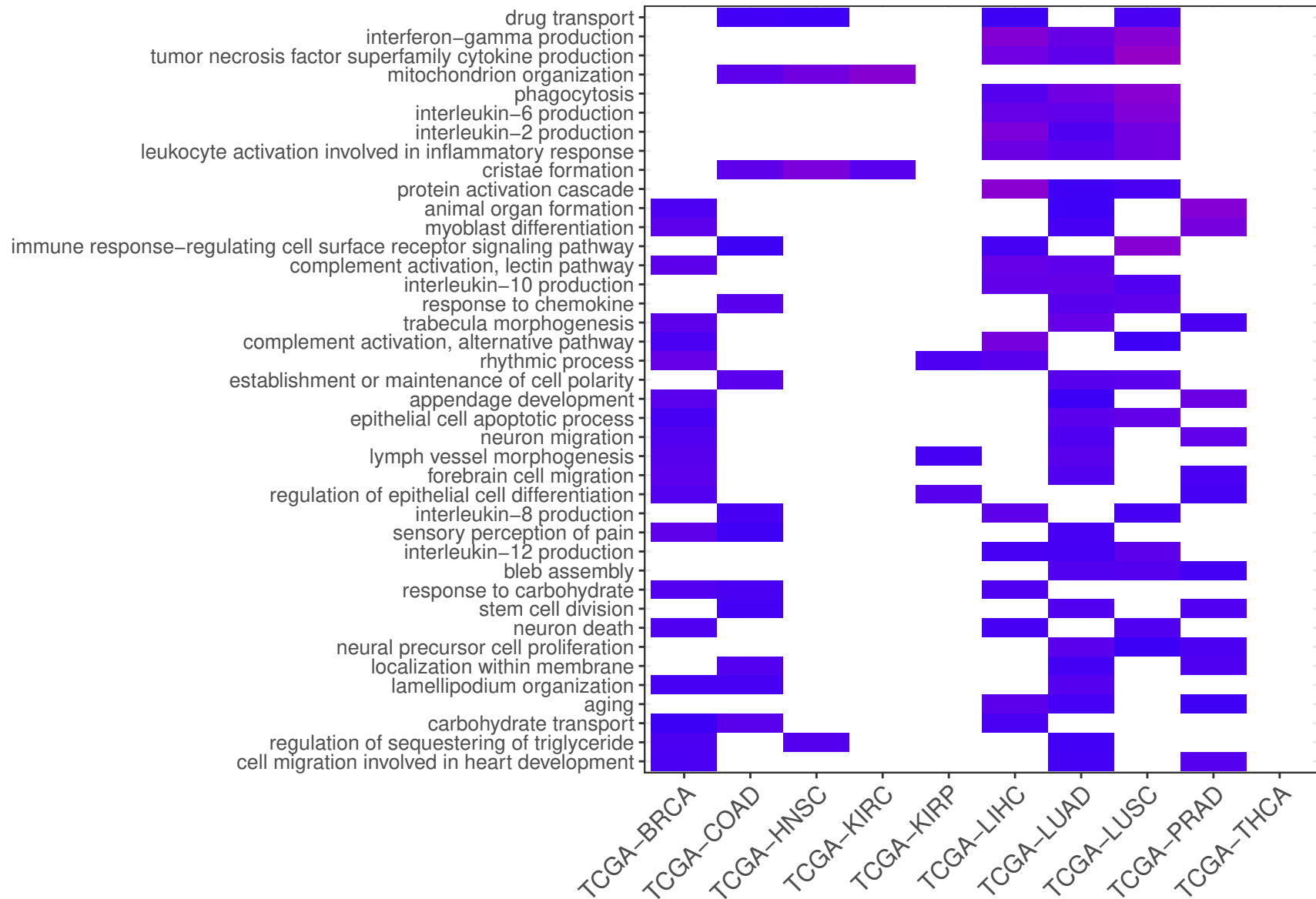

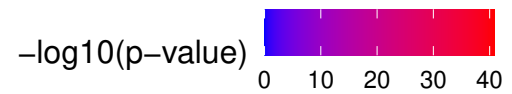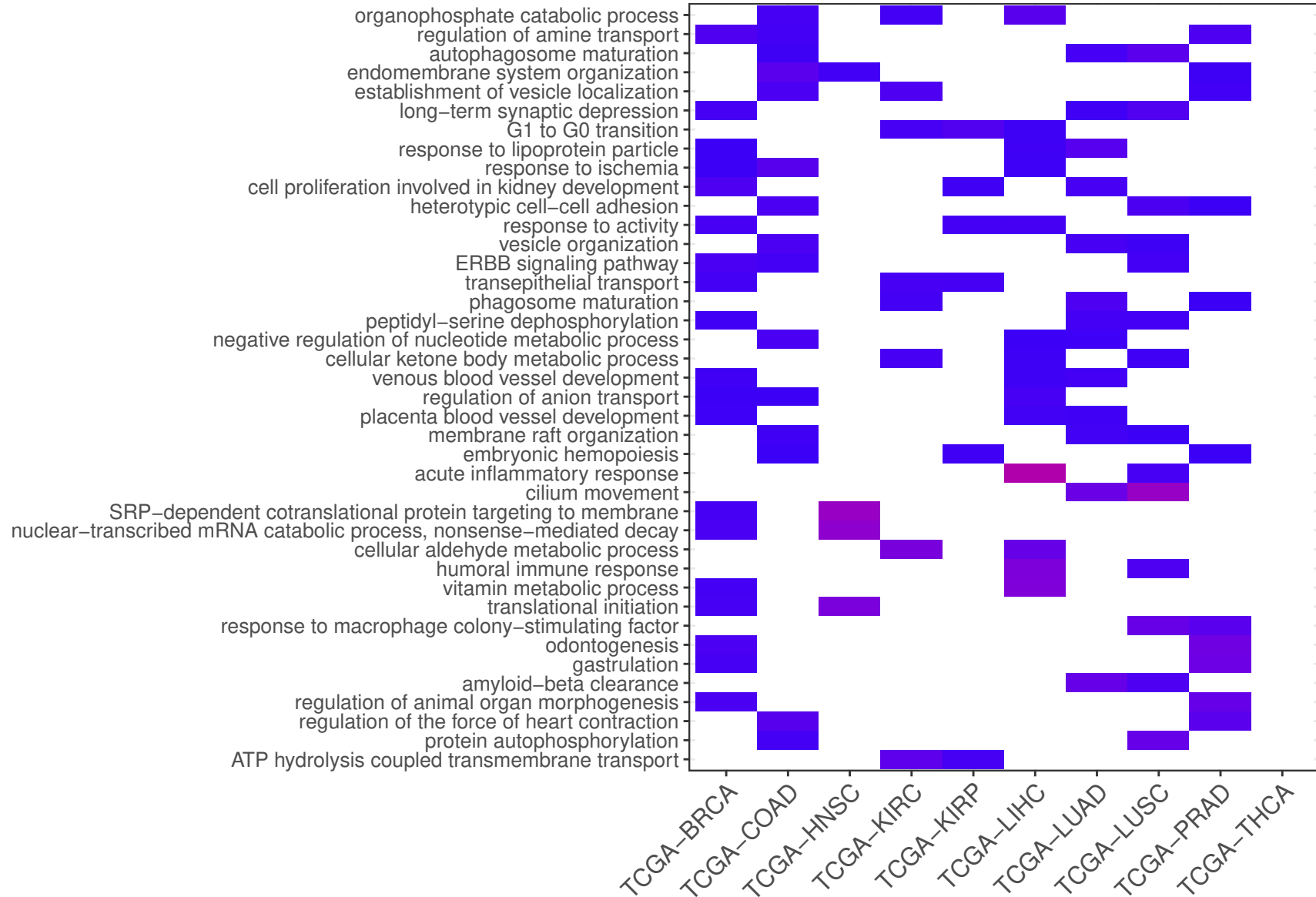

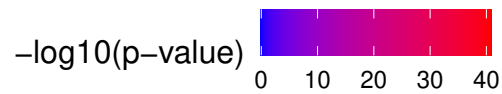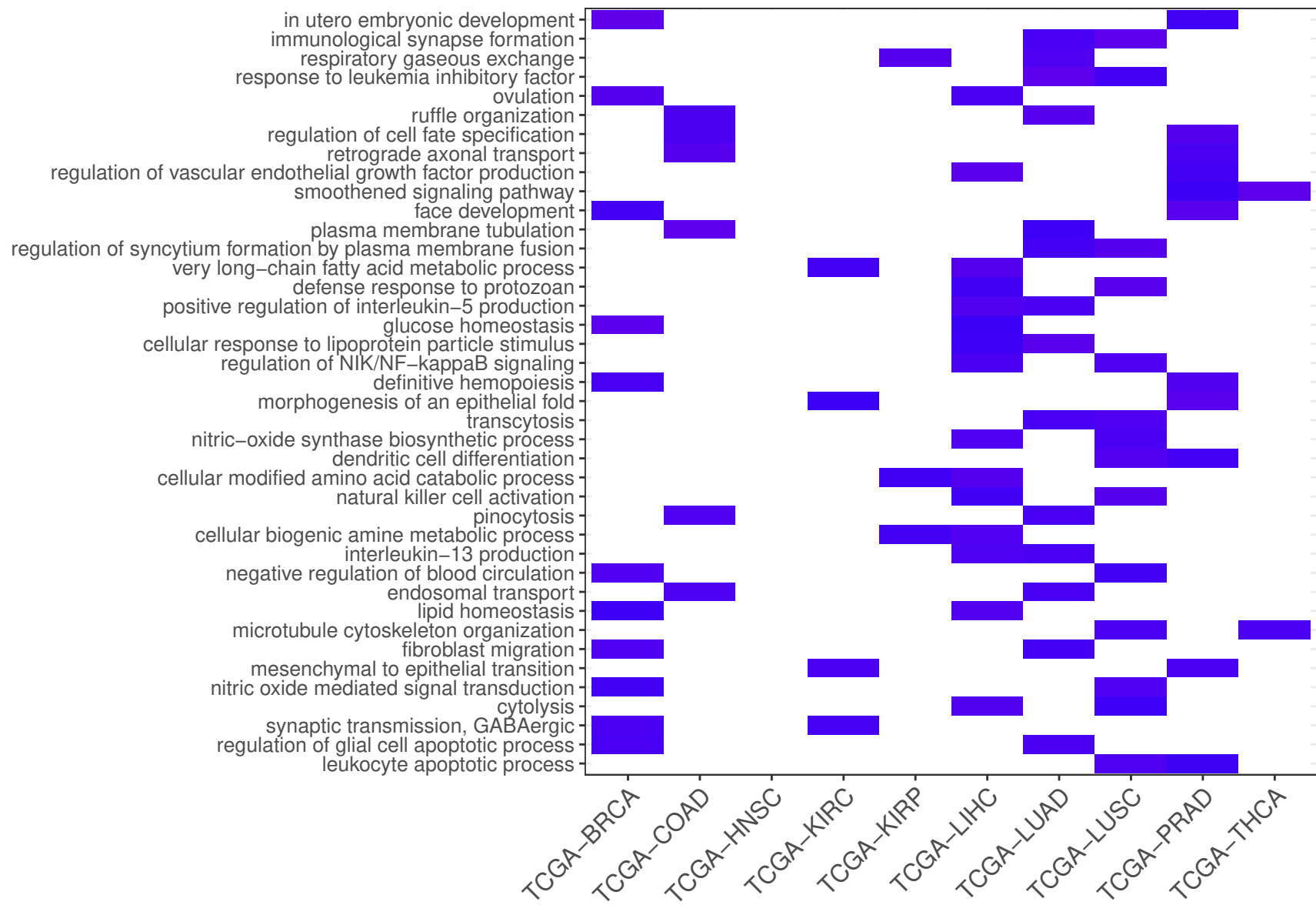

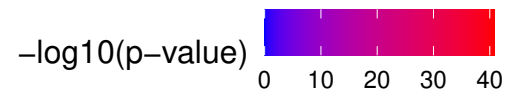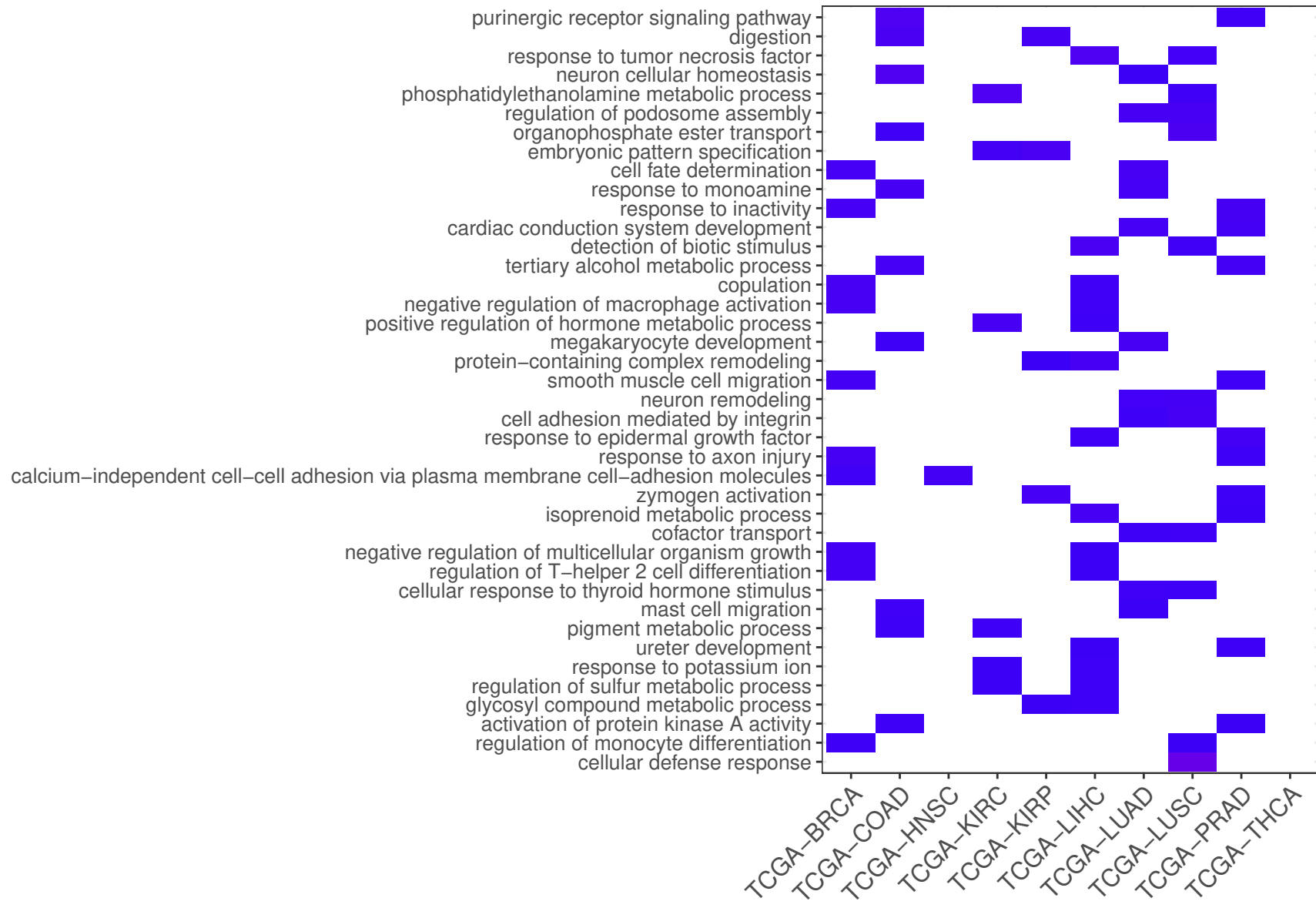

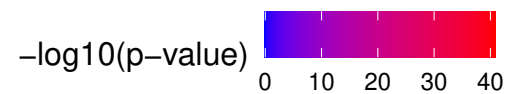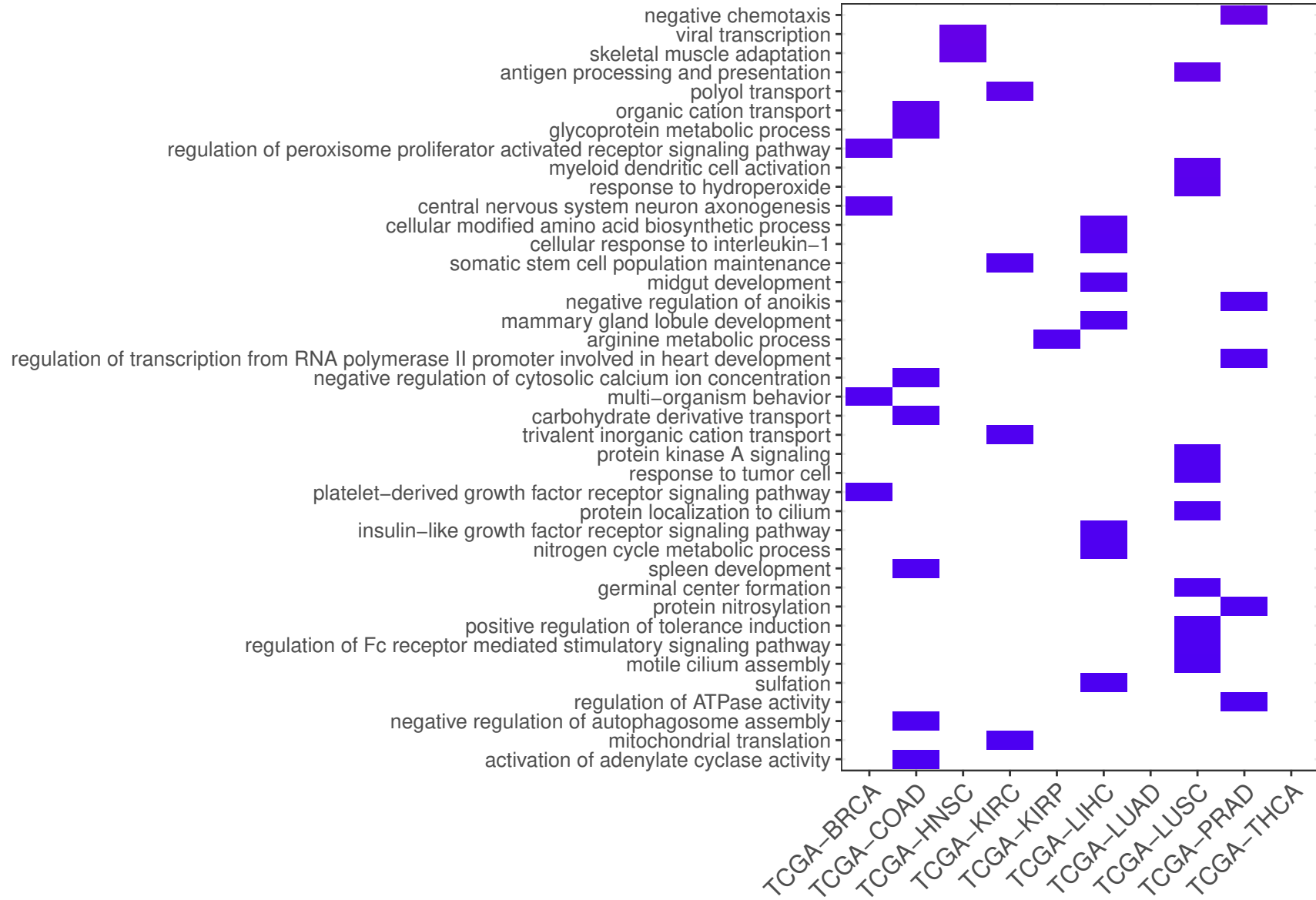

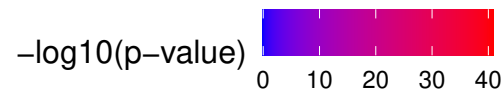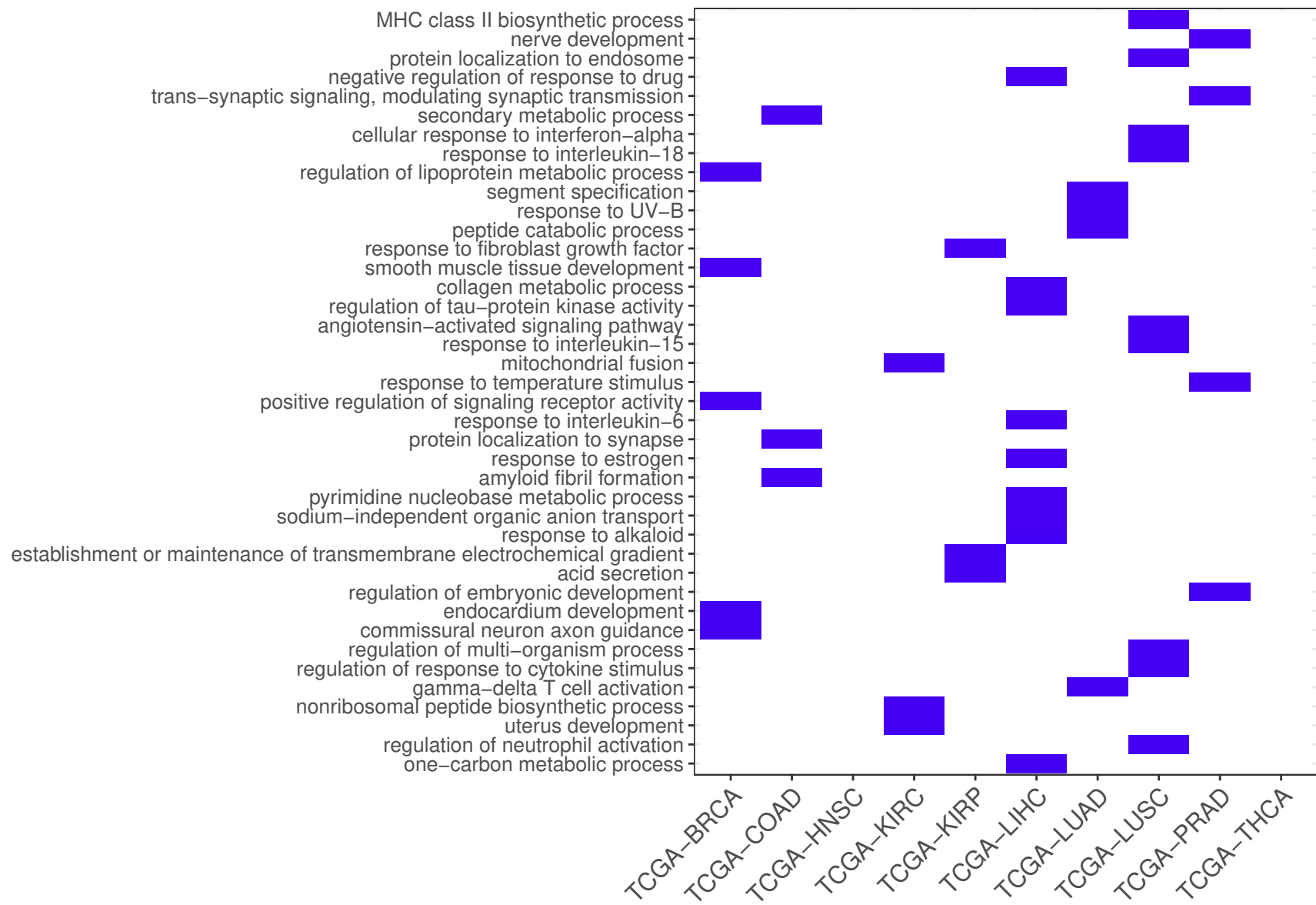

$-\log_{10}(\text{p-value})$

0 10 20 30 40

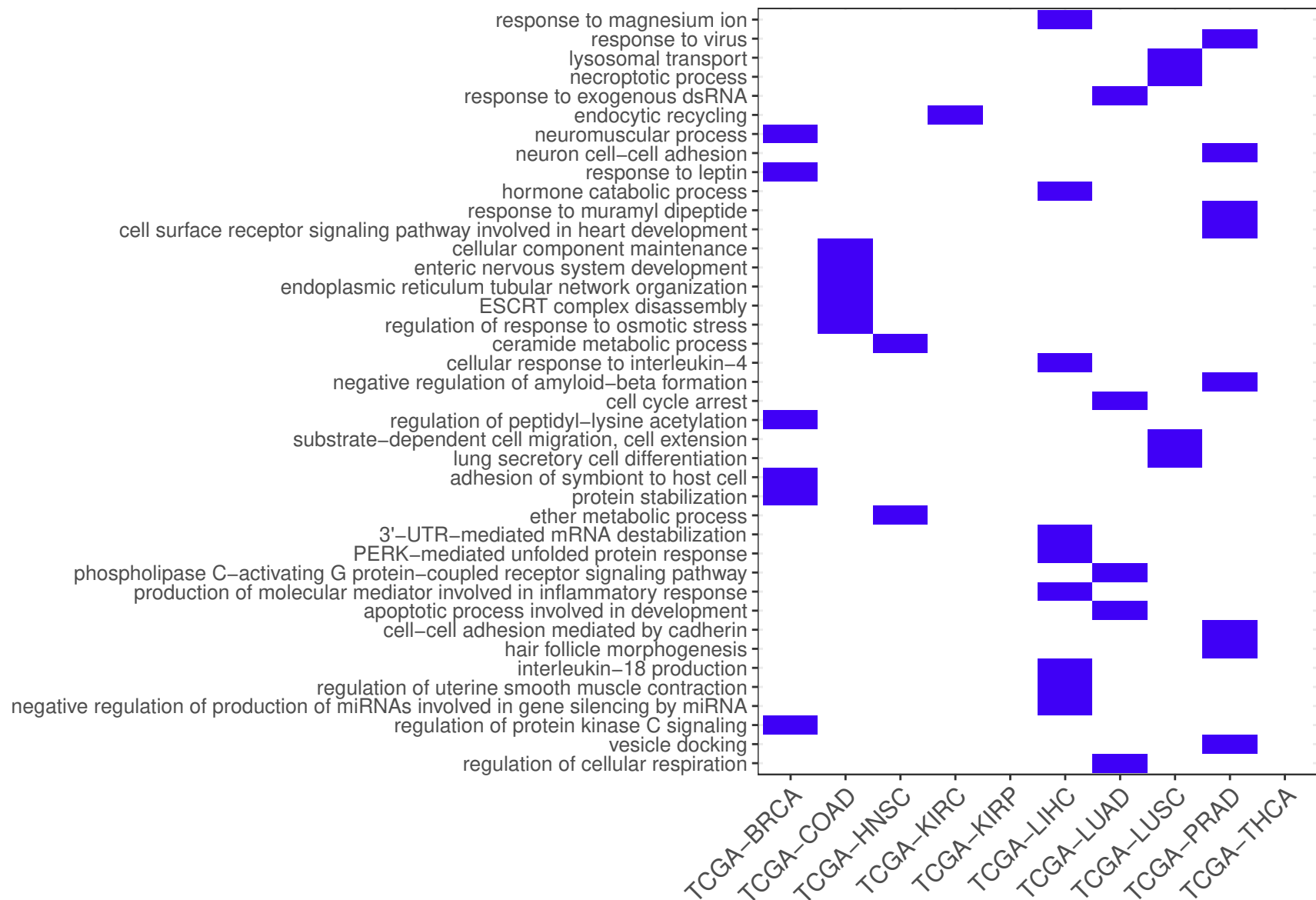

$-\log_{10}(\text{p-value})$

0 10 20 30 40

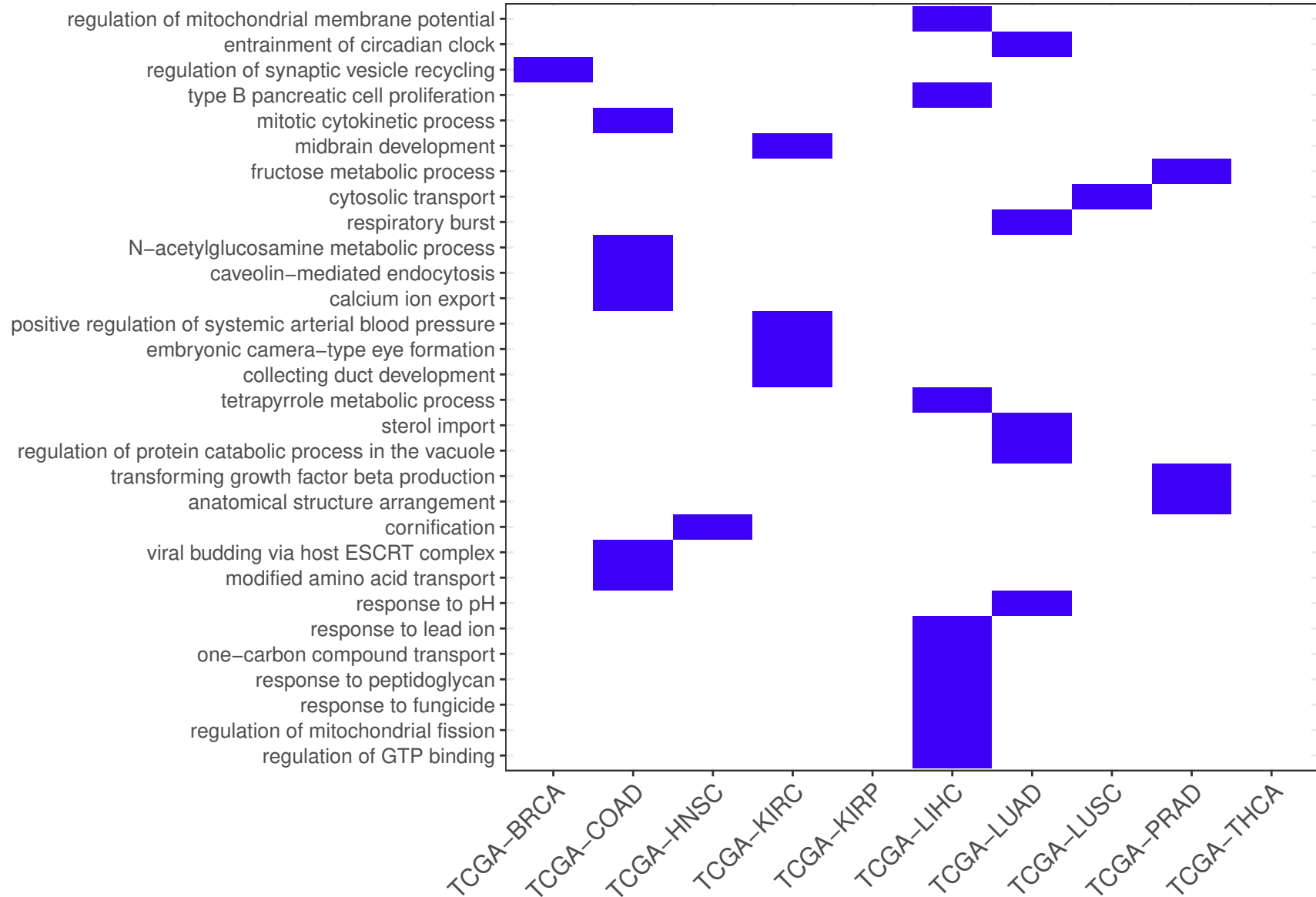

Supplement: S5 File — Full list of representative enriched Gene Ontology (GO) terms among downregulated genes in tumors, ordered first by the number of datasets for which they are enriched (decreasing order) and second by the mean p-values of enrichment across all datasets (increasing order). Downregulated genes were identified by MDSeq using a mean fold change threshold of 1. (PDF) [file pcbi.1010342.s012.pdf]
