# Supplementary material for: Detection of genes with differential expression dispersion unravels the role of autophagy in cancer progression
Source: PLoS Comput Biol. 2023 Mar 9;19(3):e1010342. doi: 10.1371/journal.pcbi.1010342 (PMC9997931; doi:10.1371/journal.pcbi.1010342)
Supplement: S2 File — Intersections of sets of overdispersed genes in tumors (DD+) identified by Levene’s test, MDSeq, DiPhiSeq, GAMLSS and DiffDist among non-differentially expressed genes for the following TCGA datasets: breast invasive carcinoma (TCGA-BRCA), colon adenocarcinoma (TCGA-COAD), head and neck squamous cell carcinoma (TCGA-HNSC), liver hepatocellular carcinoma (TCGA-LIHC), lung adenocarcinoma (TCGA-LUAD), lung squamous cell carcinoma (TCGA-LUSC), prostate adenocarcinoma (TCGA-PRAD), and thyroid carcinoma (TCGA-THCA). Non-differentially expressed genes were identified by MDSeq. (PDF) [file pcbi.1010342.s009.pdf]

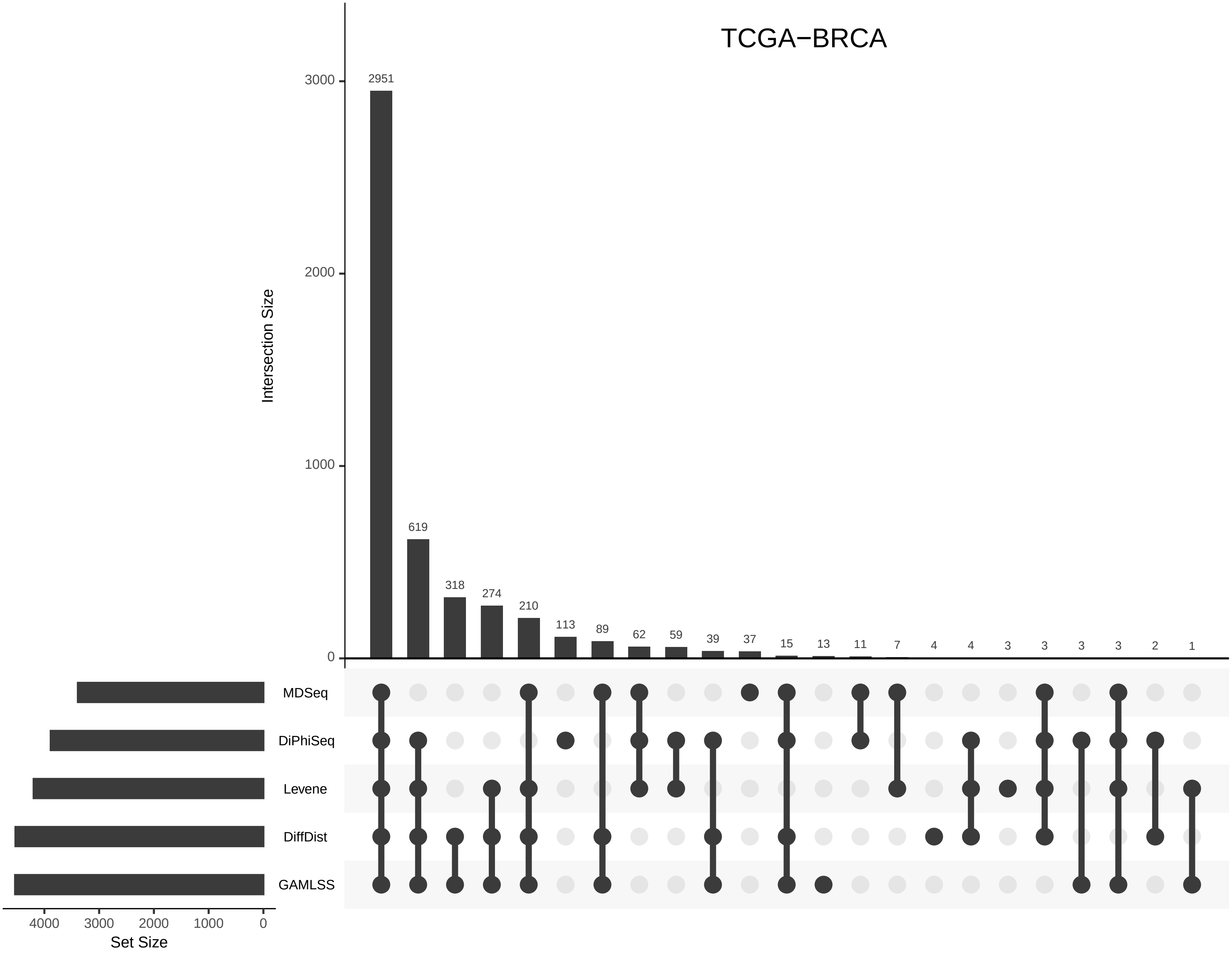
TCGA−BRCA
Intersection Size
3000
2000
1000
0
2951
619
318
274
210
113
89
62
59
39
37
15
13
11
7
4
4
3
3
3
3
2
1
MDSeq
DiPhiSeq
Levene
DiffDist
GAMLSS
4000
3000
2000
1000
0
Set Size

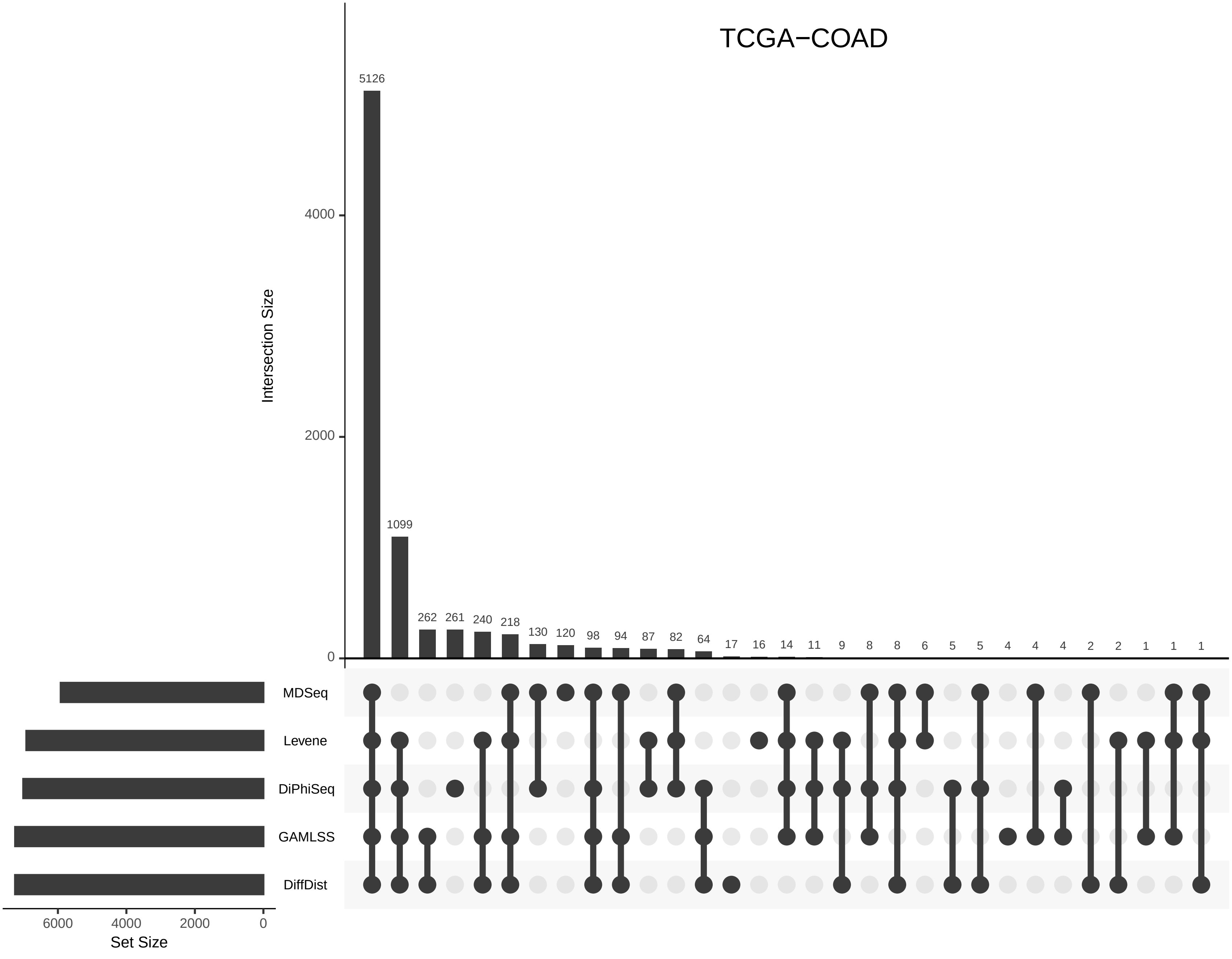

TCGA−COAD
Intersection Size
4000
2000
0
5126
1099
262
261
240
218
130
120
98
94
87
82
64
17
16
14
11
9
8
8
6
5
5
4
4
4
2
2
1
1
1
MDSeq
Levene
DiPhiSeq
GAMLSS
DiffDist
6000
4000
2000
0
Set Size

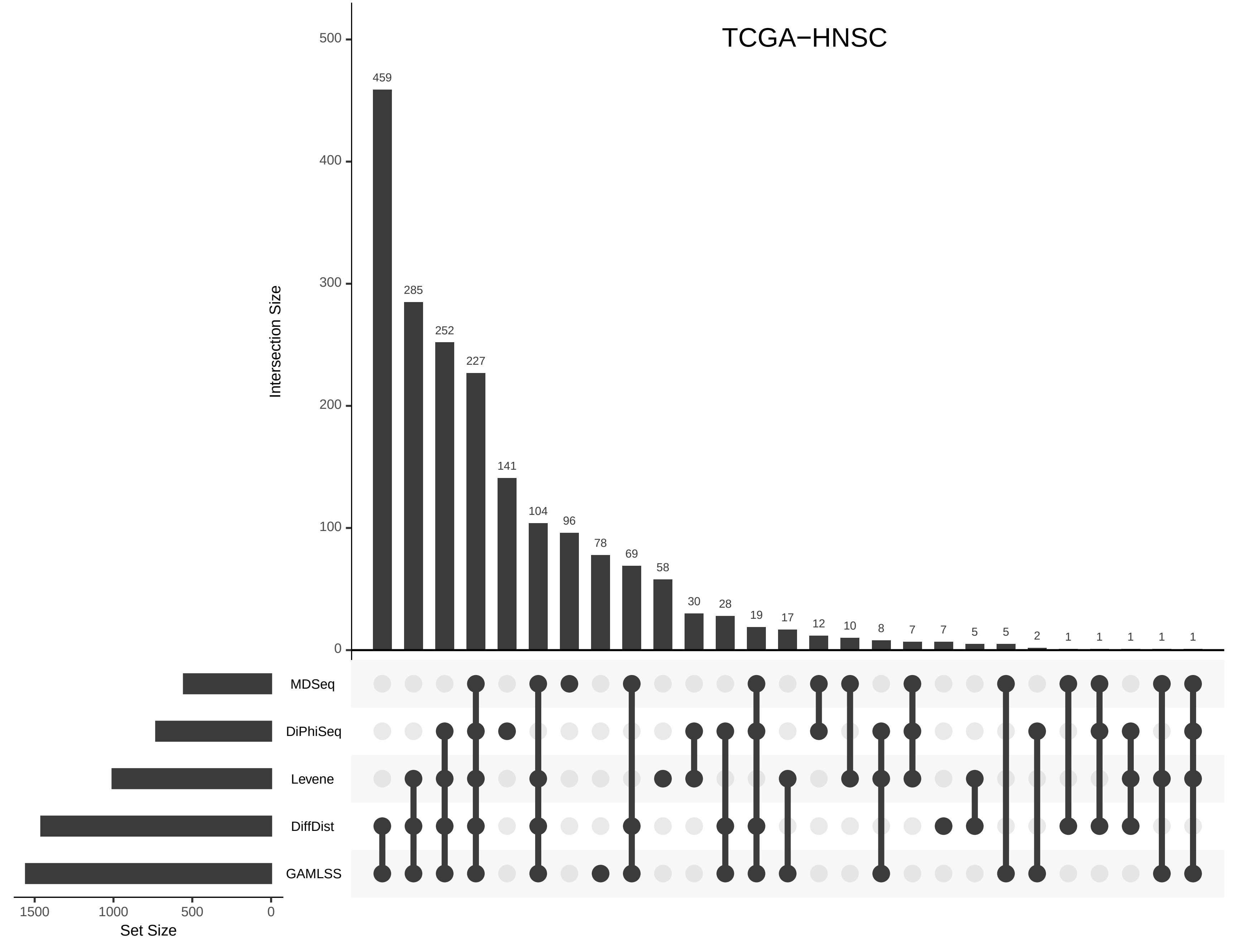

TCGA−HNSC
Intersection Size
0
100
200
300
400
500
459
285
252
227
141
104
96
78
69
58
30
28
19
17
12
10
8
7
7
5
5
2
1
1
1
1
1
MDSeq
DiPhiSeq
Levene
DiffDist
GAMLSS
1500
1000
500
0
Set Size

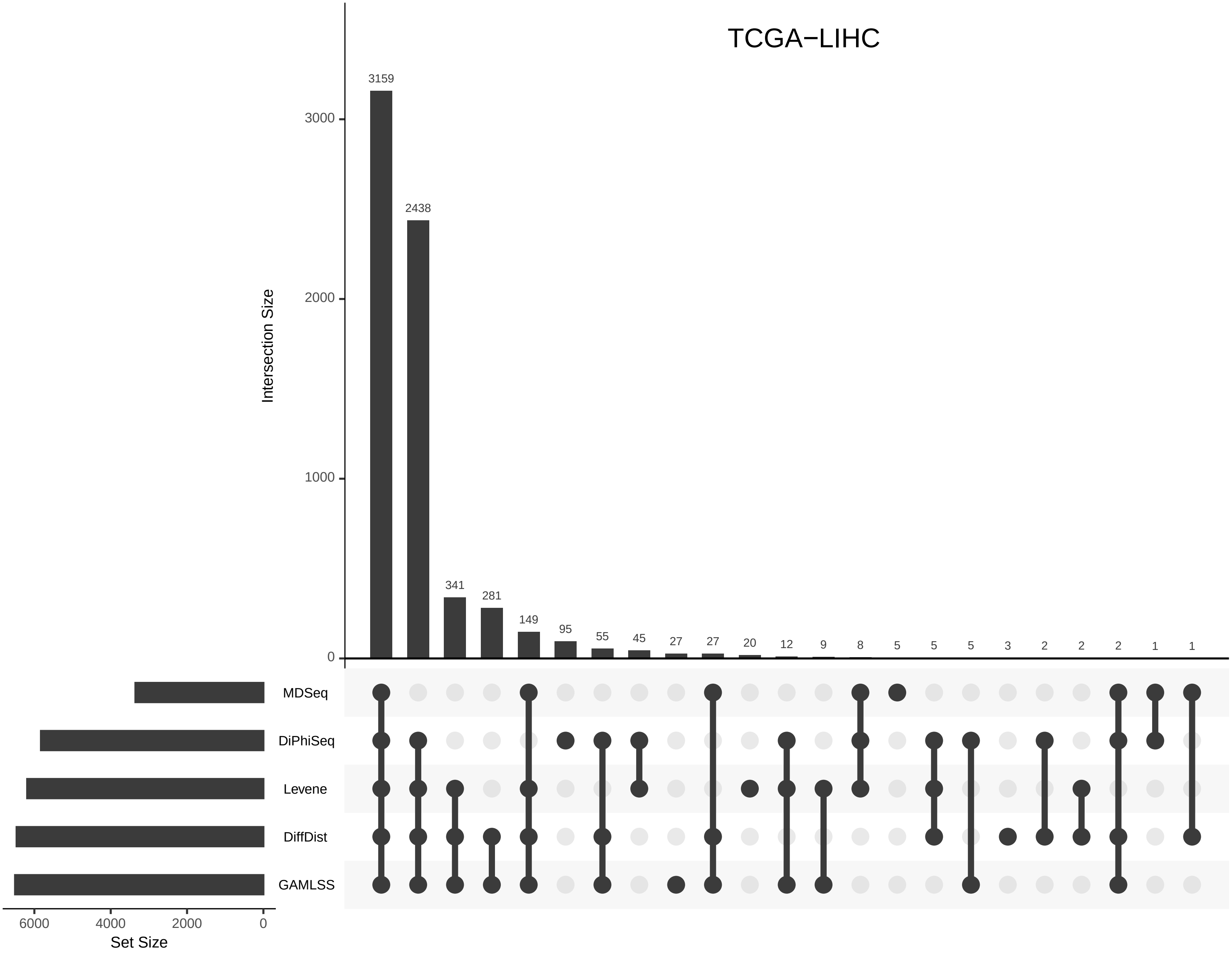
TCGA−LIHC
Intersection Size
3000
2000
1000
0
3159
2438
341
281
149
95
55
45
27
27
20
12
9
8
5
5
5
3
2
2
2
1
1
MDSeq
DiPhiSeq
Levene
DiffDist
GAMLSS
6000
4000
2000
0
Set Size

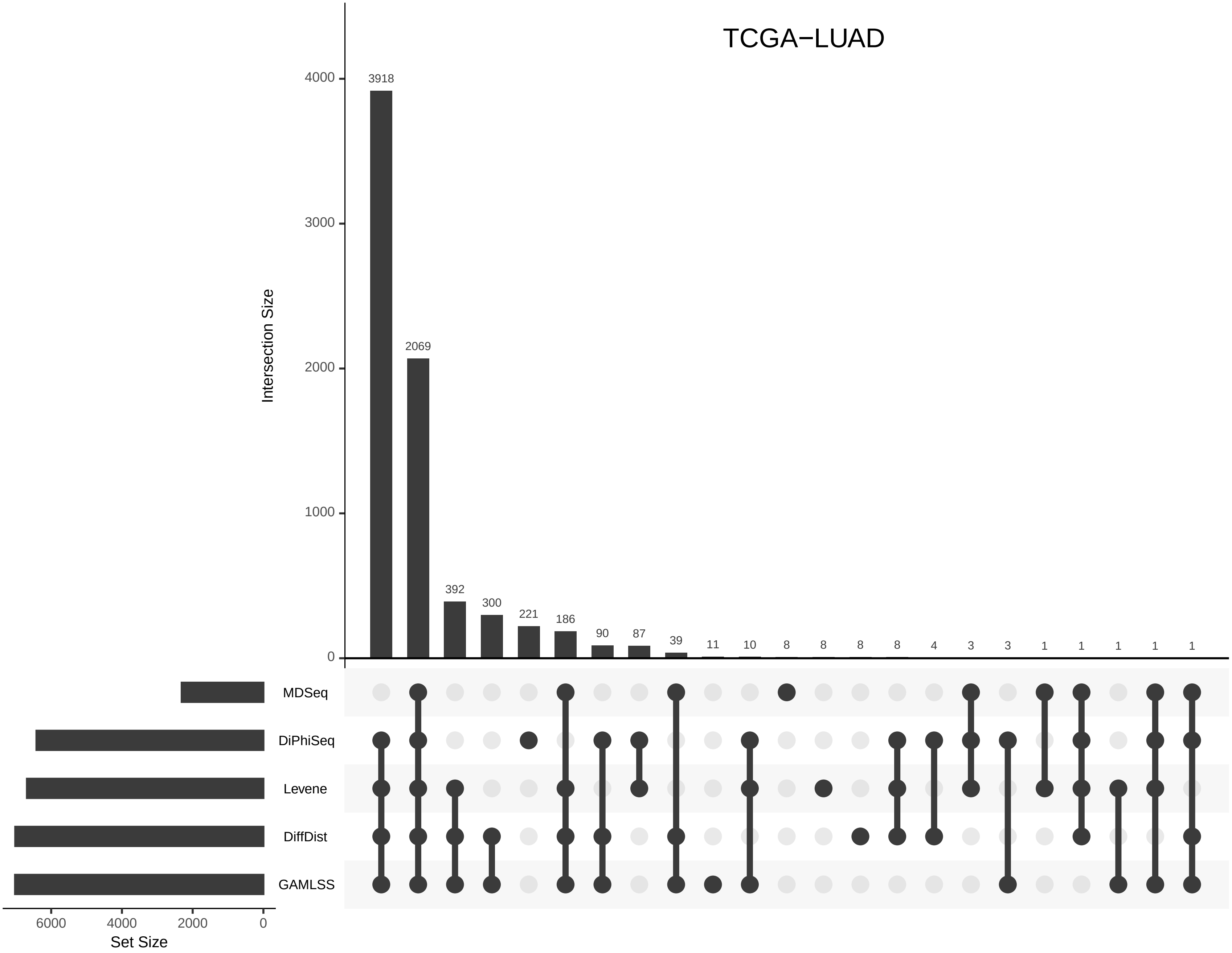
TCGA−LUAD
Intersection Size
0
1000
2000
3000
4000
3918
2069
392
300
221
186
90
87
39
11
10
8
8
8
8
4
3
3
1
1
1
1
1
MDSeq
DiPhiSeq
Levene
DiffDist
GAMLSS
6000
4000
2000
0
Set Size

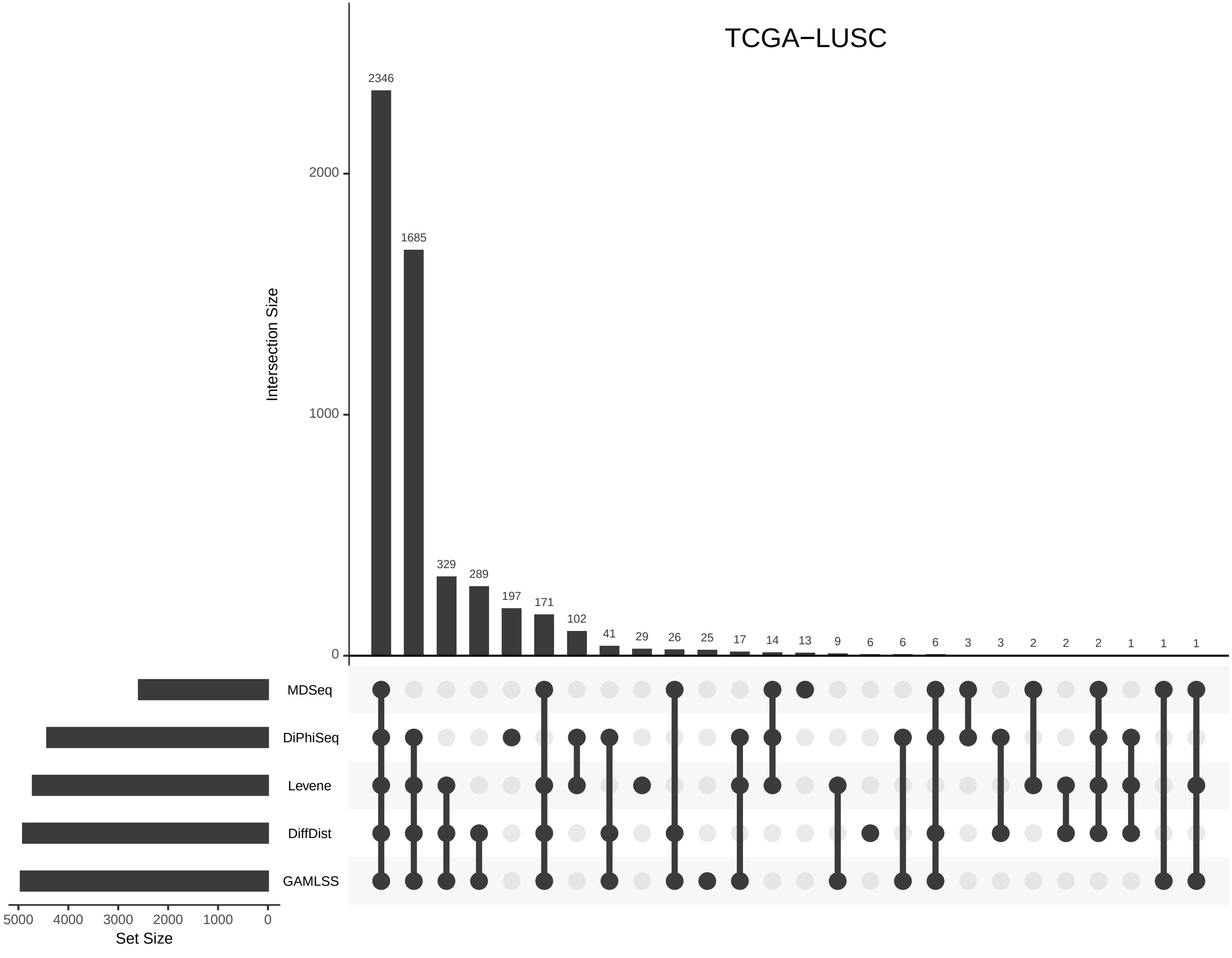

TCGA−LUSC
Intersection Size
2000
1000
0
2346
1685
329
289
197
171
102
41
29
26
25
17
14
13
9
6
6
6
3
3
2
2
2
1
1
1
MDSeq
DiPhiSeq
Levene
DiffDist
GAMLSS
5000
4000
3000
2000
1000
0
Set Size

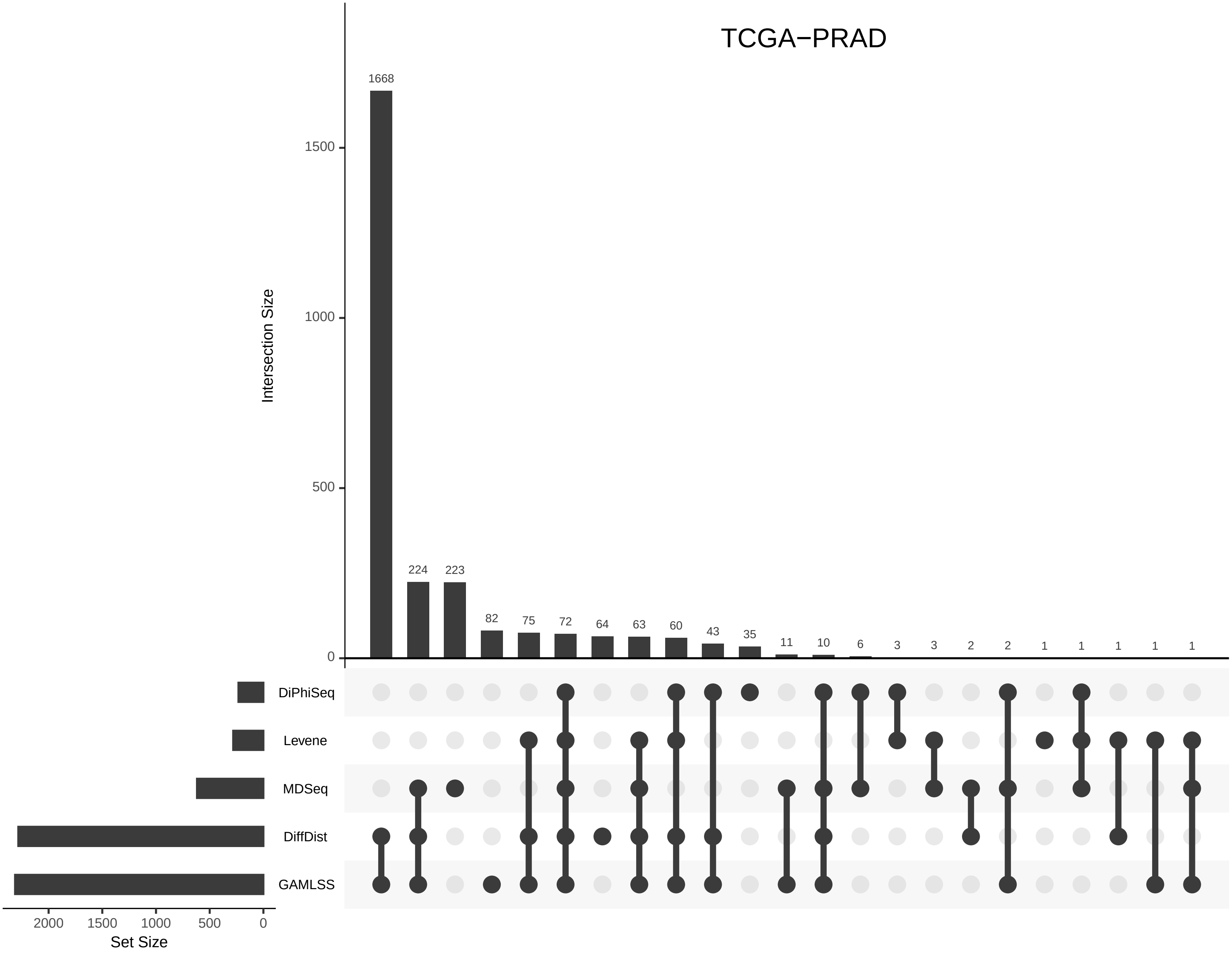
TCGA−PRAD
Intersection Size
1500
1000
500
0
1668
224
223
82
75
72
64
63
60
43
35
11
10
6
3
3
2
2
1
1
1
1
1
DiPhiSeq
Levene
MDSeq
DiffDist
GAMLSS
2000
1500
1000
500
0
Set Size

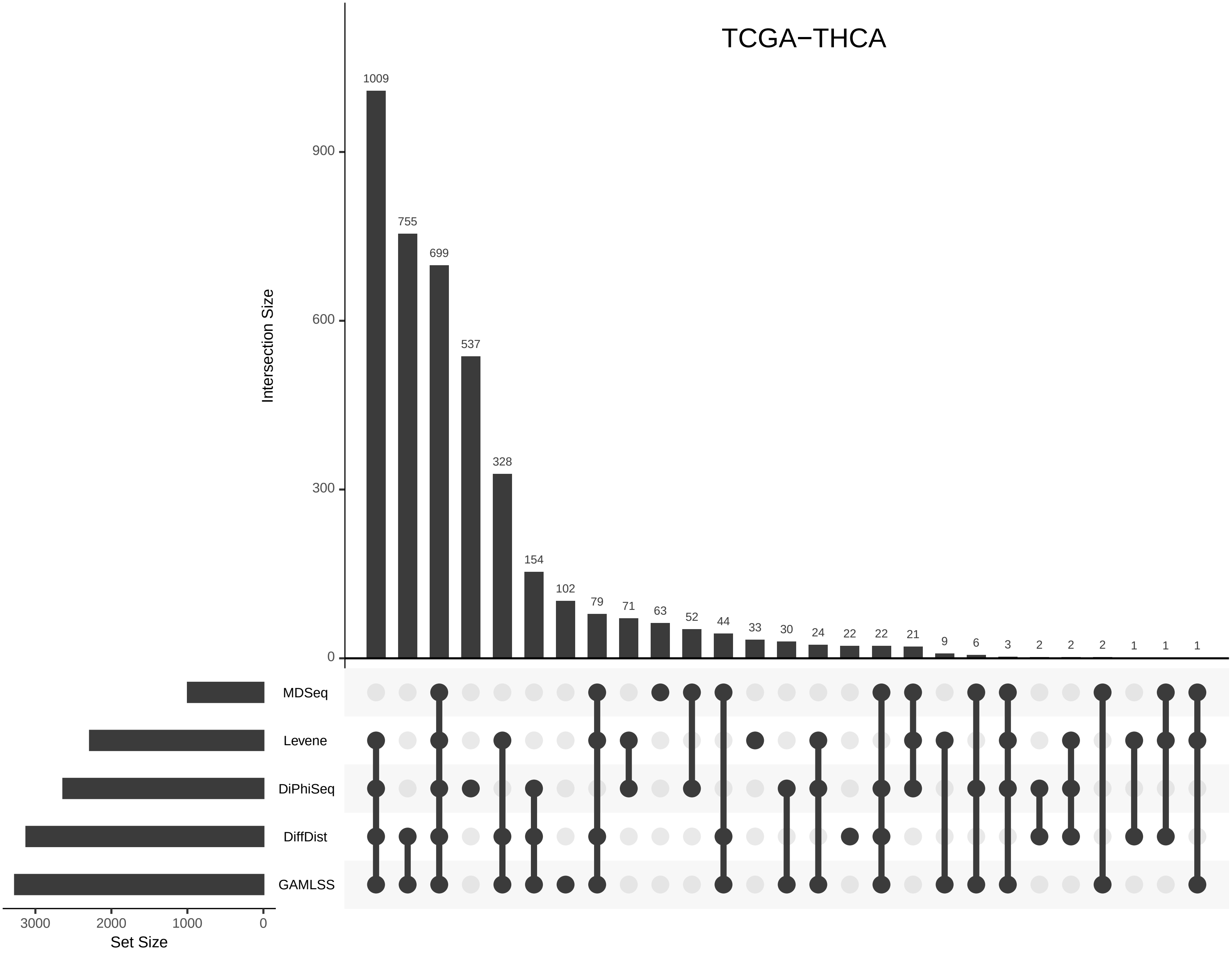

TCGA−THCA
Intersection Size
0
300
600
900
1009
755
699
537
328
154
102
79
71
63
52
44
33
30
24
22
22
21
9
6
3
2
2
2
1
1
1
MDSeq
Levene
DiPhiSeq
DiffDist
GAMLSS
3000
2000
1000
0
Set Size
