# Supplementary material for: Detection of genes with differential expression dispersion unravels the role of autophagy in cancer progression
Source: PLoS Comput Biol. 2023 Mar 9;19(3):e1010342. doi: 10.1371/journal.pcbi.1010342 (PMC9997931; doi:10.1371/journal.pcbi.1010342)
Supplement: S4 File — Full list of representative enriched Gene Ontology (GO) terms among upregulated genes in tumors, ordered first by the number of datasets for which they are enriched (decreasing order) and second by the mean p-values of enrichment across all datasets (increasing order). Upregulated genes were identified by MDSeq using a mean fold change threshold of 1. (PDF) [file pcbi.1010342.s011.pdf]

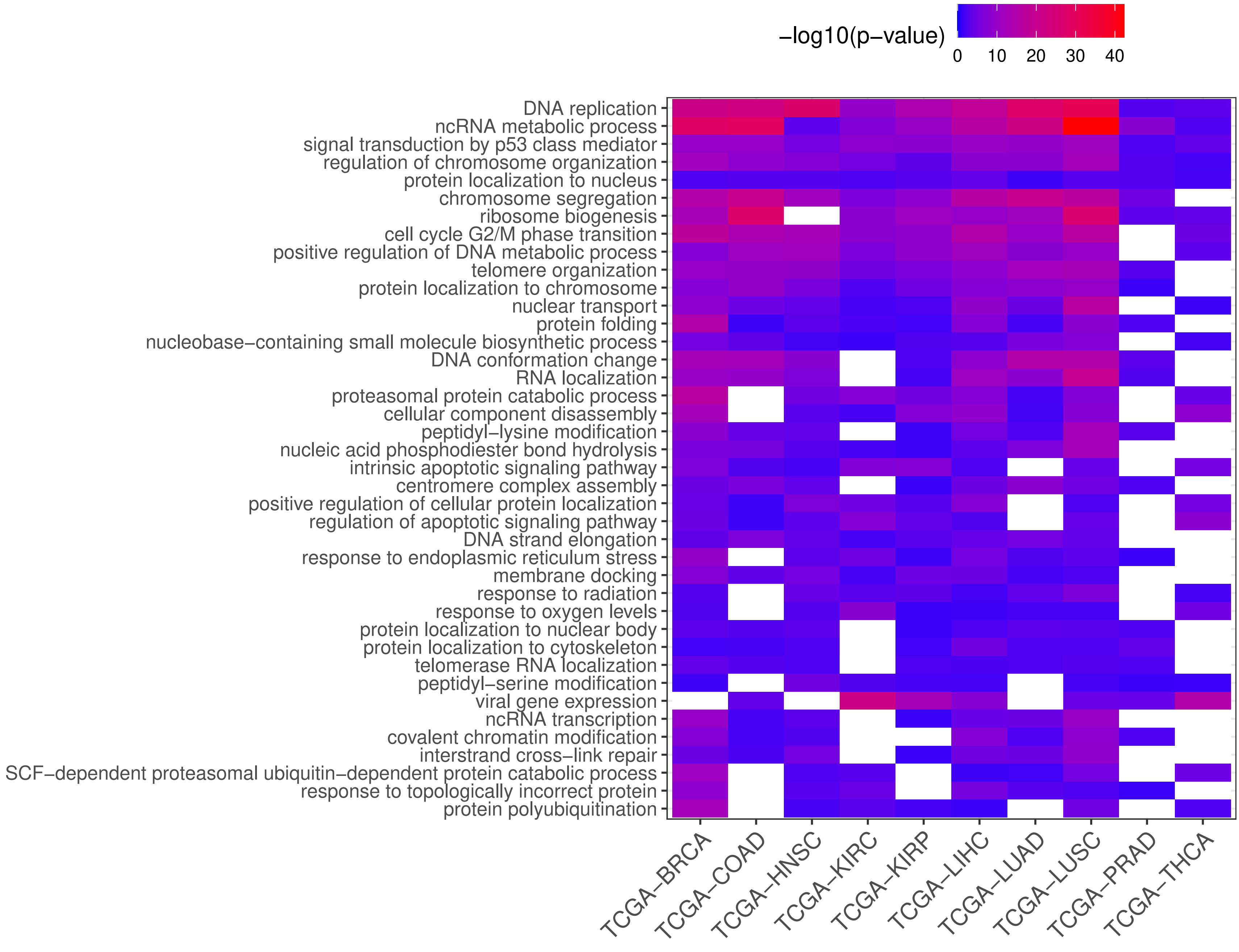

−log10(p−value)
0
10
20
30
40
DNA replication
ncRNA metabolic process
signal transduction by p53 class mediator
regulation of chromosome organization
protein localization to nucleus
chromosome segregation
ribosome biogenesis
cell cycle G2/M phase transition
positive regulation of DNA metabolic process
telomere organization
protein localization to chromosome
nuclear transport
protein folding
nucleobase−containing small molecule biosynthetic process
DNA conformation change
RNA localization
proteasomal protein catabolic process
cellular component disassembly
peptidyl−lysine modification
nucleic acid phosphodiester bond hydrolysis
intrinsic apoptotic signaling pathway
centromere complex assembly
positive regulation of cellular protein localization
regulation of apoptotic signaling pathway
DNA strand elongation
response to endoplasmic reticulum stress
membrane docking
response to radiation
response to oxygen levels
protein localization to nuclear body
protein localization to cytoskeleton
telomerase RNA localization
peptidyl−serine modification
viral gene expression
ncRNA transcription
covalent chromatin modification
interstrand cross−link repair
SCF−dependent proteasomal ubiquitin−dependent protein catabolic process
response to topologically incorrect protein
protein polyubiquitination
TCGA−BRCA
TCGA−COAD
TCGA−HNSC
TCGA−KIRC
TCGA−KIRP
TCGA−LIHC
TCGA−LUAD
TCGA−LUSC
TCGA−PRAD
TCGA−THCA

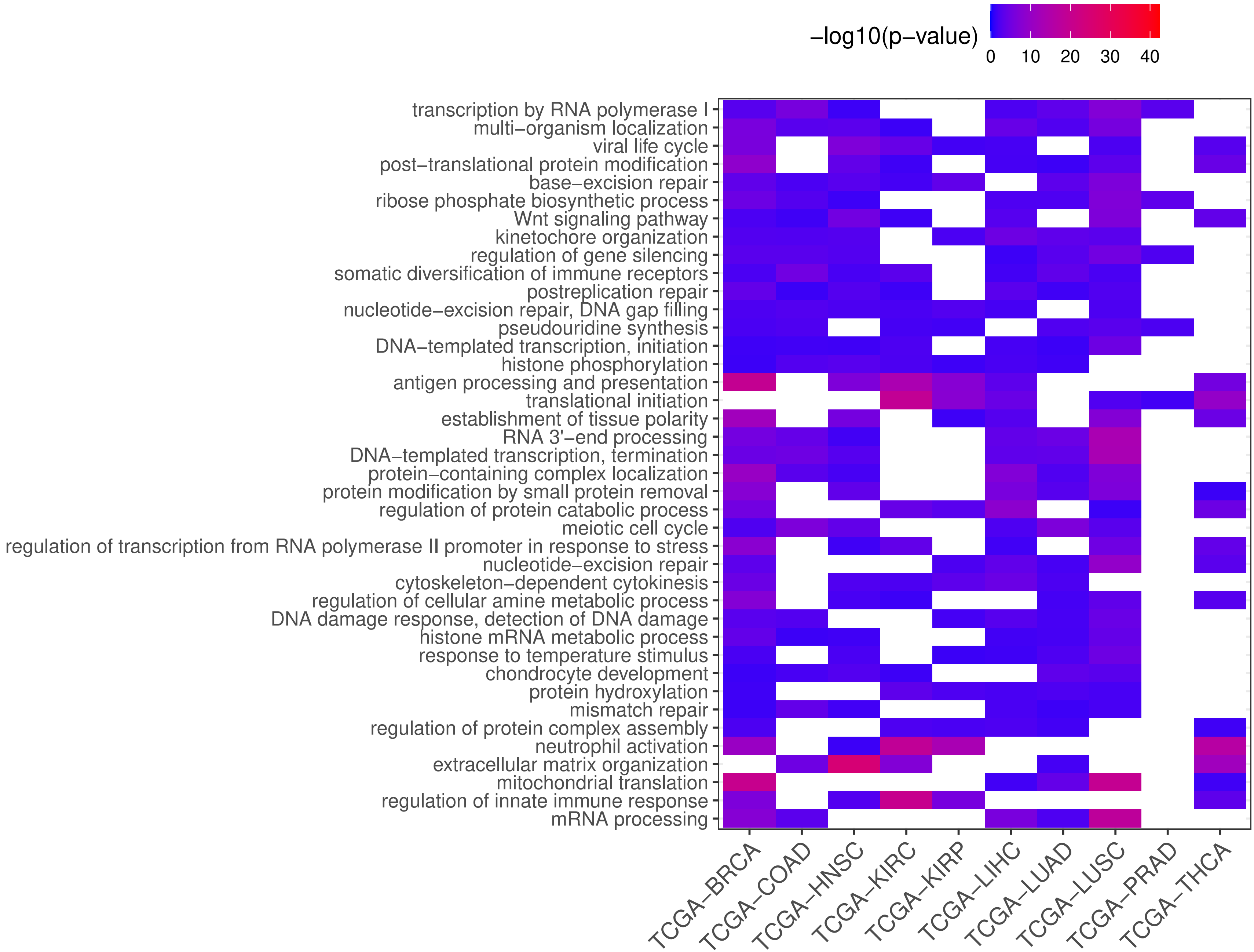

−log10(p−value)
0
10
20
30
40
transcription by RNA polymerase I
multi−organism localization
viral life cycle
post−translational protein modification
base−excision repair
ribose phosphate biosynthetic process
Wnt signaling pathway
kinetochore organization
regulation of gene silencing
somatic diversification of immune receptors
postreplication repair
nucleotide−excision repair, DNA gap filling
pseudouridine synthesis
DNA−templated transcription, initiation
histone phosphorylation
antigen processing and presentation
translational initiation
establishment of tissue polarity
RNA 3'−end processing
DNA−templated transcription, termination
protein−containing complex localization
protein modification by small protein removal
regulation of protein catabolic process
meiotic cell cycle
regulation of transcription from RNA polymerase II promoter in response to stress
nucleotide−excision repair
cytoskeleton−dependent cytokinesis
regulation of cellular amine metabolic process
DNA damage response, detection of DNA damage
histone mRNA metabolic process
response to temperature stimulus
chondrocyte development
protein hydroxylation
mismatch repair
regulation of protein complex assembly
neutrophil activation
extracellular matrix organization
mitochondrial translation
regulation of innate immune response
mRNA processing
TCGA−BRCA
TCGA−COAD
TCGA−HNSC
TCGA−KIRC
TCGA−KIRP
TCGA−LIHC
TCGA−LUAD
TCGA−LUSC
TCGA−PRAD
TCGA−THCA

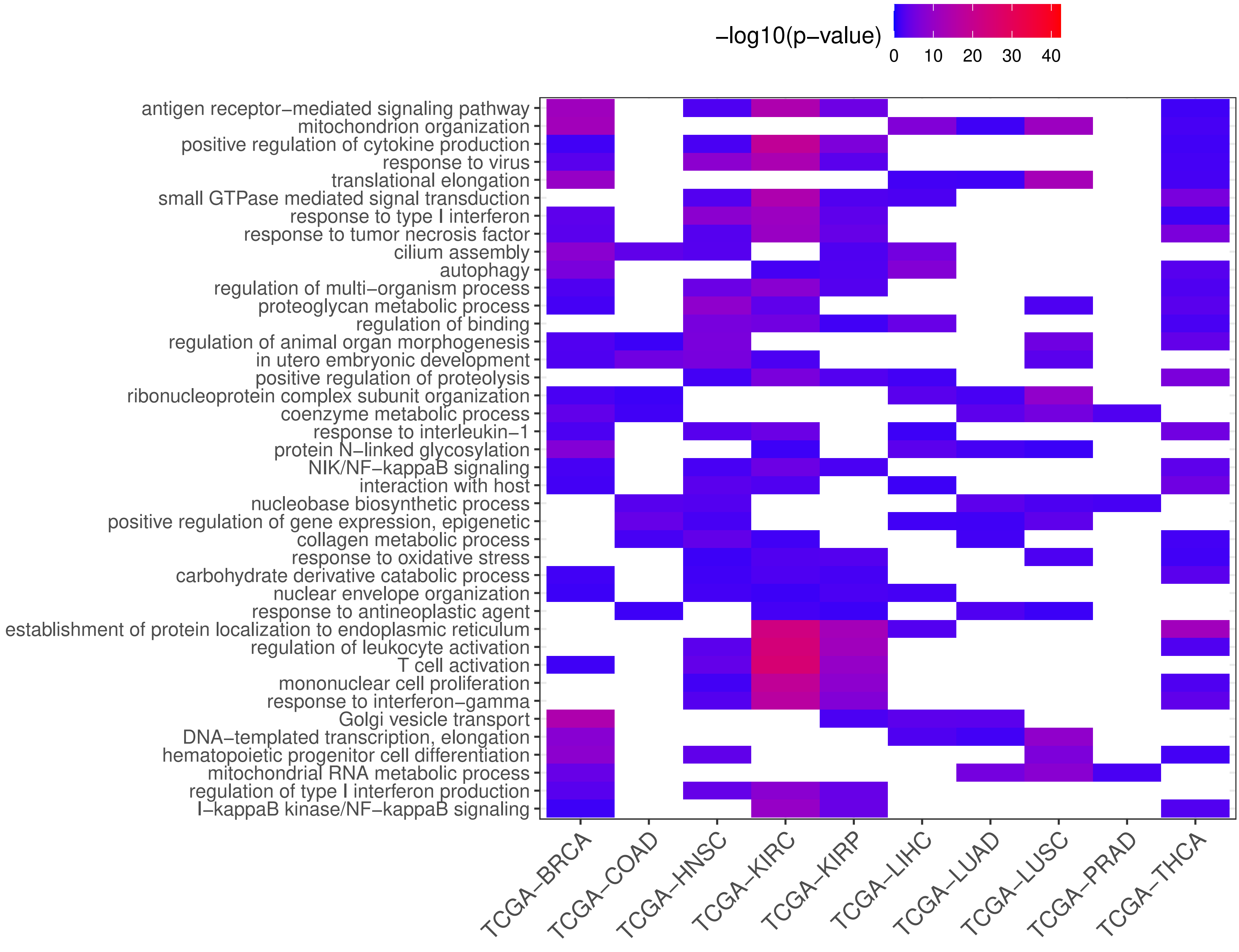

−log10(p−value)
0
10
20
30
40
antigen receptor−mediated signaling pathway
mitochondrion organization
positive regulation of cytokine production
response to virus
translational elongation
small GTPase mediated signal transduction
response to type I interferon
response to tumor necrosis factor
cilium assembly
autophagy
regulation of multi−organism process
proteoglycan metabolic process
regulation of binding
regulation of animal organ morphogenesis
in utero embryonic development
positive regulation of proteolysis
ribonucleoprotein complex subunit organization
coenzyme metabolic process
response to interleukin−1
protein N−linked glycosylation
NIK/NF−kappaB signaling
interaction with host
nucleobase biosynthetic process
positive regulation of gene expression, epigenetic
collagen metabolic process
response to oxidative stress
carbohydrate derivative catabolic process
nuclear envelope organization
response to antineoplastic agent
establishment of protein localization to endoplasmic reticulum
regulation of leukocyte activation
T cell activation
mononuclear cell proliferation
response to interferon−gamma
Golgi vesicle transport
DNA−templated transcription, elongation
hematopoietic progenitor cell differentiation
mitochondrial RNA metabolic process
regulation of type I interferon production
I−kappaB kinase/NF−kappaB signaling
TCGA−BRCA
TCGA−COAD
TCGA−HNSC
TCGA−KIRC
TCGA−KIRP
TCGA−LIHC
TCGA−LUAD
TCGA−LUSC
TCGA−PRAD
TCGA−THCA

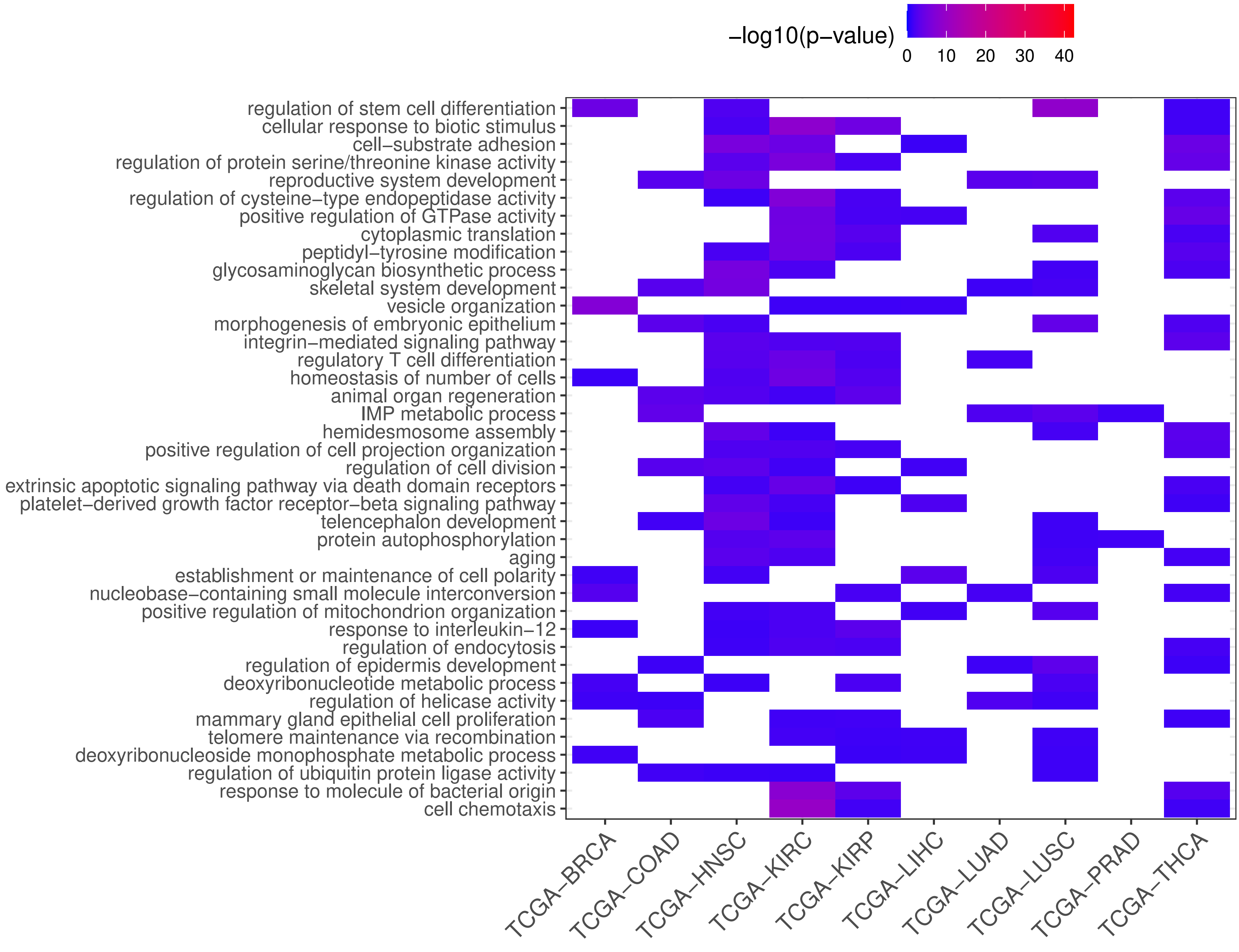

−log10(p−value)
0
10
20
30
40
regulation of stem cell differentiation
cellular response to biotic stimulus
cell−substrate adhesion
regulation of protein serine/threonine kinase activity
reproductive system development
regulation of cysteine−type endopeptidase activity
positive regulation of GTPase activity
cytoplasmic translation
peptidyl−tyrosine modification
glycosaminoglycan biosynthetic process
skeletal system development
vesicle organization
morphogenesis of embryonic epithelium
integrin−mediated signaling pathway
regulatory T cell differentiation
homeostasis of number of cells
animal organ regeneration
IMP metabolic process
hemidesmosome assembly
positive regulation of cell projection organization
regulation of cell division
extrinsic apoptotic signaling pathway via death domain receptors
platelet−derived growth factor receptor−beta signaling pathway
telencephalon development
protein autophosphorylation
aging
establishment or maintenance of cell polarity
nucleobase−containing small molecule interconversion
positive regulation of mitochondrion organization
response to interleukin−12
regulation of endocytosis
regulation of epidermis development
deoxyribonucleotide metabolic process
regulation of helicase activity
mammary gland epithelial cell proliferation
telomere maintenance via recombination
deoxyribonucleoside monophosphate metabolic process
regulation of ubiquitin protein ligase activity
response to molecule of bacterial origin
cell chemotaxis
TCGA−BRCA
TCGA−COAD
TCGA−HNSC
TCGA−KIRC
TCGA−KIRP
TCGA−LIHC
TCGA−LUAD
TCGA−LUSC
TCGA−PRAD
TCGA−THCA

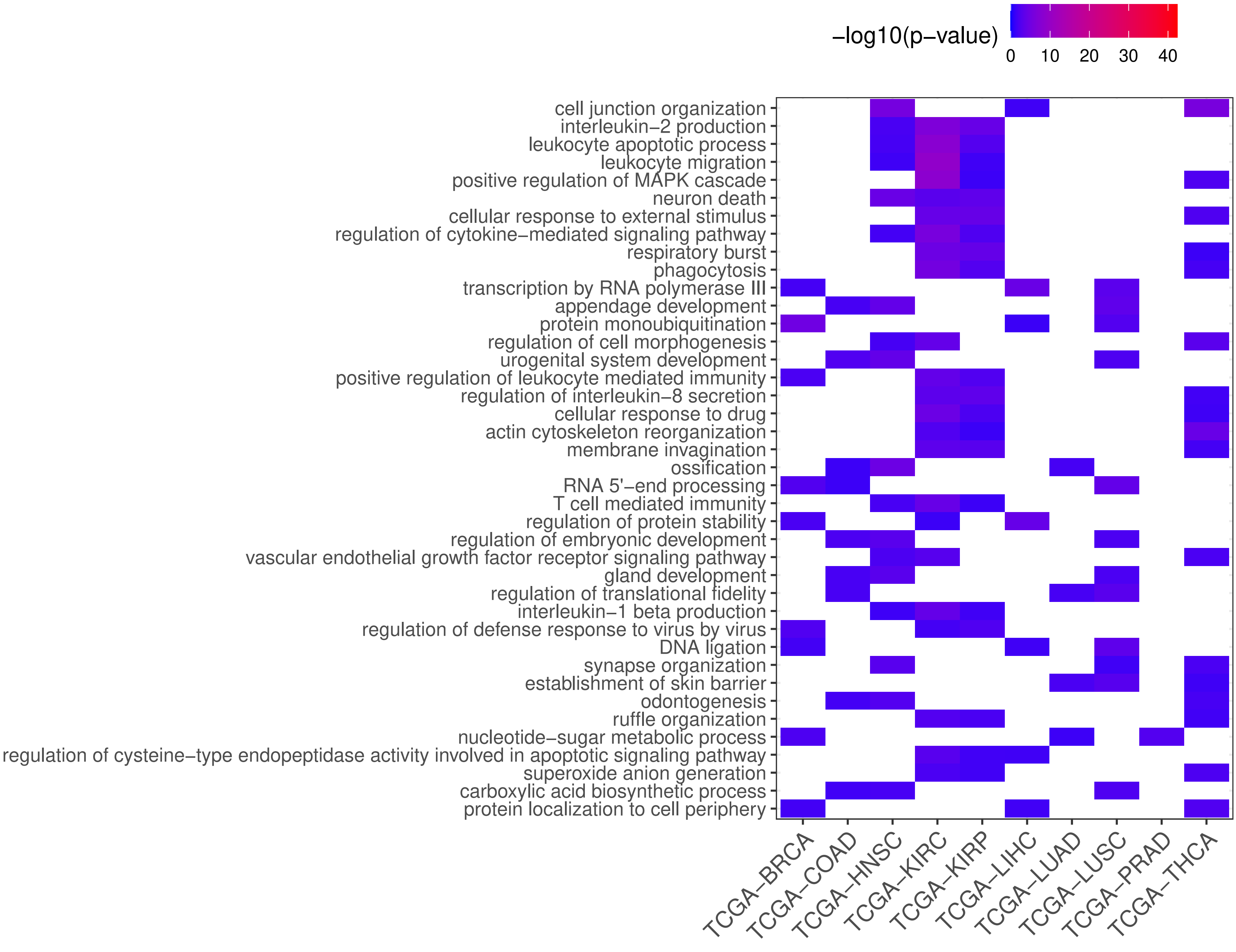

−log10(p−value)
0
10
20
30
40
cell junction organization
interleukin−2 production
leukocyte apoptotic process
leukocyte migration
positive regulation of MAPK cascade
neuron death
cellular response to external stimulus
regulation of cytokine−mediated signaling pathway
respiratory burst
phagocytosis
transcription by RNA polymerase III
appendage development
protein monoubiquitination
regulation of cell morphogenesis
urogenital system development
positive regulation of leukocyte mediated immunity
regulation of interleukin−8 secretion
cellular response to drug
actin cytoskeleton reorganization
membrane invagination
ossification
RNA 5'−end processing
T cell mediated immunity
regulation of protein stability
regulation of embryonic development
vascular endothelial growth factor receptor signaling pathway
gland development
regulation of translational fidelity
interleukin−1 beta production
regulation of defense response to virus by virus
DNA ligation
synapse organization
establishment of skin barrier
odontogenesis
ruffle organization
nucleotide−sugar metabolic process
regulation of cysteine−type endopeptidase activity involved in apoptotic signaling pathway
superoxide anion generation
carboxylic acid biosynthetic process
protein localization to cell periphery
TCGA−BRCA
TCGA−COAD
TCGA−HNSC
TCGA−KIRC
TCGA−KIRP
TCGA−LIHC
TCGA−LUAD
TCGA−LUSC
TCGA−PRAD
TCGA−THCA

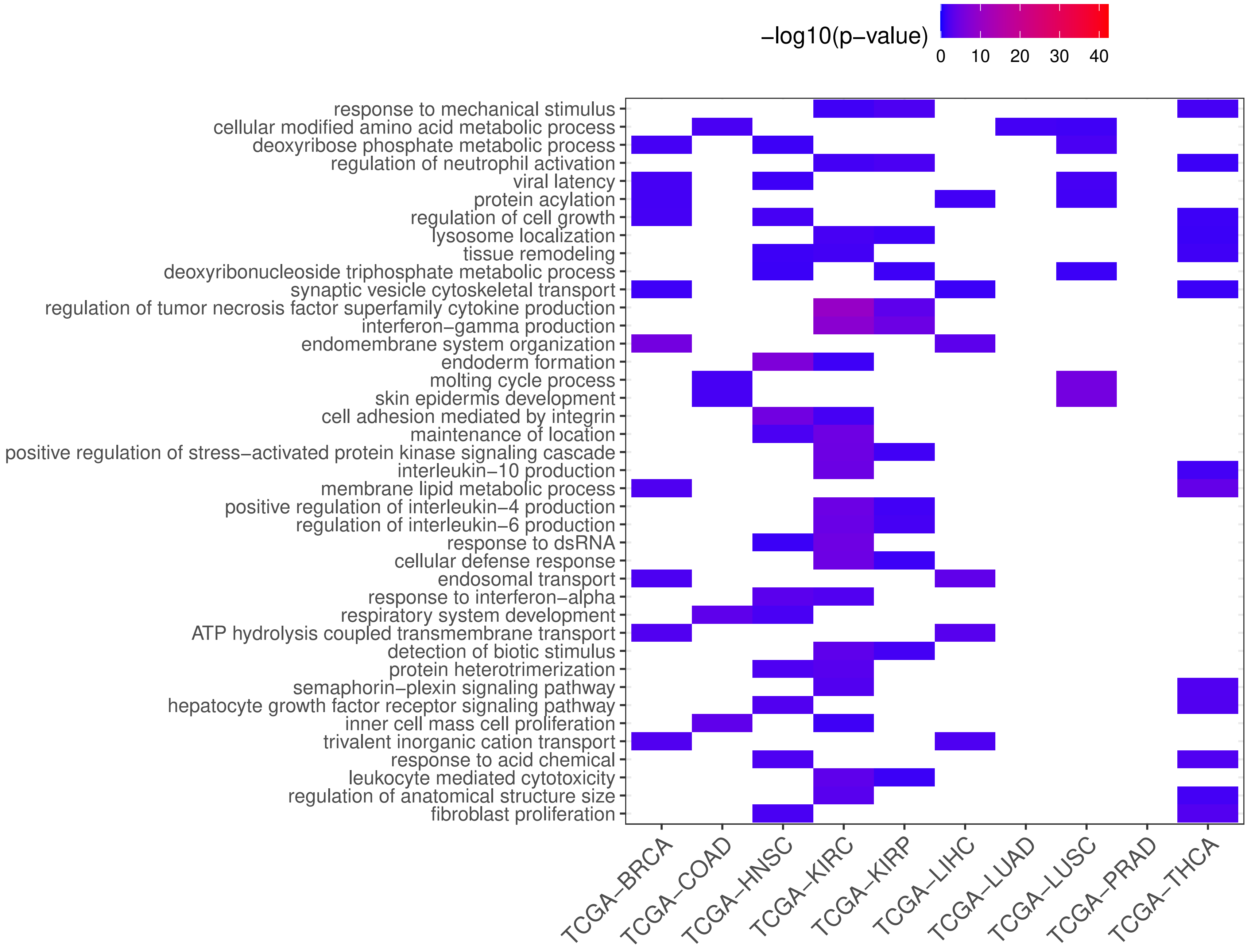

−log10(p−value)
0
10
20
30
40
response to mechanical stimulus
cellular modified amino acid metabolic process
deoxyribose phosphate metabolic process
regulation of neutrophil activation
viral latency
protein acylation
regulation of cell growth
lysosome localization
tissue remodeling
deoxyribonucleoside triphosphate metabolic process
synaptic vesicle cytoskeletal transport
regulation of tumor necrosis factor superfamily cytokine production
interferon−gamma production
endomembrane system organization
endoderm formation
molting cycle process
skin epidermis development
cell adhesion mediated by integrin
maintenance of location
positive regulation of stress−activated protein kinase signaling cascade
interleukin−10 production
membrane lipid metabolic process
positive regulation of interleukin−4 production
regulation of interleukin−6 production
response to dsRNA
cellular defense response
endosomal transport
response to interferon−alpha
respiratory system development
ATP hydrolysis coupled transmembrane transport
detection of biotic stimulus
protein heterotrimerization
semaphorin−plexin signaling pathway
hepatocyte growth factor receptor signaling pathway
inner cell mass cell proliferation
trivalent inorganic cation transport
response to acid chemical
leukocyte mediated cytotoxicity
regulation of anatomical structure size
fibroblast proliferation
TCGA−BRCA
TCGA−COAD
TCGA−HNSC
TCGA−KIRC
TCGA−KIRP
TCGA−LIHC
TCGA−LUAD
TCGA−LUSC
TCGA−PRAD
TCGA−THCA

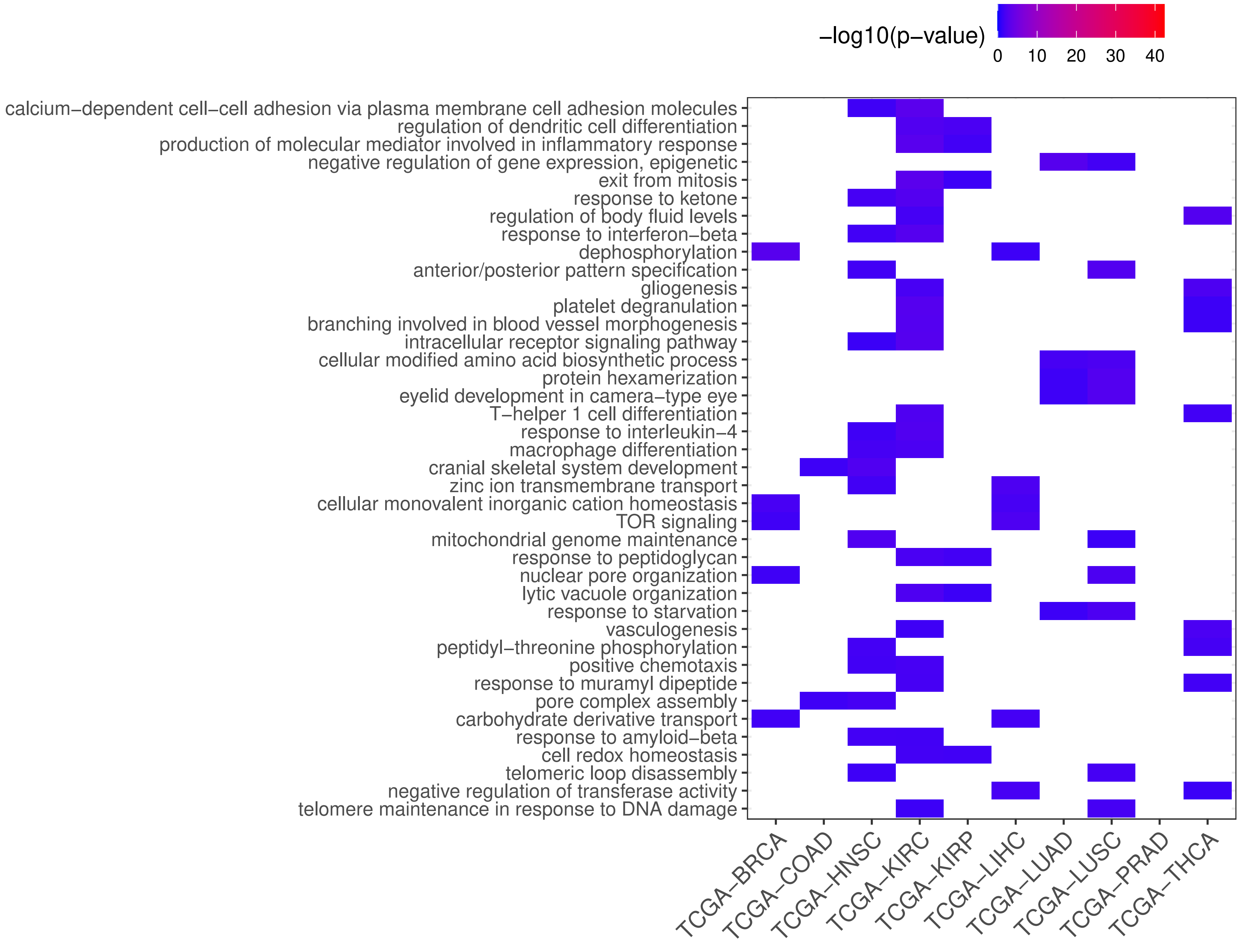
−log10(p−value)
0
10
20
30
40
calcium−dependent cell−cell adhesion via plasma membrane cell adhesion molecules
regulation of dendritic cell differentiation
production of molecular mediator involved in inflammatory response
negative regulation of gene expression, epigenetic
exit from mitosis
response to ketone
regulation of body fluid levels
response to interferon−beta
dephosphorylation
anterior/posterior pattern specification
gliogenesis
platelet degranulation
branching involved in blood vessel morphogenesis
intracellular receptor signaling pathway
cellular modified amino acid biosynthetic process
protein hexamerization
eyelid development in camera−type eye
T−helper 1 cell differentiation
response to interleukin−4
macrophage differentiation
cranial skeletal system development
zinc ion transmembrane transport
cellular monovalent inorganic cation homeostasis
TOR signaling
mitochondrial genome maintenance
response to peptidoglycan
nuclear pore organization
lytic vacuole organization
response to starvation
vasculogenesis
peptidyl−threonine phosphorylation
positive chemotaxis
response to muramyl dipeptide
pore complex assembly
carbohydrate derivative transport
response to amyloid−beta
cell redox homeostasis
telomeric loop disassembly
negative regulation of transferase activity
telomere maintenance in response to DNA damage
TCGA−BRCA
TCGA−COAD
TCGA−HNSC
TCGA−KIRC
TCGA−KIRP
TCGA−LIHC
TCGA−LUAD
TCGA−LUSC
TCGA−PRAD
TCGA−THCA

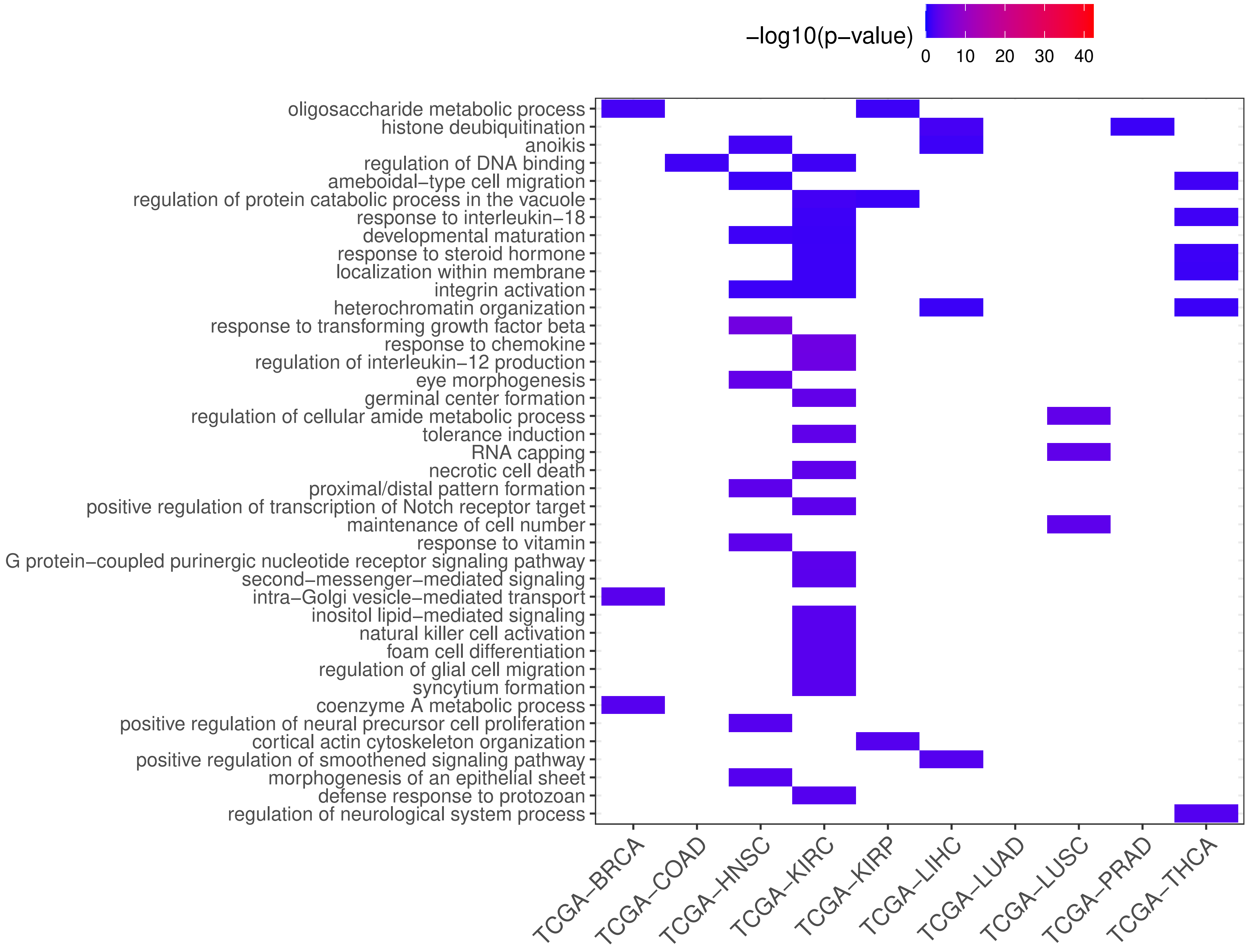

−log10(p−value)
0
10
20
30
40
oligosaccharide metabolic process
histone deubiquitination
anoikis
regulation of DNA binding
ameboidal−type cell migration
regulation of protein catabolic process in the vacuole
response to interleukin−18
developmental maturation
response to steroid hormone
localization within membrane
integrin activation
heterochromatin organization
response to transforming growth factor beta
response to chemokine
regulation of interleukin−12 production
eye morphogenesis
germinal center formation
regulation of cellular amide metabolic process
tolerance induction
RNA capping
necrotic cell death
proximal/distal pattern formation
positive regulation of transcription of Notch receptor target
maintenance of cell number
response to vitamin
G protein−coupled purinergic nucleotide receptor signaling pathway
second−messenger−mediated signaling
intra−Golgi vesicle−mediated transport
inositol lipid−mediated signaling
natural killer cell activation
foam cell differentiation
regulation of glial cell migration
syncytium formation
coenzyme A metabolic process
positive regulation of neural precursor cell proliferation
cortical actin cytoskeleton organization
positive regulation of smoothened signaling pathway
morphogenesis of an epithelial sheet
defense response to protozoan
regulation of neurological system process
TCGA−BRCA
TCGA−COAD
TCGA−HNSC
TCGA−KIRC
TCGA−KIRP
TCGA−LIHC
TCGA−LUAD
TCGA−LUSC
TCGA−PRAD
TCGA−THCA

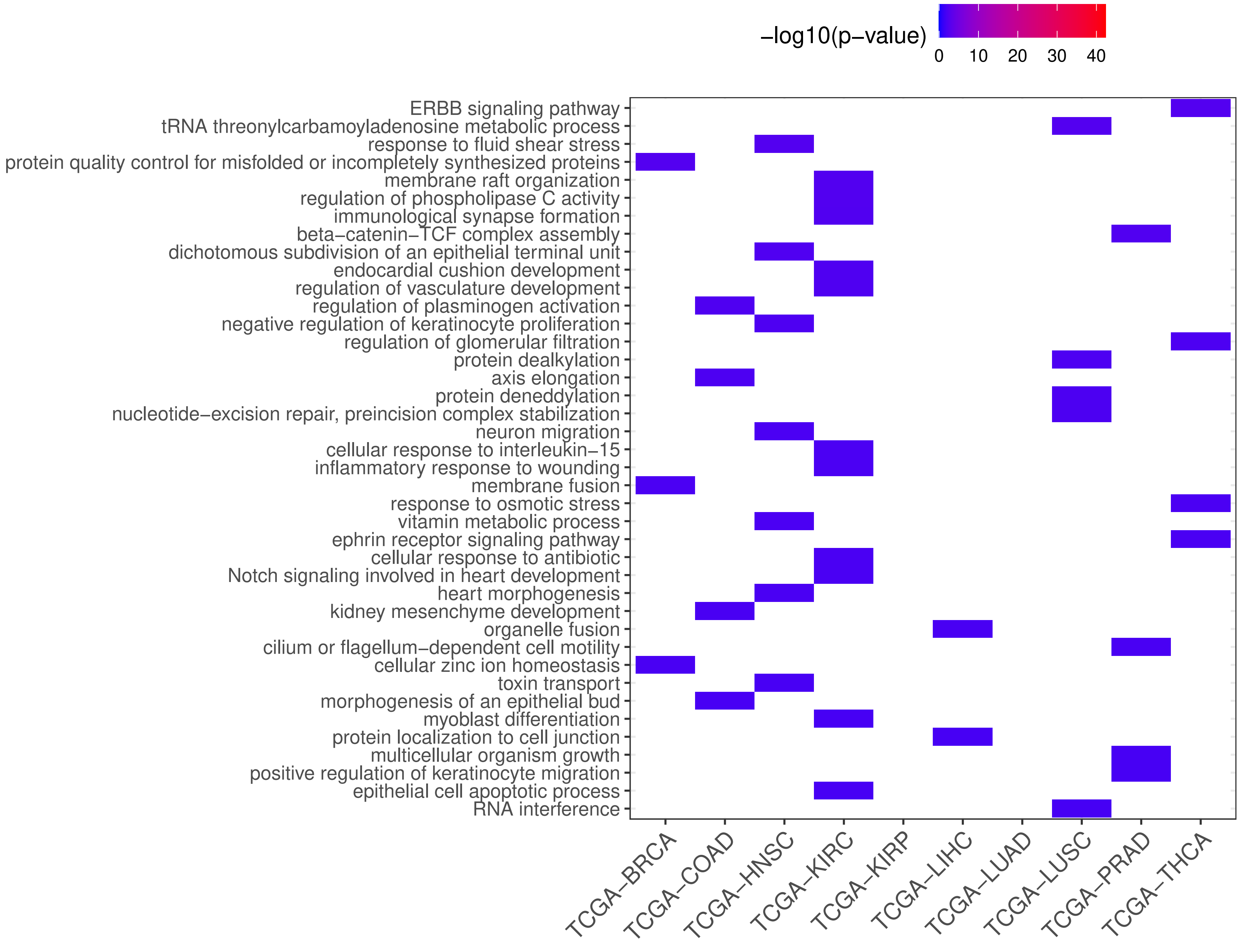

−log10(p−value)
0
10
20
30
40
ERBB signaling pathway
tRNA threonylcarbamoyladenosine metabolic process
response to fluid shear stress
protein quality control for misfolded or incompletely synthesized proteins
membrane raft organization
regulation of phospholipase C activity
immunological synapse formation
beta−catenin−TCF complex assembly
dichotomous subdivision of an epithelial terminal unit
endocardial cushion development
regulation of vasculature development
regulation of plasminogen activation
negative regulation of keratinocyte proliferation
regulation of glomerular filtration
protein dealkylation
axis elongation
protein deneddylation
nucleotide−excision repair, preincision complex stabilization
neuron migration
cellular response to interleukin−15
inflammatory response to wounding
membrane fusion
response to osmotic stress
vitamin metabolic process
ephrin receptor signaling pathway
cellular response to antibiotic
Notch signaling involved in heart development
heart morphogenesis
kidney mesenchyme development
organelle fusion
cilium or flagellum−dependent cell motility
cellular zinc ion homeostasis
toxin transport
morphogenesis of an epithelial bud
myoblast differentiation
protein localization to cell junction
multicellular organism growth
positive regulation of keratinocyte migration
epithelial cell apoptotic process
RNA interference
TCGA−BRCA
TCGA−COAD
TCGA−HNSC
TCGA−KIRC
TCGA−KIRP
TCGA−LIHC
TCGA−LUAD
TCGA−LUSC
TCGA−PRAD
TCGA−THCA

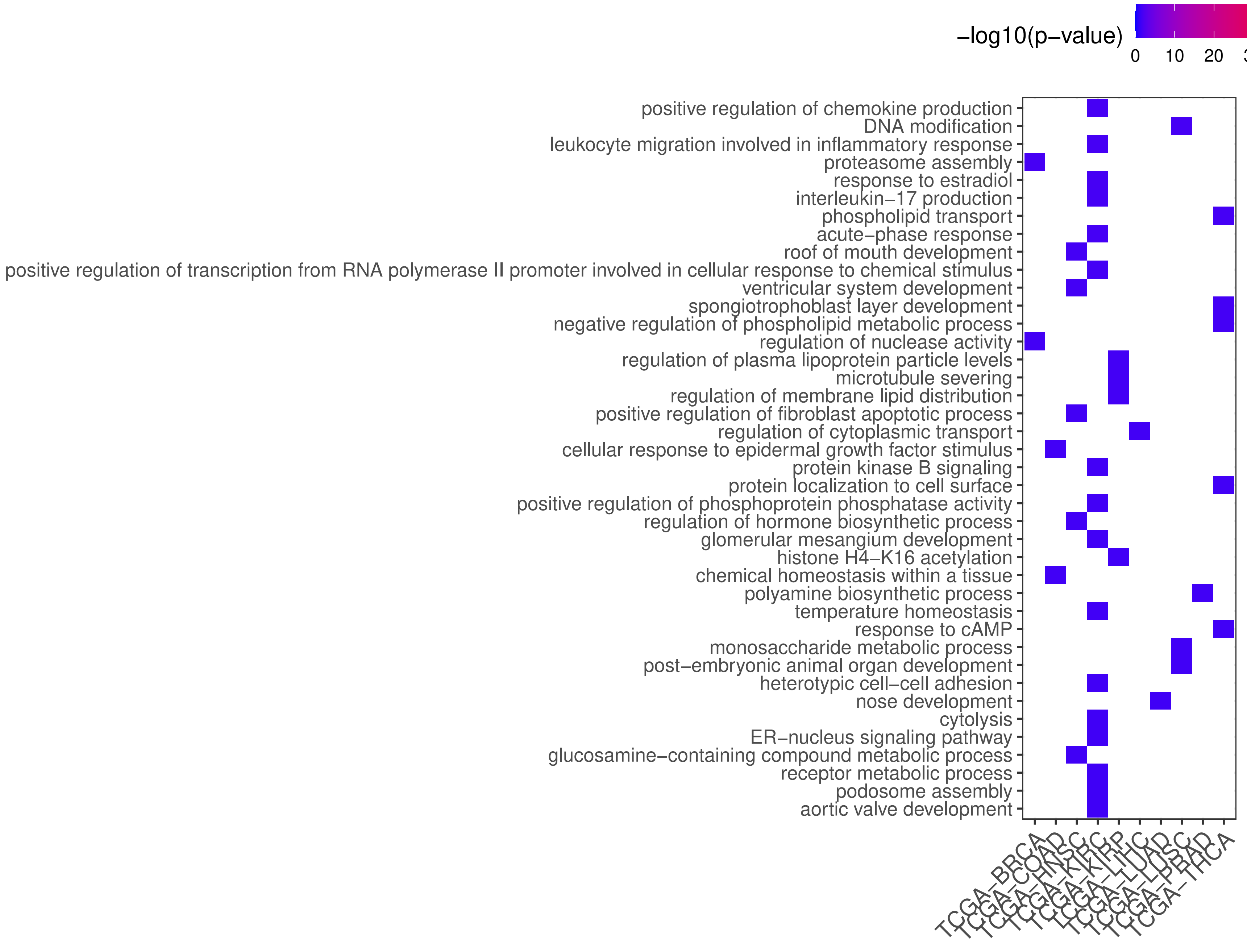

−log10(p−value)
0
10
20
positive regulation of chemokine production
DNA modification
leukocyte migration involved in inflammatory response
proteasome assembly
response to estradiol
interleukin−17 production
phospholipid transport
acute−phase response
roof of mouth development
positive regulation of transcription from RNA polymerase II promoter involved in cellular response to chemical stimulus
ventricular system development
spongiotrophoblast layer development
negative regulation of phospholipid metabolic process
regulation of nuclease activity
regulation of plasma lipoprotein particle levels
microtubule severing
regulation of membrane lipid distribution
positive regulation of fibroblast apoptotic process
regulation of cytoplasmic transport
cellular response to epidermal growth factor stimulus
protein kinase B signaling
protein localization to cell surface
positive regulation of phosphoprotein phosphatase activity
regulation of hormone biosynthetic process
glomerular mesangium development
histone H4−K16 acetylation
chemical homeostasis within a tissue
polyamine biosynthetic process
temperature homeostasis
response to cAMP
monosaccharide metabolic process
post−embryonic animal organ development
heterotypic cell−cell adhesion
nose development
cytolysis
ER−nucleus signaling pathway
glucosamine−containing compound metabolic process
receptor metabolic process
podosome assembly
aortic valve development
TCGA−BRCA
TCGA−COAD
TCGA−HNSC
TCGA−KIRC
TCGA−KIRP
TCGA−LIHC
TCGA−LUAD
TCGA−LUSC
TCGA−PRAD
TCGA−THCA

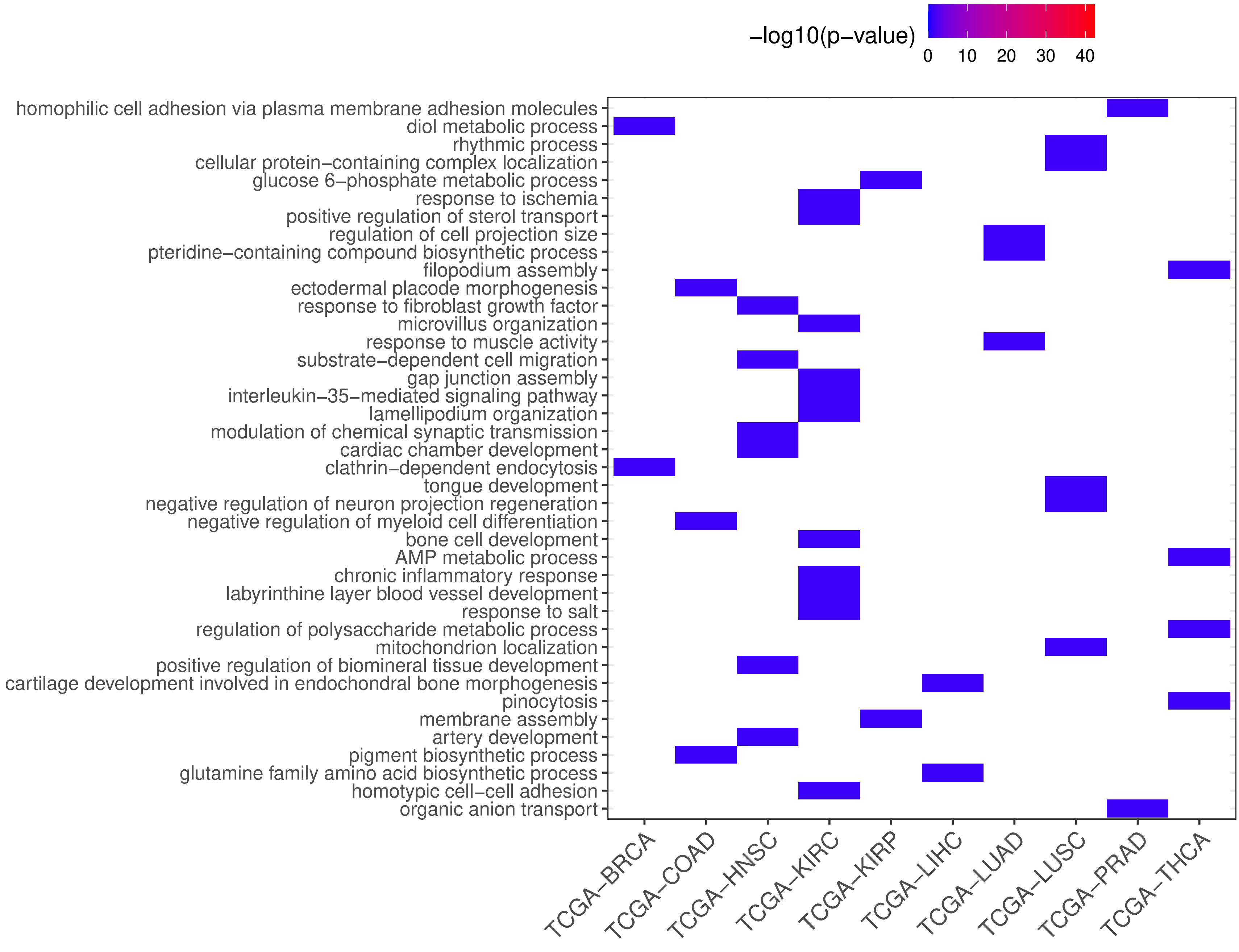
−log10(p−value)
0
10
20
30
40
homophilic cell adhesion via plasma membrane adhesion molecules
diol metabolic process
rhythmic process
cellular protein−containing complex localization
glucose 6−phosphate metabolic process
response to ischemia
positive regulation of sterol transport
regulation of cell projection size
pteridine−containing compound biosynthetic process
filopodium assembly
ectodermal placode morphogenesis
response to fibroblast growth factor
microvillus organization
response to muscle activity
substrate−dependent cell migration
gap junction assembly
interleukin−35−mediated signaling pathway
lamellipodium organization
modulation of chemical synaptic transmission
cardiac chamber development
clathrin−dependent endocytosis
tongue development
negative regulation of neuron projection regeneration
negative regulation of myeloid cell differentiation
bone cell development
AMP metabolic process
chronic inflammatory response
labyrinthine layer blood vessel development
response to salt
regulation of polysaccharide metabolic process
mitochondrion localization
positive regulation of biomineral tissue development
cartilage development involved in endochondral bone morphogenesis
pinocytosis
membrane assembly
artery development
pigment biosynthetic process
glutamine family amino acid biosynthetic process
homotypic cell−cell adhesion
organic anion transport
TCGA−BRCA
TCGA−COAD
TCGA−HNSC
TCGA−KIRC
TCGA−KIRP
TCGA−LIHC
TCGA−LUAD
TCGA−LUSC
TCGA−PRAD
TCGA−THCA

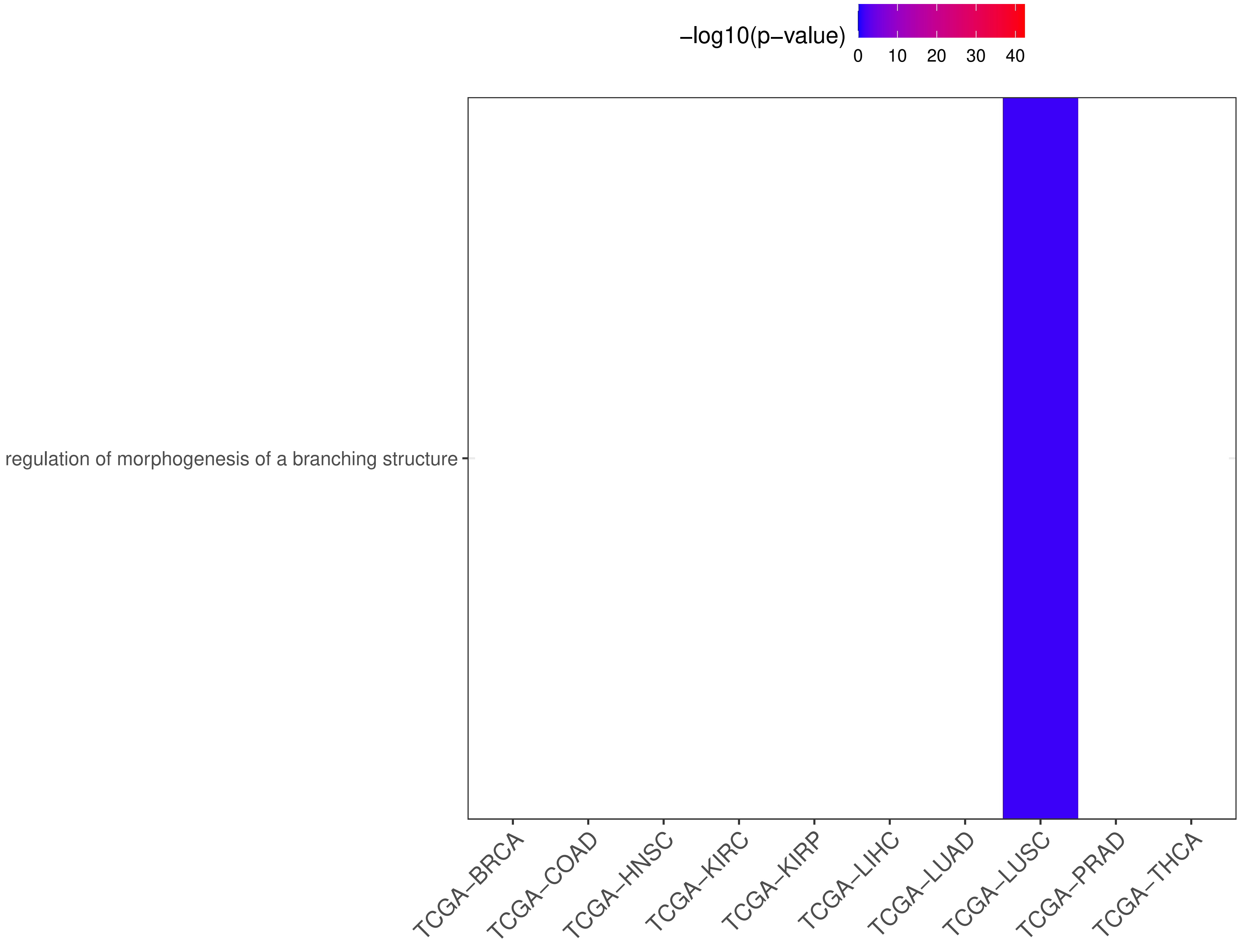

−log10(p−value)
0
10
20
30
40
regulation of morphogenesis of a branching structure
TCGA−BRCA
TCGA−COAD
TCGA−HNSC
TCGA−KIRC
TCGA−KIRP
TCGA−LIHC
TCGA−LUAD
TCGA−LUSC
TCGA−PRAD
TCGA−THCA
