# Supplementary material for: Detection of genes with differential expression dispersion unravels the role of autophagy in cancer progression
Source: PLoS Comput Biol. 2023 Mar 9;19(3):e1010342. doi: 10.1371/journal.pcbi.1010342 (PMC9997931; doi:10.1371/journal.pcbi.1010342)
Supplement: S2 Fig — False discovery rate (FDR) and true positive rate (TPR) of Levene’s test, MDSeq, DiPhiSeq, GAMLSS and DiffDist for differential dispersion detection in simulated datasets composed of lowly differentially expressed genes between two sample populations of equal size. The Benjamini-Hochberg procedure was applied to control the FDR for all the methods. The performances were assessed using 10 replicates of simulated datasets per parameter setting. (PDF) [file pcbi.1010342.s002.pdf]

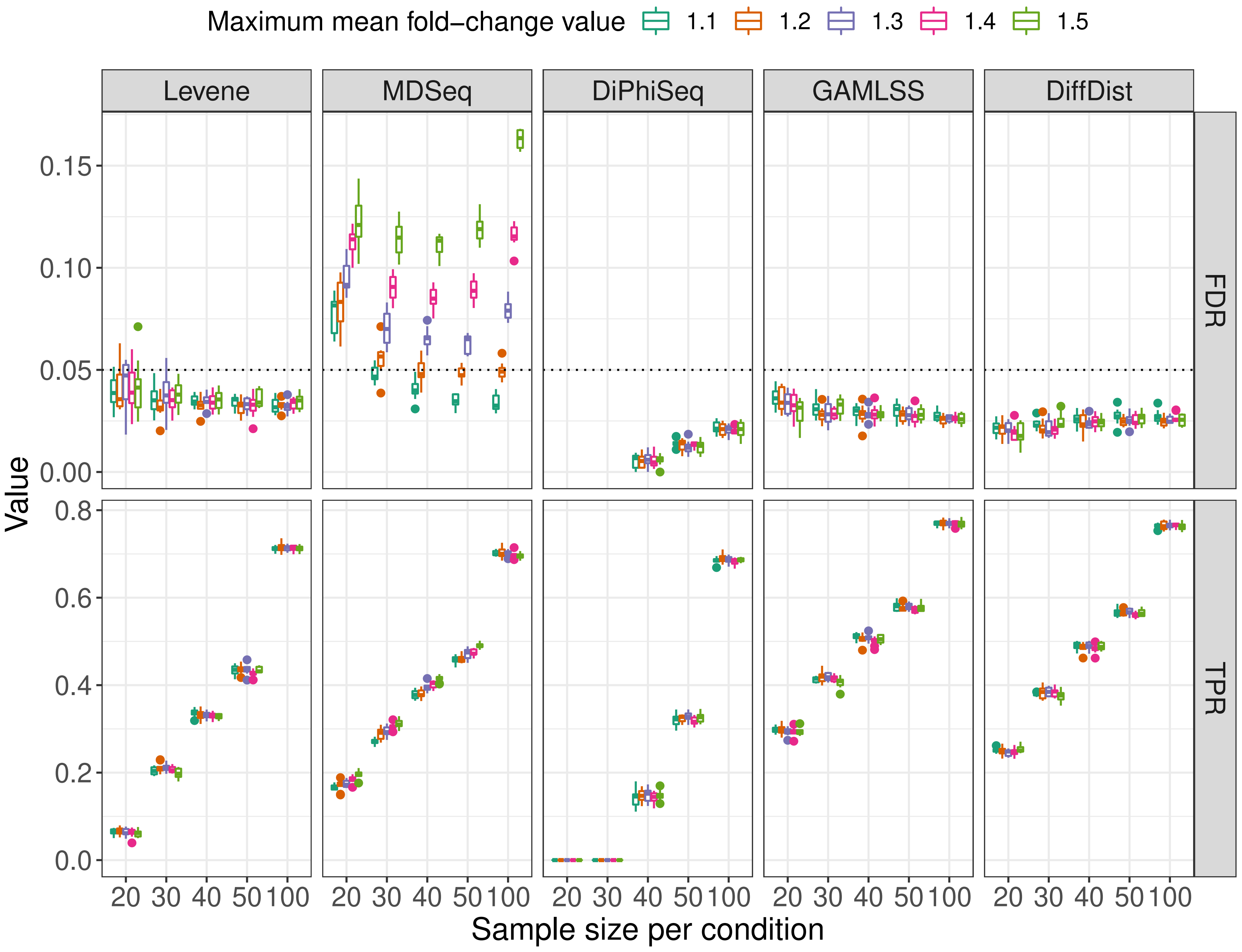

Maximum mean fold−change value
1.1
1.2
1.3
1.4
1.5
Levene
MDSeq
DiPhiSeq
GAMLSS
DiffDist
FDR
TPR
Value
0.00
0.05
0.10
0.15
0.0
0.2
0.4
0.6
0.8
20
30
40
50
100
Sample size per condition
