# Supplementary material for: Detection of genes with differential expression dispersion unravels the role of autophagy in cancer progression
Source: PLoS Comput Biol. 2023 Mar 9;19(3):e1010342. doi: 10.1371/journal.pcbi.1010342 (PMC9997931; doi:10.1371/journal.pcbi.1010342)
Supplement: Attachment — Submitted filename: response_to_reviewers.pdf [file pcbi.1010342.s013.pdf]

Responses to comments

We would like to thank all the reviewers for their constructive comments and suggestions which helped us to improve the manuscript. Please find below our point-by-point responses to each individual comment.

Reviewer 1

> *The work is very thorough but I'm not sure why the authors felt that MDSeq and DiPhiSeq were important to evaluate. Have these two methods made such an impact on the subfield that they deserve to be studied and compared? Have there been no other methods proposed since then that also deserve to included in the comparison? I'm not convinced that these two methods are important to study.*

We decided to include MDSeq and DiPhiSeq since they were the two only available methods enabling to identify differences of dispersion in RNA-seq data to our knowledge at the time we performed our study. Meanwhile, two new methods which enable to achieve the same goal were published. First, de Jong *et al* proposed to take benefits of a generalized additive model for location, scale and shape (GAMLSS), rather than a generalized linear model, to enable the estimation of dispersion for different groups of samples [1]. Second, Roberts *et al* introduced a new dedicated method, DiffDist, which enables the identification of any change of distribution in RNA-seq data, including changes of dispersion [2]. In this revised version of our manuscript, we included these two new approaches in our study in order to make it as comprehensive as possible. We believe that the increase of available statistical methods to identify differences of dispersion in RNA-seq data highlights the overall interest on this type of gene expression variation.

> *The study itself also does not include any simple tests for differential variance. For completeness I would be interested in seeing how something like a simple permutation approach using a statistic with power toward differential variances instead of differential expression would perform.*

In addition to the two previously cited methods, we also included Levene's test [3] in this new version of the study. Rather than following a permutation approach as suggested here, we estimated the performance of this statistical test of equality of variances by analyzing the datasets we simulated in order to have the same framwork as the other evaluated methods. We chose Levene's test since it is a robust alternative to the F-test for non normal data and the expression of large sets of genes shows departure from normality after $\log_2$-transformation of RNA-seq counts.

> *The authors apply these methods to gain into cancer. However, I'm not sure this gives me any additional insight about the methods*

comparison. The biological results generate interesting hypotheses, but there is limited validation of the results and in any case the purpose of this report doesn't seem to be to produce new biological insights.

The purpose of the application of the evaluated methods to TCGA datasets was only to highlight the potential benefit of looking for genes with differences of dispersion in their expression between conditions of interest, in addition to the classical approach of looking for changes in mean expression. Our goal was indeed to generate hypotheses, experimental validation was out of the scope of our study. However, we disagree on the absence of new biological insights. If one considers the gain of dispersion of the expression of one gene, as a dysregulation process, from our perspective and in agreement with reviewer 2, we have made two important biological discoveries. Of course, this will need experimental confirmation, yet these hypotheses are very interesting. First, catabolic pathways are more dysregulated in cancer than anabolic pathways. To the best of our knowledge, this is new and that could explain several features observed in tumor progression or even relapse. Indeed, as suggested by Kitano, more dysregulation may facilitates evolvability and adaptation to anticancer treatment. Second, autophagy, a major recycling process in the cell, is completely dysregulated in cancer, which is also very interesting as damaged cell parts could accumulate in the cancer cell.

Reviewer 2

1. Further clarification is needed on the choice of the methods used for comparison- DiPhiSeq and MDSeq. What kind of literature search was involved in making sure that these are the only methods available for calculating differential dispersion/distributions.

This concern was also raised by reviewer 1. Please refer to our response to his first comment.

2. How do different preprocessing methods (normalization, filtering etc.) impact the downstream results from the two tools? For example, in this case, TMM was used to normalize. If other methods of normalization or different thresholds for filtering were used, would you expect the methods to behave differently?

The main goal of our study was to identify genes whose expression has a difference of dispersion between samples of conditions of interest. We built our framework so as to set the methods and parameters of all the other steps, including the normalization of RNA-seq counts, to constant values. The TMM method was shown to perform the best in a comprehensive evaluation of normalization methods [4]. In particular, the TMM method was among the methods which minimized the intra-condition variance the most and, more importantly, was the method which introduced the less variation in the expression of a set

of housekeeping genes. For these reasons, the TMM method appeared to us to be a reasonable choice in the context of the identification of differences of disperion in gene expression. Besides, evaluating other normalization methods, in addition to the different differential dispersion methods and sample sizes, would have dramatically increased the overall settings to explore and the associated computations of our simulation study.

> *3. In lines 119-125, you mention the different methods used for adjustment for multiple testing. How do the methods compare if the same FDR adjustment method was used- BH/BY/Bonferroni? Please elaborate this sentence- "We note, however, that in our evaluation (see S2 Fig), the Benjamini-Hochberg procedure was not sufficient to control for FDR".*

We also evaluated the performance of MDSeq by using the Benjamini-Hochberg procedure to control the FDR for all the simulated datasets (see the new S1 Fig and S2 Fig). The FDR was effectively maintained below 0.05 with this procedure for only a few datasets while we observed high values for the others, some of them being higher than 0.1. Thus, the more conservative choice of FDR-controlling procedure made by the authors of MDSeq was confirmed by our simulation study.

> *4. It is important to provide some recommendations for the usage of either of the two methods. Do you suggest that they be used in conjunction to DE? Or consecutively?*

We provided some recommendations in the revised version of our manuscript for the identification of differentially dispersed genes. We think that the classical differential expression analysis and the identification of differentially dispersed genes are different but complementary approaches. Thus, performing one of these analysis, or both, depends on the goals of a study. The emergence of methods to identify differences of dispersion in RNA-seq counts makes the second type of approach possible, as we showed in our simulation study. In the case of identifying differences of both mean and dispersion, the differential distribution approach of DiffDist, combining the detection of any difference of both parameters in a single test, would be advantageous.

> *5. In lines 140-141, MDSeq is suggested to be used in lowly DE genes. You provide fold change thresholds based on your simulation studies. How should these be interpreted for tissues or conditions where gene expression is relatively low or the effects are subtle?*

We changed the set of genes among which to look for differentially dispersed genes in the revised version of our manuscript. We focused on the non-differentially expressed genes, since it is the most interesting set of genes among which to look for differentially dispersed genes, rather than extending the set to lowly differentially expressed (DE) genes according to the maximum mean fold changes

tolerated by MDSeq. We think that this choice is biologically more relevant. Besides, the inclusion of several other methods in our study and the relatively poor performance of MDSeq make this initial choice of study design less legitimate.

> *6. In Figure 3, "True DD" term is used. How are these classified? Further clarification is needed on the 'gold standard' for DD.*

Here, we refer to genes simulated to have a difference of dispersion between conditions and identified as differentially dispersed (DD) genes by the evaluated methods. We corrected the caption of figure 3 by replacing "True DD" by "Differentially dispersed genes correctly identified", which, we think, makes it clearer.

> *7. In lines 220-221, the number of non-DE genes are substantially different between the two methods. These numbers invariably impact the number of DD tests run. Please elaborate further on how this difference in number of non-DE genes impact the number of DD genes classified by the models.*

As mentioned in our response to your fifth comment, we changed the set of genes among which to look for differentially dispersed genes in the revised version of our manuscript and focused on the non-differentially expressed genes, as indeed this seems more relevant biologically to explore DD genes among non-DE genes. We chose MDSeq to filter out DE genes in order to take into account batch effects in the GLM it implements.

> *8. I suggest that if you plan on including discussion on single-cell datasets, some validation is required, especially for the claim that MDSeq may be more useful.*

We kept the discussion on single-cell datasets in the revised version of our manuscript as a perspective of application of differentially dispersed gene identification methods. We agree that maintaining the claim of using MDSeq for this type of data would require some validation, especially because of its poor performance in comparison with the newly included methods in our study. Thus, we removed this mension in the revised version of our manuscript.

> *9. The results from the TCGA DD analysis are extremely useful not only to establish the utility of the tools but also interpreting DD genes as having a role in robustness and regulation. The GO analysis and the interpretation seems very speculative at the moment. The discussion includes hand-picking a few pathways that showed up from the GO analysis. I suggest that the the biological findings be enhanced by analyzing a treatment dataset for one of the cancer types chosen from TCGA to further show how a potential 'therapeutic' can regulate the DD genes in a way opposite to that from cancer etiology.*

The goal of the application of the evaluated methods to TCGA datasets was to highlight the potential benefit of looking for genes with differences of dispersion in their expression. We try as far as possible to reduce speculation in the GO terms analysis by using redundancy reduction methods to ease the comparison of enriched GO terms. The top 40 representative terms and the p-values of their enrichment in each dataset are shown in Figure 6 and we were pleased to see them enriched in every cancer we consider. Therefore we think that the hypotheses we generated are interesting leads to explore further but we did not have time in the revision process to include any kind of validation.

Reviewer 3

Reviewer 3 did not make any comment or suggestion.

Changes to manuscript

Most of the changes made in the revised version of our manuscript relate to the inclusion of Levene's test [3], the analysis proposed by de Jong *et al* [1] and DiffDist [2] in the set of evaluated methods and, more generally, to the different comments made by the reviewers. We also changed the set of genes among which to look for differentially dispersed genes. We focused on the non-differentially expressed genes, since it is the set of genes of most interest among which to look for differentially dispersed genes, rather than extending the set to lowly differentially expressed genes according to the maximum mean fold changes tolerated by MDSeq. We think that this choice is more biologically relevant.

As a consequence, all the figures, supporting figures and supporting files take into account the aforementioned changes. No other changes were applied to figures 1, 2 and 6. In figure 3, we replaced the Venn diagram by an upset plot and focused on the errors of dispersion $\log_2$-fold change sign made by GAMLSS and DiffDist rather than differences between MDSeq and DiPhiSeq. In figure 4, we replaced the heatmap by a barplot for clarity. Similarly, we replaced barplots by upset plots in figure 5. The main changes made to the supporting information were the replacement of list of enriched GO terms in tabulated files by files storing complete figures on multiple pages for clarity. We also added the supporting figure 2 as an answer to the third comment of reviewer 2.

References

[1] T. V. de Jong, Y. M. Moshkin, and V. Guryev. Gene expression variability: the other dimension in transcriptome analysis. *Physiol Genomics*, 51(5):145–158, May 2019. [DOI:10.1152/physiolgenomics.00128.2018] [PubMed:30875273].

[2] A. G. K. Roberts, D. R. Catchpoole, and P. J. Kennedy. Identification of differentially distributed gene expression and distinct sets of cancer-related genes identified by changes in mean and variability. *NAR Genom Bioinform*, 4(1):lqab124, Mar 2022. [PubMed Central:PMC3401966] [DOI:10.1093/nargab/lqab124] [PubMed:35047816].

[3] Howard Levene. *Robust Tests for Equality of Variances*, pages 278–292. Stanford University Press, Stanford, Calif, 1960.

[4] M. A. Dillies, A. Rau, J. Aubert, C. Hennequet-Antier, M. Jeanmougin, N. Servant, C. Keime, G. Marot, D. Castel, J. Estelle, G. Guernec, B. Jagla, L. Jouneau, D. LaloÃń, C. Le Gall, B. SchaÃńffer, S. Le Crom, M. Guedj, and F. JaffrÃĭzic. A comprehensive evaluation of normalization methods for Illumina high-throughput RNA sequencing data analysis. *Brief Bioinform*, 14(6):671–683, Nov 2013. [DOI:10.1093/bib/bbs046] [PubMed:22988256].
